# Literature-derived serum miRNA signatures associated with cognitive decline in Alzheimer’s disease: integrated analysis and machine learning-based diagnostic modeling

**DOI:** 10.1186/s13195-026-02048-x

**Published:** 2026-04-20

**Authors:** Zhiyan Chen, Yadi Liu, Huan Wang, Ke Liu, Yutong Li, Xiaohua Hu, Rui Li, Linjuan Sun

**Affiliations:** 1 Beijing University of Chinese Medicine, Beijing, China; 2 Academy of Chinese Medical Sciences, Xiyuan Hospital, China, Beijing, China

## Abstract

**Objective:**

Because the clinical diagnosis of Alzheimer’s disease (AD) still relies largely on cognitive decline and A/T/N biomarkers remain costly and invasive, we aimed to identify literature-derived serum microRNAs (miRNAs) associated with cognitive function in AD and to re-evaluate them in a large public cohort (GSE120584) as potential adjunctive diagnostic biomarkers.

**Methods:**

We systematically searched Chinese- and English-language databases for studies reporting serum miRNA expression in patients with AD and its association with cognitive scale scores. Functional target genes were retrieved from miRTarBase, protein–protein interaction (PPI) networks were constructed using STRING, and functional modules were identified with the Cytoscape plug-in MCODE. Enrichment analyses were then performed for Gene Ontology (GO), KEGG, Reactome, and Hallmark gene sets. Differential expression analysis of GSE120584 was conducted using limma, and partial correlations between miRNA expression and age, sex, and apolipoprotein E ε4 (APOE ε4) allele count were calculated. Based on the correlation structure and nested cross-validation, optimal miRNA combinations were selected, and multiple diagnostic models were developed using age and sex as baseline clinical predictors. Model performance was evaluated using receiver operating characteristic (ROC) and precision-recall (PR) curves, calibration curves, and decision curve analysis (DCA).

**Results:**

Twenty-three publications including 2,458 patients with AD and 2,139 controls were ultimately included. Twenty-two differentially expressed serum miRNAs were identified, of which 15 were positively and 7 were negatively correlated with Mini-Mental State Examination (MMSE) scores. Target genes of positively correlated miRNAs were enriched in PI3K/AKT/mTOR, Wnt, and TNF-α/NF-κB signaling pathways, whereas target genes of negatively correlated miRNAs were mainly involved in cell cycle regulation, the G2/M checkpoint, and oxidative stress responses. After matching literature-derived miRNAs with GSE120584 and expanding the candidate miRNA set, 25 significantly differentially expressed miRNAs were identified. Correlation-network analysis combined with nested cross-validation indicated that the minimal miRNA signatures with optimal diagnostic value were miR-211-5p alone (K1) and the three-miRNA panel miR-211-5p, miR-128–1-5p, and miR-128-3p (K3). When age and sex were added, the “K3 + age + sex” model showed the best performance (area under the curve [AUC] = 0.838, average precision [AP] = 0.934, Brier score = 0.162), yielding the highest sensitivity (0.563) and the best positive predictive value (PPV = 0.954) at specificity ≥ 0.90.

**Conclusion:**

By integrating published evidence with re-evaluation in a public serum cohort, we found that adding miR-211-5p and a miR-128-related panel to age- and sex-based models provided a modest but consistent improvement in diagnostic discrimination for AD. These miRNAs may serve as adjunctive peripheral blood biomarkers, although their clinical utility requires confirmation in independent external cohorts.

**Supplementary Information:**

The online version contains supplementary material available at 10.1186/s13195-026-02048-x.

## Introduction

Alzheimer's disease (AD) is the most prevalent neurodegenerative disorder among older adults. Clinically, AD presents with cognitive impairment, most notably progressive memory loss [1], and is the most common cause of dementia [2]. With population aging worldwide, the prevalence of AD continues to rise [3]. For example, by 2050, the number of individuals aged 65 years and older living with AD in the United States is projected to reach 13.8 million [4], while China is expected to have approximately 15.07 million people with AD among its population aged 60 years and above [5]. AD is also a leading cause of death among older adults. As the disease progresses, patients often develop changes in mood, personality, and behavior and gradually require assistance with activities of daily living, thereby imposing a substantial socioeconomic burden on patients, families, and society [6].

At present, the clinical diagnosis of AD still relies largely on clinical presentation and cognitive assessment, most commonly with instruments such as the Mini-Mental State Examination (MMSE) and Montreal Cognitive Assessment (MoCA) [7]. However, biological changes in AD begin years to decades before symptom onset, a period now referred to as preclinical AD [8]. According to the NIA-AA framework, the core pathological features of AD include amyloid-β deposition, pathologic tau, and neurodegeneration [9]. Neuropathological studies suggest that AD-related changes begin in the transentorhinal region and then spread to the hippocampus and medial temporal cortex before becoming more widespread [10].

In 2018, the National Institute on Aging-Alzheimer’s Association (NIA-AA) proposed the A/T/N framework for AD biomarkers, classifying them into amyloid-β (A), pathologic tau (T), and neurodegeneration or neuronal injury (N). Beyond the A/T/N framework, increasing attention has been directed toward microRNAs (miRNAs) as peripheral biomarkers and potential regulators of AD-related pathways. miRNAs are short non-coding RNAs that post-transcriptionally regulate gene expression by binding to the 3′ untranslated region of target mRNAs, thereby repressing translation or promoting mRNA degradation [11, 12]. Because they can be readily measured in peripheral blood, miRNAs have been investigated as minimally invasive biomarkers for diagnosis and prognostic assessment in AD and in other conditions, including breast cancer, lung cancer, and obesity [13]. Current research indicates that miRNA dysregulation is present in the blood, cerebrospinal fluid, and central nervous system of patients with AD and is involved in multiple pathological mechanisms, including chronic neuroinflammation, oxidative stress, and abnormal cell cycle progression [11].

Previous studies have suggested that peripheral blood miRNAs may serve as candidate biomarkers for AD [14, 15]. However, most reported candidates have been identified either through agnostic differential-expression screening across all detectable miRNAs or through network- and pathway-based mining of public datasets [16]. Consequently, many candidate miRNAs are not well supported by published clinical studies demonstrating both differential serum expression and direct associations with cognitive performance in AD, and their mechanistic relevance to AD pathology remains insufficiently defined. Although large observational cohorts have also examined serum miRNAs related to cognition [17], systematic integration of clinically reported cognition-associated serum miRNAs in AD, together with evaluation of their diagnostic relevance and biological context, is still lacking.

To address this gap, we first identified serum miRNAs that had been reported to be differentially expressed in AD and associated with cognitive function through a literature-based screening process. These miRNAs were then subjected to target gene analysis, and their expression patterns and diagnostic value for AD were further evaluated in a public serum miRNA dataset (GSE120584), with the aim of bridging published evidence, reproducibility in public cohorts, and clinical interpretability.

## Methods

This study consisted of two sequential stages. In the first stage, candidate serum miRNAs associated with cognitive function in AD were identified through systematic literature searching and integrated screening of the retrieved studies. In the second stage, the expression direction and diagnostic utility of these candidate miRNAs were re-evaluated in the large public cohort GSE120584.

### Literature search

We systematically searched four Chinese databases (CNKI, Wanfang, VIP, and SinoMed) and four English-language databases (PubMed, Cochrane Library, Web of Science, and Embase) from database inception to November 2024 for studies reporting miRNA expression in patients with AD. A combination of subject headings and free-text keywords was used. Records were imported into EndNote X9 (version 3.3) for deduplication and screening. The full electronic search strategies for each database are provided in [Supplementary Material 1].

### Inclusion and exclusion criteria

We included case–control studies comparing serum miRNA expression between patients with AD and cognitively healthy controls.

Studies were eligible if they met all of the following criteria: (**1**) AD was diagnosed using clearly defined criteria; (**2**) cognitive function was assessed with at least one standardized scale, including MMSE, MoCA, or Alzheimer’s Disease Assessment Scale-Cognitive Subscale (ADAS-Cog); (**3**) serum miRNA expression was measured by quantitative real-time polymerase chain reaction (qRT-PCR); and (**4**) associations between miRNA expression and cognitive-scale scores in patients with AD were analyzed using Pearson’s or Spearman’s correlation, with statistical significance defined as *p* < 0.05 [18, 19].

Studies were excluded if they met any of the following criteria: (**1**) miRNA expression was not assessed in the control group; (**2**) miRNA expression was not significantly correlated with cognitive scale scores according to Pearson or Spearman correlation analysis (*p* > 0.05); (**3**) the difference in miRNA expression between the AD and control groups was not statistically significant (*p* > 0.05); or (**4**) the sample size of either the AD group or the control group was fewer than 60 participants. The literature screening protocol of this study was registered in PROSPERO (CRD42024605700).

### Data extraction

Two independent reviewers (Li Yutong and Hu Xiaohua) first screened the titles and abstracts of all records retrieved from the databases and excluded clearly irrelevant studies, including animal experiments, reviews, pharmacological studies, and randomized controlled clinical trials. Potentially eligible articles then underwent full-text review according to the predefined inclusion and exclusion criteria. Any discrepancies in the assessment of study eligibility were resolved through discussion with a third reviewer (Liu Yadi).

For each included study, we extracted baseline information, including AD diagnostic criteria, sample sizes and ages of the AD and control groups, differentially expressed miRNAs, cognitive scale scores, and the results of correlation analyses. The methodological quality of the case–control studies was assessed using the relevant items of the Newcastle–Ottawa Scale (NOS). Differentially expressed miRNAs that were significantly associated with cognitive performance in patients with AD were extracted for subsequent analyses. For simplicity, the prefix “hsa-” was omitted from miRNA names throughout the Results section.

### Selection of miRNA target genes, construction of PPI networks, and enrichment analysis

Experimentally validated target genes of the included miRNAs were retrieved from the miRTarBase database (Version 9.0) [20]. Only target genes with a support type classified as Functional MicroRNA Target Interaction (Functional MTI) were retained, and all genes were annotated using HGNC gene symbols. According to the direction of the correlation between miRNA expression and cognitive function in patients with AD, the included miRNAs were categorized as positively or negatively correlated. The experimentally validated target genes of positively correlated miRNAs, negatively correlated miRNAs, and the intersection of these two gene sets were then subjected, in turn, to the following analyses:

1. Protein–protein interaction (PPI) networks for the three gene sets were constructed using the STRING online database (https://string-db.org/) with a medium confidence score threshold (> 0.4) [21].
2. The PPI network was analyzed using Cytoscape software (version 3.10.1) [22]. Core modules were identified with the Molecular Complex Detection (MCODE) plugin [23] using the following parameters: degree cutoff = 2, node score cutoff = 0.2, K-core = 2, and maximum depth = 100.
3. Enrichment analyses were performed for the three target-gene sets and for the top-ranked modules identified by MCODE analysis. All enrichment analyses were conducted in R (version 4.3.2) using the clusterProfiler, ReactomePA, and msigdbr frameworks, including GO analyses of Biological Process (BP) and Cellular Component (CC), Kyoto Encyclopedia of Genes and Genomes (KEGG) pathway analysis, Reactome pathway analysis, and Hallmark gene set analysis. Multiple testing was adjusted using the Benjamini–Hochberg method to control the false discovery rate (FDR), with statistical significance defined as FDR < 0.05. For the enrichment analysis at the whole-set level of the three target-gene sets, the minimum gene set size (minGSSize) was set to 10; for the MCODE module-level analysis, given the smaller set sizes, minGSSize was relaxed to 3. Biological interpretation was provided only for terms meeting the significance threshold.

### Differential expression analysis and covariate sensitivity analyses in GSE120584

Differential expression analyses were performed in R (version 4.3.2) using GSE120584 [24]. Because the dataset was generated from serum miRNA microarrays, analyses were conducted with the limma package [25], which fits linear models with empirical Bayes shrinkage of variance estimates. After log₂ transformation, quantile normalization was applied across samples. Low-variability features were then filtered on the basis of row standard deviation (rowSD), using the 25th percentile (Q25) as the threshold to improve statistical power and reduce the burden of multiple testing.

Differential expression analysis employed the limma linear model with an empirical Bayes variance-shrinkage framework. To minimize the influence of hyper-variable features and array-quality variation, robust hyperparameter estimation was applied using eBayes(trend = TRUE, robust = TRUE). Because GSE120584 includes age, sex, and apolipoprotein E ε4 (APOE ε4) allele count [24], the arrayWeights function was used to assign empirical weights to each sample [26]. Multiple testing was adjusted using the Benjamini–Hochberg procedure to control the false discovery rate (FDR), with statistical significance defined as FDR < 0.05 [27].

To evaluate the effect of covariate modeling strategies, two sensitivity analyses were conducted: (**1**) modeling APOE ε4 as a continuous variable (0/1/2 counts); and (**2**) excluding APOE ε4 (adjusting only for age and sex). The Jaccard index was used to assess the overlap of significant miRNAs between models, and Spearman correlation coefficients were calculated for log₂FC values of shared miRNAs to quantify the consistency of the results.

For literature-derived miRNAs that were not directly matched in GSE120584, those without explicit annotation of arm origin were further searched in GSE120584 for miRNAs from the same family or potentially corresponding arm-specific forms, which were then additionally included in the exploratory analysis.

### Directionality-consistency testing, set-level bias assessment, and signature score construction

Based on the correlation between miRNA expression and cognitive scale scores in patients with AD, the included miRNAs were categorized as “positively correlated” or “negatively correlated.” These classifications were integrated with the differential expression analysis of the corresponding miRNAs.

1. Directional consistency was evaluated according to the expected trend—positively correlated miRNAs were expected to be downregulated in AD, whereas negatively correlated miRNAs were expected to be upregulated. Statistical significance was determined after multiple comparison correction (BH-FDR < 0.05).
2. To assess systematic directional bias across all miRNAs, statistical measures were ranked (preferring t-statistics and using log₂FC when unavailable), and one-sided tests were performed using limma’s mean-rank gene set test (geneSetTest) according to the expected direction [28].
3. To confirm the robustness of the observed directional bias at the individual level, one-tailed sign tests and one-sample rank-sum tests were conducted on log₂FC values within each set [29, 30].
4. After row-wise z-score normalization within each set, the negatively correlated set was assigned a weight of + 1 and the positively correlated set a weight of − 1. A weighted mean was then calculated for each sample to generate the signature score. Between-group differences in this score were assessed using a linear model including diagnosis, age, sex, and APOE ε4 allele count [25, 28].

All subsequent analyses were performed using Python (version 3.11.8) [31].

### Covariate diagnostic performance and miRNA-covariate association analyses

Based on miRNA expression data from GSE120584, we evaluated the univariate diagnostic value of age, sex, and APOE ε4 allele count for AD using two complementary approaches. First, the area under the ROC curve (AUC) [32] and average precision (AP) [33] were calculated using the original variables as prediction scores. Second, robust small-sample estimates were obtained using stratified five-fold cross-validation [34]. For each training fold, prediction probabilities were generated from a univariate logistic regression model. Thresholds were selected on the training folds using Youden’s index [35] and then fixed on the corresponding validation fold to evaluate sensitivity, specificity, positive predictive value, negative predictive value, and accuracy. In parallel, AUC and AP on the validation folds were recorded, and the mean and standard deviation across folds were subsequently summarized.

We then analyzed associations between the included miRNAs and age, sex, and APOE ε4 allele count. Spearman rank correlation was used for the continuous covariates (age and APOE ε4 allele count), whereas point-biserial correlation was used for the binary covariate sex [36]. P values were adjusted for multiple testing at the miRNA level using the Benjamini–Hochberg method [27]. To account for potential confounding and interactions among covariates, we additionally calculated partial Spearman correlations [37] and partial rank correlation coefficients (PRCCs) [38]. All variables were first rank-transformed, after which each miRNA and the target covariate were residualized against the remaining covariates. Pearson correlations were then calculated on the residuals, and two-sided P values and FDR-adjusted q values were obtained.

### Cluster-constrained feature selection and cross-validated miRNA panel construction

Because correlations among miRNAs may lead to model instability and interpretive redundancy, we first calculated pairwise Spearman rank correlations (ρ) for the included miRNAs and generated a correlation matrix using pairwise complete observations to handle missing data [27]. Edges with |ρ|≥ 0.70 were extracted to construct an undirected correlation network, and miRNA clusters were defined as connected components. The primary analysis summarized cluster structure at |ρ|≥ 0.80, while cluster mappings retaining singleton nodes were also preserved for comparison. To further assess potential confounding, subsequent cluster interpretation and representative-miRNA selection were informed by the results of the partial Spearman analyses described in Sect. " Covariate diagnostic performance and miRNA-covariate association analyses", in which associations of miRNAs with age, sex, and APOE ε4 were evaluated using a rank-based residual approach (rank transformation followed by residualization) [19, 38, 39]. At the correlation-network level, we applied a cluster-constrained selection strategy, under which no more than one representative miRNA was retained from each cluster unless an additional within-cluster candidate produced a greater cross-validated incremental gain in discrimination (ΔAUC ≥ 0.010), thereby reducing feature redundancy and improving panel robustness. For the largest cluster, we additionally derived a module eigengene (ME), defined as the first principal component obtained after column-wise z-score normalization and principal component analysis (PCA) within the cluster, to summarize overall cluster expression and describe its associations with age and disease status [40].

We then screened miRNA combinations for diagnostic value beyond the age-and-sex baseline. Within each outer training fold, redundancy-reduced representative miRNAs were ranked according to forward gain, defined as the increase in cross-validated AUC produced by adding a candidate to the baseline model or the current set. A minimum gain threshold of ΔAUC ≥ 0.005 was prespecified; candidates from the same cluster were required to meet a stricter threshold of ΔAUC ≥ 0.010, while the one-representative-per-cluster rule was maintained throughout the screening process.

Across outer folds and repeated runs of the configuration, selected results within each “outer-fold × model” combination were first deduplicated, and then selection frequencies were aggregated. This procedure yielded a frequency panel after cluster-level deduplication. For each K = 1…K_max (where K_max was determined by the length of this panel), K-AUC curves were sequentially evaluated and plotted. Following the strict “1-SE rule” [41], we selected the minimum K that satisfied the performance threshold to obtain a more parsimonious combination with near-optimal discrimination. In addition, univariate fivefold cross-validation analyses were performed for all miRNAs in the GSE120584 dataset to evaluate the diagnostic value of each miRNA individually.

### Cross-validated classifier comparison with threshold optimization and decision curve analysis

Because age and sex are routinely available in clinical practice, they were used as the baseline clinical predictors throughout the analysis.

We constructed six diagnostic models based on the baseline, all included miRNAs (M1), and the selected combination of miRNAs with the highest diagnostic value (M2). These models were: M1, M1 + age, M1 + age + sex; M2, M2 + age, and M2 + age + sex. All models underwent performance estimation via 5 × 5 nested cross-validation (nested CV) under strictly reproducible settings (single-threaded, fixed random seed) [40]. The inner layer was used for hyperparameter tuning, whereas the outer layer provided an assessment of generalization performance, thereby minimizing model selection bias and overfitting risk.

The primary classifier was elastic net logistic regression [42], with support vector machines using linear and radial basis function kernels (linear SVM [43] and RBF-SVM [44, 45]), random forest (RF) [46], and XGBoost [47] as comparator algorithms. Continuous features [48] were processed uniformly within the training pipeline using median imputation followed by standardization, and categorical features were binarized. Class imbalance [49] was handled through class-weight balancing and, when applicable, additional sample weighting. Out-of-fold (OOF) probabilities from the outer-layer cross-validation were used as unbiased performance estimates, and the primary evaluation metrics were the area under the receiver operating characteristic curve (AUC), average precision (AP), and Brier score.

**Fig. 1:**
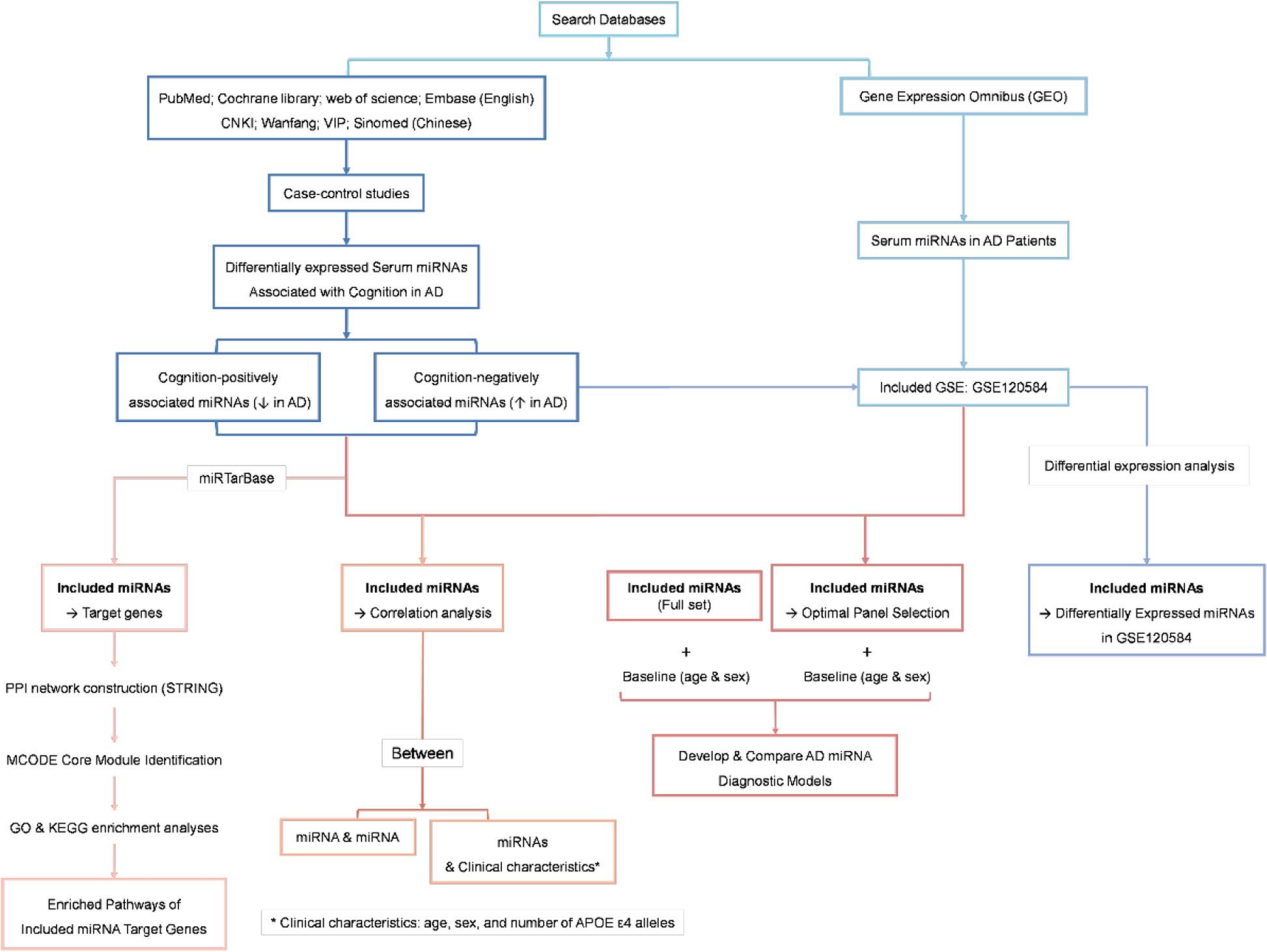
Overall study workflow

To provide clinically interpretable classification thresholds, we derived optimal cut-offs using Youden’s index and additionally reported model performance under prespecified operating points of specificity ≥ 0.90 or sensitivity ≥ 0.90. To characterize the net benefit of different models in clinical decision-making, we plotted and compared decision curves (decision curve analysis, DCA) based on out-of-sample probabilities [50], reporting net benefit at each threshold probability (pt) and the relative advantage interval over “treat-all” and “treat-none” strategies [51, 52]. The study workflow is shown in (Fig. 1).

## Results

### Literature search findings

**Fig. 2:**
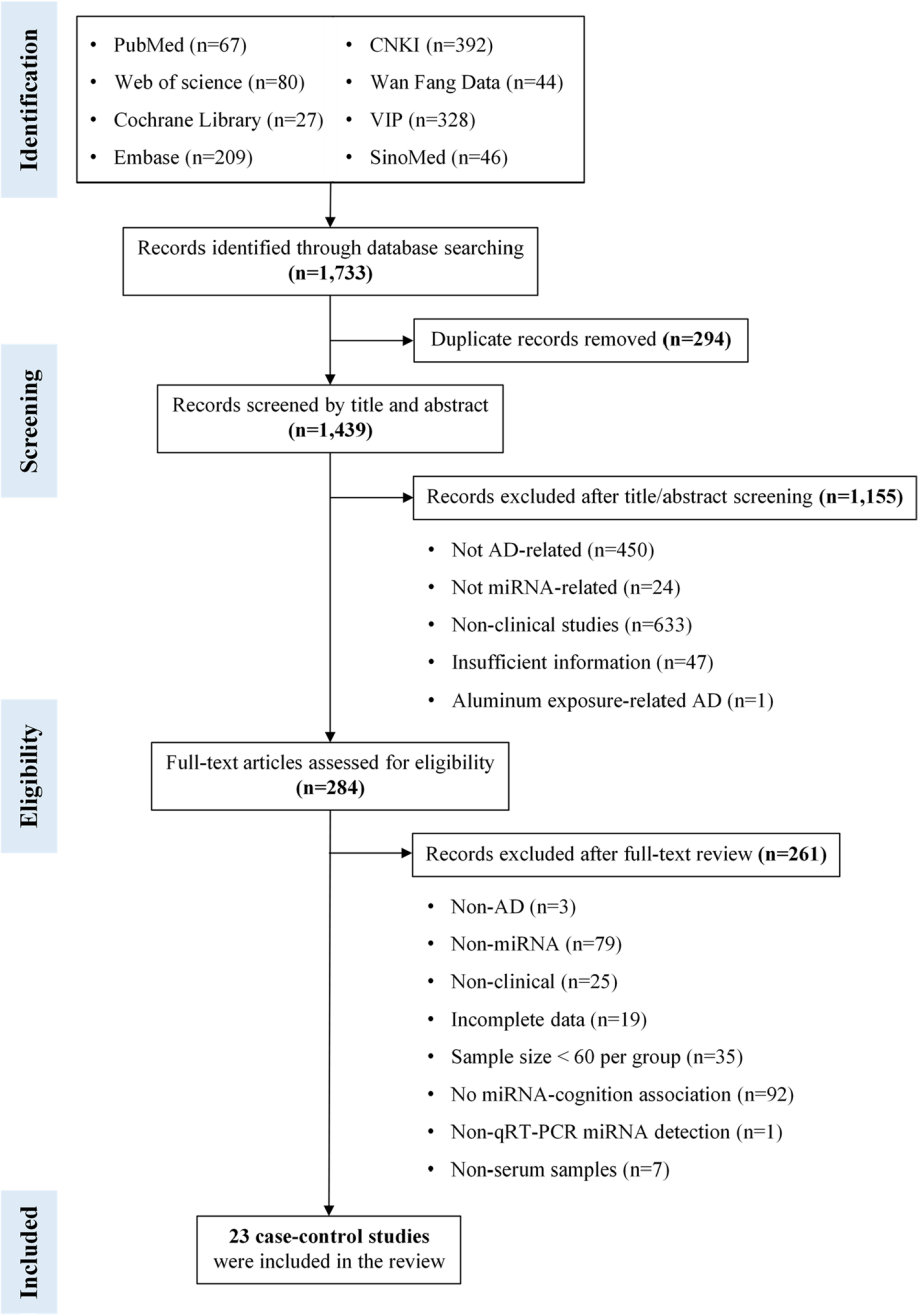
Flow diagram of the literature search and study selection process

A total of 1,733 records were identified through database searching. After screening, 23 case–control studies were included (Fig. 2). NOS quality assessment for the included studies is summarized in [Supplementary Material 2]. Across these studies, 2,458 patients with AD and 2,139 controls were enrolled. All studies used MMSE to assess cognitive function, and baseline characteristics are presented in Table 1. In total, 22 miRNAs that were differentially expressed in serum from patients with AD and associated with cognitive function were identified (Table 2). The differential expression patterns of these miRNAs in the included studies, together with their correlations with cognitive scale scores, are summarized in [Supplementary Material 3].

**Table 1:** Characteristics of the included case–control studies

Study	Number of participants		Age (years)		Sex (male/female)		MMSE score	
	**AD**	**CON**	**AD**	**CON**	**AD**	**CON**	**AD**	**CON**
Xiao JianTing, 2014 [53]	75	85	75.28 ± 6.26	75.46 ± 6.21	35/40	40/35	13.27 ± 2.53	≥ 28
Tan, L.,2014 [54]	158	155	77.43 ± 7.40	76.86 ± 7.00	78/80	70/85	10.45 ± 2.21	28.31 ± 0.12
Jia LiHua, 2016 [55]	84	62	81.36 ± 13.25	76.23 ± 19.16	62/22	41/21	18.3 ± 5.2	NA
Qin Yang, 2019 [56]	98	105	76.46 ± 5.87	75.38 ± 7.11	60/45	54/44	20.48 ± 5.42	29.00 ± 0.64
Cao F, 2020 [57]	108	93	71.50 ± 4.76	71.10 ± 4.88	59/49	52/41	18.13 ± 3.67	27.09 ± 1.49
Zhao, Xiaohua, 2020 [58]	68	75	70.12 ± 2.09	69.47 ± 1.98	31/37	34/41	15.48 ± 1.68	28.03 ± 1.52
Lin Ruidian, 2021 [59]	86	90	72.2 ± 5.6	71.8 ± 6.0	48/38	45/45	20.51 ± 1.95	28.05 ± 1.08
Meng Kaitao, 2021 [60]	200	207	72.89 ± 5.47	73.25 ± 5.07	119/81	109/98	*Mild AD:20.34 ± 2.16 Moderate AD:16.26 ± 1.68 Severe AD:14.19 ± 1.03	NA
Qiao Weidong, 2021 [61]	150	150	75.8 ± 7.6	74.0 ± 8.3	76/74	77/73	16.78 ± 2.61	28.76 ± 2.84
Zhang Qun, 2021 [62]	110	100	70.61 ± 2.82	70.93 ± 3.21	64/46	52/48	17.33 ± 3.96	27.40 ± 1.62
Zhou Yulei, 2021 [63]	138	64	65.25 ± 8.14	64.88 ± 7.36	76/62	35/30	18 (15,20)	29 (27,30)
Dong LiHua, 2021 [64]	121	86	*Mild AD:70.5 ± 6.2 Moderate AD:69.6 ± 7.3 Severe AD:71.7 ± 6.4	69.0 ± 5.8	Mild AD:16/15 Moderate AD:28/24 Severe AD:17/21	45/41	*Mild AD:24.1 ± 1.3 Moderate AD:15.5 ± 2.6 Severe AD:7.0 ± 3.7	28.9 ± 0.9
Liu Qingling, 2021 [65]	110	60	72.69 ± 6.64	72.52 ± 7.57	59/51	37/23	16.05 ± 2.69	28.7 ± 0.89
Liu, L., 2021 [66]	104	94	65.43 ± 6.34	66.09 ± 6.22	57/47	46/48	15.8 ± 2.69	25.64 ± 1.51
Zhang, H., 2021 [67]	110	100	70.89 ± 4.09	71.64 ± 4.00	47/63	46/54	17.39 ± 3.89	27.38 ± 1.66
Zhang, M., 2021 [68]	117	106	72.57 ± 8.13	73.15 ± 9.29	67/50	61/45	16.88 ± 2.04	26.81 ± 2.19
Deng Tianling, 2022 [69]	110	110	69.40 ± 6.92	68.76 ± 5.38	62/48	55/55	18.24 ± 2.38	28.06 ± 1.04
Wang Wei, 2022 [70]	65	60	75.61 ± 5.13	74.82 ± 5.27	28/37	31/29	△Mild AD:23.47 ± 3.19 Moderate AD:16.24 ± 2.55 Severe AD:6.76 ± 1.31	NA
Zhou Weihua, 2022 [71]	95	63	68.28 ± 8.05	67.92 ± 7.12	32/63	20/43	22.01 ± 1.65	28.12 ± 1.03
He Xia, 2023 [72]	103	50	66.54 ± 5.87	65.18 ± 5.52	63/40	31/19	16 (12,19)	28 (27,29)
Wang, T., 2023 [73]	92	68	70.12 ± 8.56	69.50 ± 6.55	49/43	37/31	16.76 ± 6.66	NA
He Lijie, 2024 [74]	66	66	#Mild AD:71.31 ± 7.52 Moderate & Severe AD:72.57 ± 6.83	69.45 ± 7.36	Mild AD:23/18 Moderate & Severe AD:15/10	39/27	#Mild AD:21 (17,25) Moderate & Severe AD:9 (7,11)	27(21,34)
Wang Pengfei, 2024 [75]	90	90	72.25 ± 5.32	72.55 ± 5.24	51/39	50/40	*Mild AD:22.54 ± 1.41 Moderate AD:19.35 ± 1.01 Severe AD:16.23 ± 1.00	NA

CON = control group; AD participants were grouped according to the Clinical Dementia Rating (CDR) scale [76] and MMSE scores: * Mild AD: CDR = 1; Moderate AD: CDR = 2; Severe AD: CDR = 3 [77]; △ mild AD(MMSE 21–26); moderate AD (MMSE = 10–20); severe AD (MMSE = 0–9) [70]; # mild AD(CDR ≤ 1); moderate & severe AD(CDR > 1) [74]

**Table 2:** Literature-derived serum miRNAs associated with cognitive function in AD

Correlation with cognitive function	miRNA
positively correlated miRNAs (n = 15)	miR-133b, miR-137, miR-148a-3p, miR-193a-3p, miR-202, miR-211, miR-222, miR-223, miR-26b, miR-27a-3p, miR-320a, miR-331-3p, miR-340-5p, miR-511-3p, miR-342-3p
negatively correlated miRNAs (n = 7)	miR-128, miR-138, miR-24-3p, miR-28-3p, miR-9, miR-98-5p, miR-142-5p

The clinical characteristics of participants in GSE120584 are presented in (Table 3). The AD and control groups differed substantially in baseline characteristics.

**Table 3:** Clinical characteristics of participants in GSE120584

Characteristic	AD	Control	p-value and effect size
n	1021	288	NA
Age	80.0 [76.0, 84.0]	71.0 [67.0, 76.0]	*p* < 0.01, SMD = 1.215
Sex (male/female)	307/714	151/137	*p* < 0.01, Cramér's V = 0.194
APOE ε4 (0/1/2)	577/376/68	238/46/4	*p* < 0.01, Cramér's V = 0.225

Age was compared between groups using the Mann–Whitney U test (skewness: AD group |0.395|, control group |0.545|). Sex and APOE ε4 allele count were compared using Fisher’s exact test

### Construction of miRNA target gene PPI networks and MCODE module analysis

**Fig. 3:**
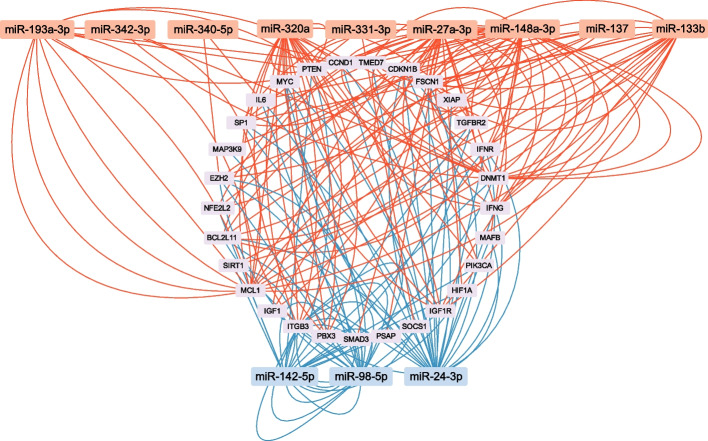
miRNA–target gene interaction network for target genes shared by positively and negatively correlated miRNAs. Note: miRNAs shown in red were positively correlated with MMSE scores, whereas those shown in blue were negatively correlated

Using miRTarBase (https://mirtarbase.cuhk.edu.cn/) [20], we retrieved experimentally validated target genes and retained only those annotated as Functional miRNA Target Interactions (Functional MTIs). Such validated target genes were available for 10 miRNAs in the positively correlated group and 4 miRNAs in the negatively correlated group Fig. 3.

Because the two groups shared some target genes, the validated targets were divided into three subsets: (1) shared target genes of positively and negatively correlated miRNAs (n = 29); (2) target genes unique to positively correlated miRNAs (n = 259); and (3) target genes unique to negatively correlated miRNAs (n = 101). STRING was then used to construct PPI networks for each subset, and the resulting networks were imported into Cytoscape for MCODE-based module detection. Module information and visualizations are presented in Tables 4 and 5.

**Table 4:**
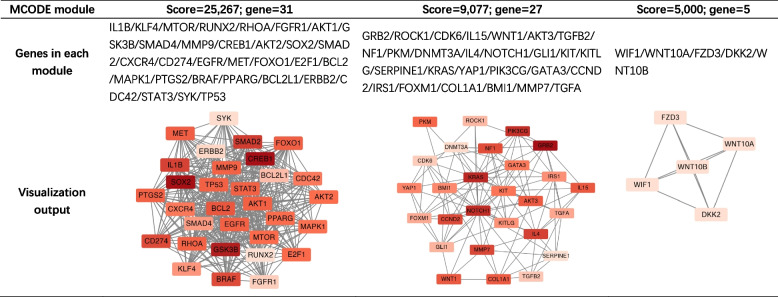
MCODE modules identified from target genes of positively correlated miRNAs

**Table 5:**
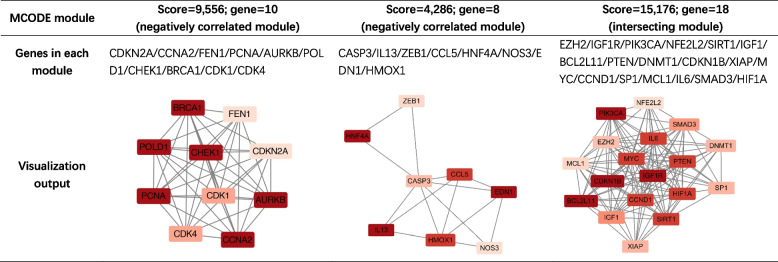
MCODE modules identified from target genes of negatively correlated and intersecting miRNAs

### Enrichment analysis of miRNA target genes

Enrichment analyses were performed for the target genes of the three miRNA groups and for each MCODE module. Overall, the differences in MCODE modules among target genes across groups were relatively minor. Pathways supported by an insufficient number of genes are therefore not discussed in detail here. Comprehensive enrichment results are provided in [Supplementary Material 4].

**Fig. 4:**
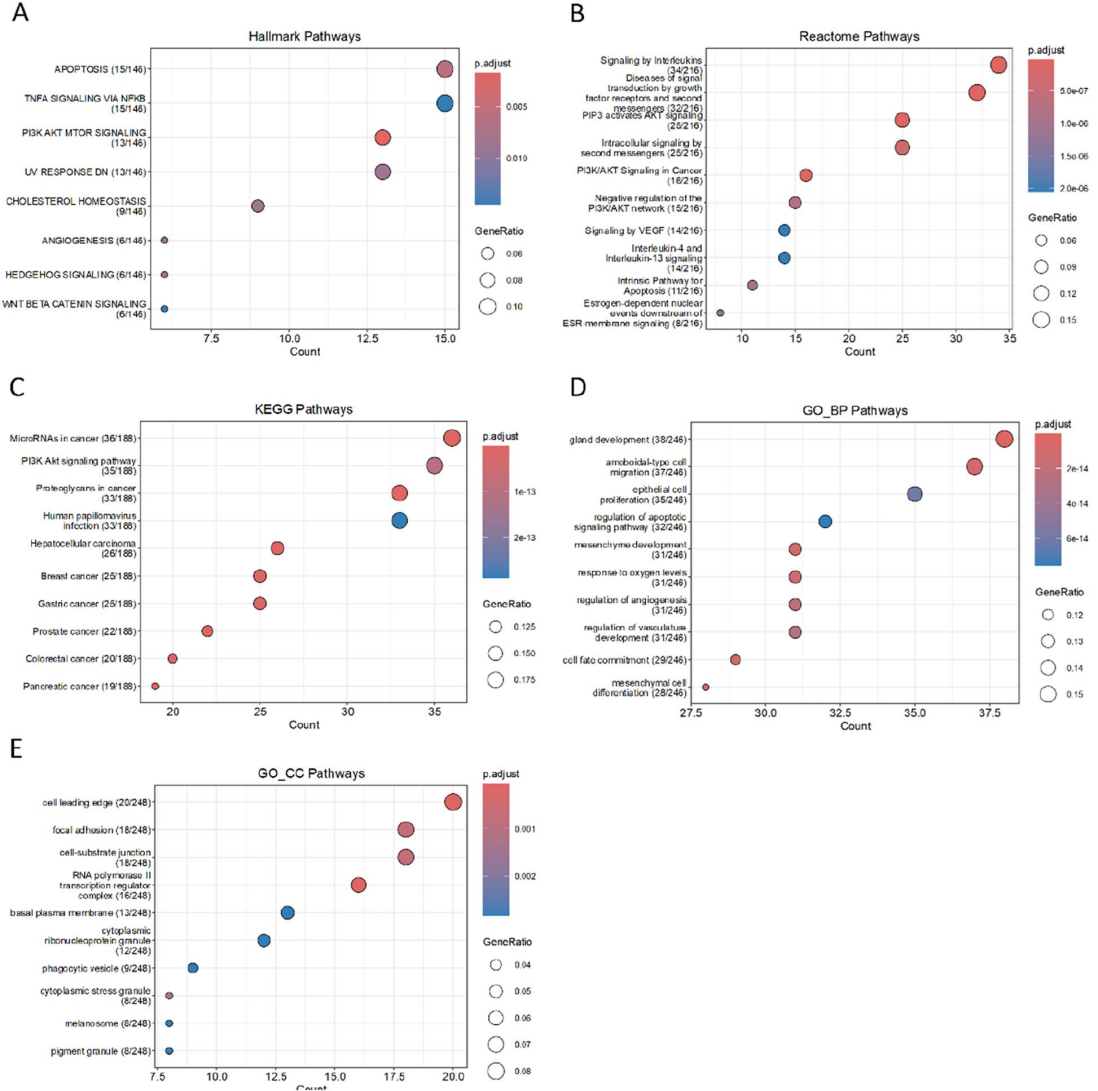
Functional enrichment analysis of target genes of positively correlated miRNAs

The enrichment results for positively correlated miRNA target genes are shown in (Fig. 4). In Hallmark analysis, these genes were significantly enriched in apoptosis and several signaling pathways, including the PI3K/AKT/mTOR, Wnt/β-catenin, and TNF-α/NF-κB pathways, as well as pathways related to cellular stress (UV_RESPONSE). Reactome analysis indicated that many target genes were enriched in interleukin signaling pathways, particularly interleukin-4 (IL-4) and interleukin-13 (IL-13), as well as in growth factor receptor-mediated signaling, second messenger-mediated signaling, PI3K/AKT-related pathways, and negative regulation of the PI3K/AKT network; these genes were also associated with vascular endothelial growth factor (VEGF) signaling and estrogen receptor-membrane signaling. KEGG analysis showed enrichment not only in cancer- and virus-related pathways, but also in focal adhesion and multiple signaling pathways, including PI3K/AKT, FoxO, and MAPK. GO analysis demonstrated that, in the Cellular Component (CC) category, these genes were enriched in focal adhesion and other cell migration-related components, including the cell leading edge, cell-substrate junction, basal plasma membrane, and RNA polymerase II transcription regulator complex. In the BP category, enrichment was observed in pathways such as regulation of angiogenesis, regulation of vasculature development, epithelial cell proliferation, and regulation of apoptotic signaling pathways.

Enrichment analysis was also performed for the three MCODE modules derived from positively correlated miRNAs.

1. Module 1 (score = 25,267, 31 genes) showed enrichment patterns that were largely consistent with the overall miRNA enrichment results. In the Hallmark gene sets, the target genes were enriched in activation of the PI3K/AKT/mTOR and KRAS signaling pathways. Reactome analysis further indicated enrichment in PI3K/AKT-related pathways, growth factor receptor signaling pathways, second messenger-mediated signal transduction, and interleukin signaling pathways. Additional enrichment was observed in endogenous apoptotic signaling and estrogen-related pathways. GO biological process (GO-BP) analysis revealed associations with glial cell differentiation (gliogenesis) and T-cell lineage commitment (T-cell differentiation), whereas GO cellular component (GO-CC) analysis indicated enrichment in the RNA polymerase II transcription regulator complex and multiple cell migration-related structures (cell leading edge, leading edge membrane, and ruffle membrane).
2. Module 2 (score = 9,077, 27 genes) showed, in the Hallmark analysis, enrichment of a subset of genes in cellular stress-related gene sets (UV_RESPONSE) and the Wnt/β-catenin signaling pathway. Reactome analysis again demonstrated enrichment in PI3K/AKT-related pathways, as well as growth factor receptor-mediated and second messenger-mediated signal transduction pathways. GO-BP terms were predominantly related to epithelial cell migration and proliferation, as well as Ras protein signaling pathways. GO-CC analysis indicated enrichment in transferase complexes acting on phosphate groups and secretion-related components (secretory granule lumen, cytoplasmic vesicle lumen, and vesicle lumen).
3. Module 3 (score = 5,000, 5 genes) contained relatively few genes; therefore, only Reactome and GO enrichment analyses were performed. All genes in this module were enriched in the Wnt signaling pathway in both Reactome and GO-BP analyses.

**Fig. 5:**
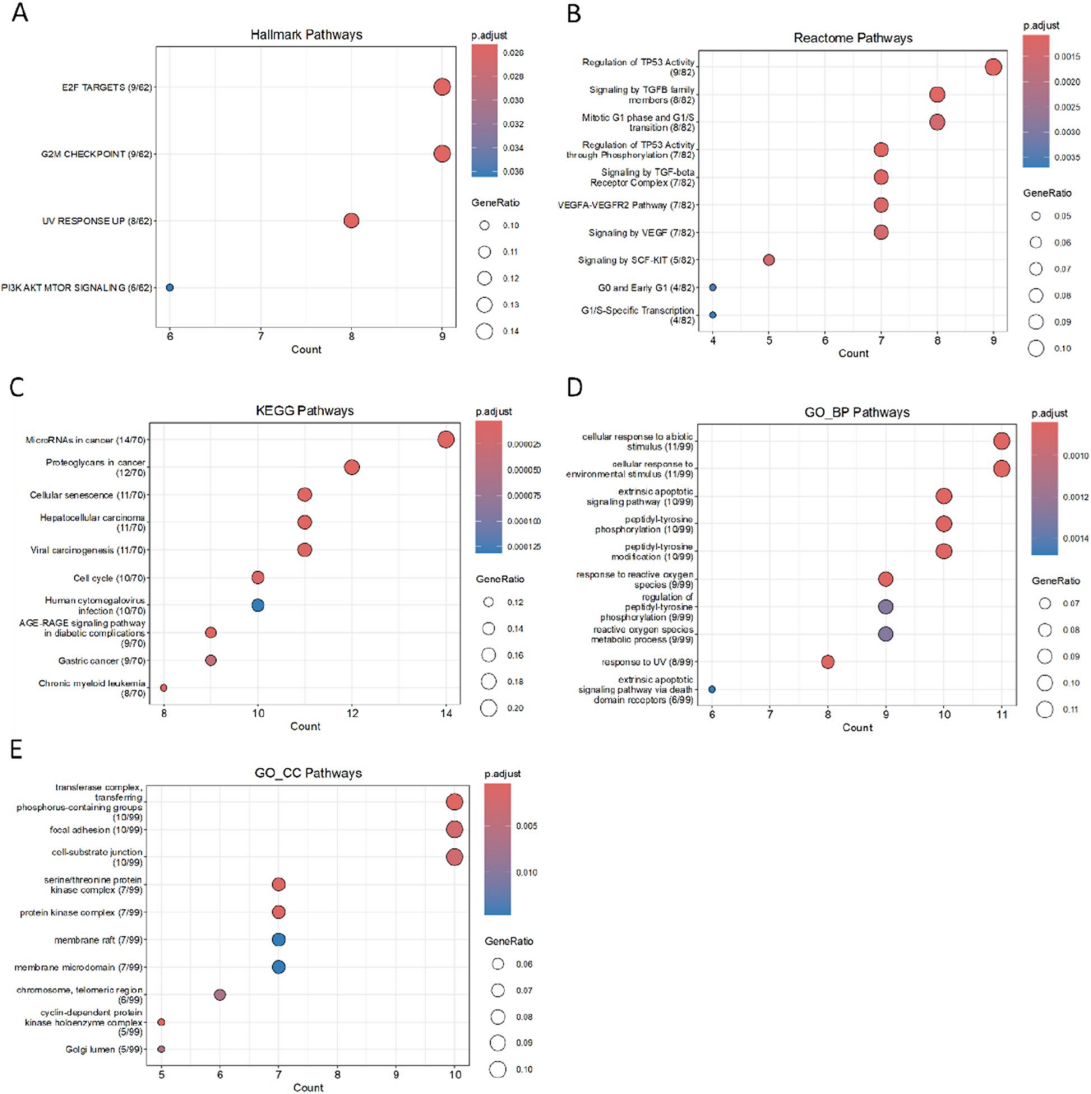
Functional enrichment analysis of target genes of negatively correlated miRNAs

The enrichment results for negatively correlated microRNA (miRNA) target genes are shown in (Fig. 5).

Hallmark analysis revealed that these target genes were associated with E2F transcription factors (E2F_TARGETS), cellular stress (UV_RESPONSE) and cell-cycle regulation (G2M_CHECKPOINT). Reactome analysis indicated that these target genes were involved in cell-cycle-related processes, including the mitotic G1 phase and G1/S transition, G0 and early G1, and G1/S-specific transcription. They were also associated with TGF-β signaling, TP53 phosphorylation, and VEGF signaling and its receptors.

Similarly, beyond general cancer- and leukemia-related pathways, KEGG analysis showed that negatively correlated miRNA target genes were enriched in pathways related to cellular senescence, the cell cycle and the AGE/RAGE signaling pathway.

In GO analysis, cellular component (GO-CC) terms were enriched for the transferase complex transferring phosphorus-containing groups and various protein kinase complexes, including the serine/threonine protein kinase complex and the cyclin-dependent protein kinase holoenzyme complex. In addition, enrichment was observed in cell migration-related structures, such as focal adhesions, cell-substrate junctions, membrane rafts and membrane microdomains. Biological process (GO-BP) analysis showed enrichment in multiple oxidative stress-related cellular pathways, including response to reactive oxygen species, response to UV, cellular response to abiotic stimulus, cellular response to environmental stimulus and reactive oxygen species metabolic process. Furthermore, genes were enriched in the extrinsic apoptotic signaling pathway and tyrosine phosphorylation-related pathways, including peptidyl-tyrosine phosphorylation, peptidyl-tyrosine modification and regulation of peptidyl-tyrosine phosphorylation.

Enrichment analysis was performed for the two MCODE modules derived from negatively correlated miRNAs.

1. Module 1 (score = 9,556, 10 genes) was enriched, in the Hallmark gene sets, for E2F transcription factor targets (E2F_TARGETS) and G2/M checkpoint-related pathways. Reactome analysis revealed enrichment of these target genes in DNA repair pathways, including homology-directed repair and DNA double-strand break repair, as well as TP53-related pathways (regulation of TP53 activity and transcriptional regulation by TP53). GO cellular component (GO-CC) analysis showed enrichment in the cyclin-dependent protein kinase holoenzyme complex, whereas GO biological process (GO-BP) analysis revealed enrichment in multiple pathways related to the G2/M phase of the cell cycle, such as G2/M transition of the mitotic cell cycle, cell cycle G2/M phase transition and regulation of G2/M transition of the mitotic cell cycle.
2. Module 2 (score = 4,286, 8 genes) showed, in the Hallmark analysis, enrichment of a subset of target genes in the complement system pathway. Reactome analysis further indicated enrichment in signaling by interleukins. GO-BP analysis demonstrated enrichment in biological processes related to smooth muscle cell proliferation, including regulation of smooth muscle cell proliferation and smooth muscle cell proliferation.

**Fig. 6:**
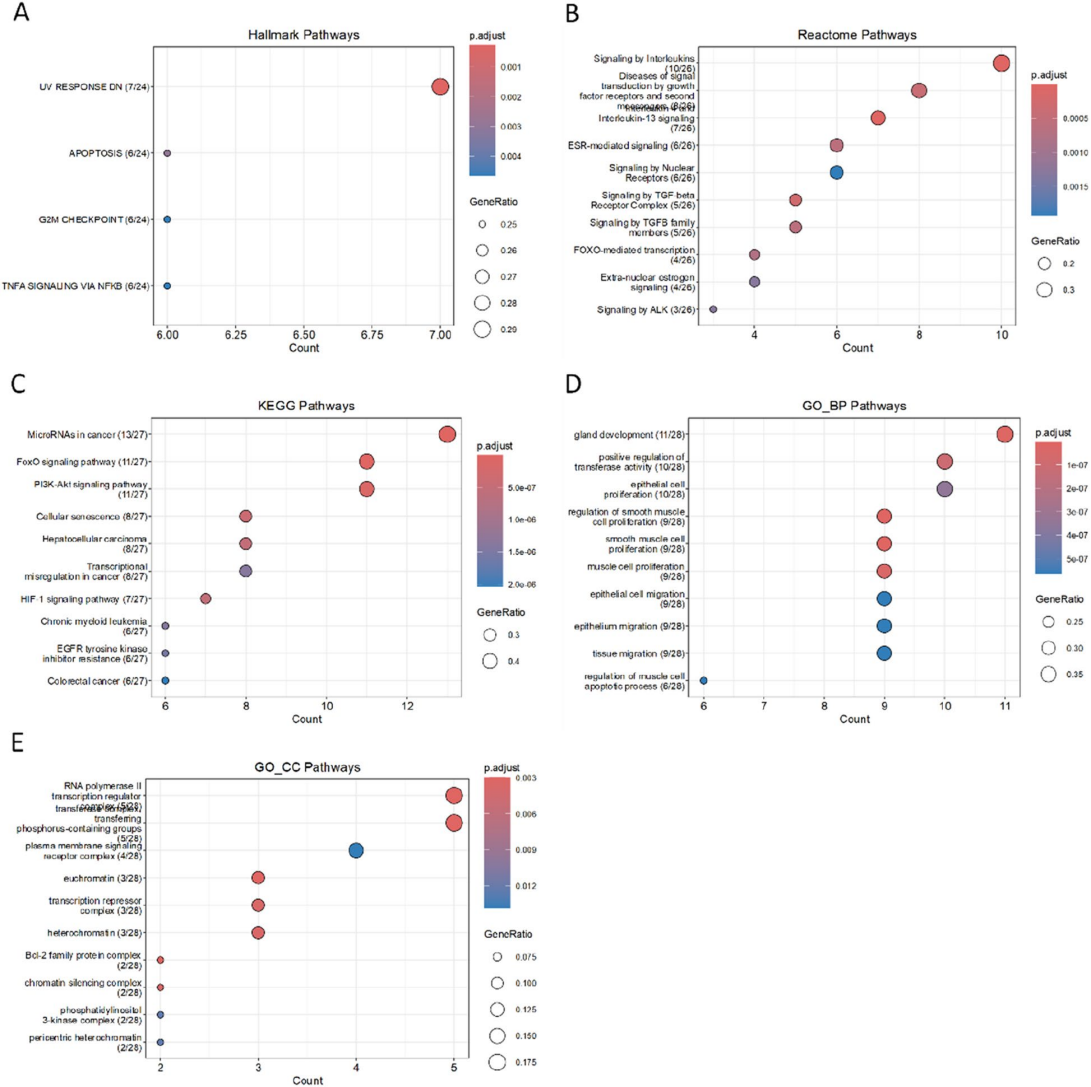
Intersection of target genes of positively and negatively correlated miRNAs

The intersecting target genes of positively and negatively correlated miRNAs were enriched in multiple pathways overlapping with those identified for the above target-gene sets, as shown in (Fig. 6). Hallmark analysis demonstrated enrichment in apoptosis (APOPTOSIS), cell-cycle regulation (G2M_CHECKPOINT), cellular stress (UV_RESPONSE_DN), and TNF-α/NF-κB signaling pathways. Reactome analysis indicated enrichment in interleukin-4 and interleukin-13 signaling, ESR-mediated signaling, and signaling by the TGF-β receptor complex. KEGG analysis similarly revealed enrichment in cellular senescence, as well as FoxO, PI3K-Akt, and HIF-1 signaling pathways. In GO biological process (BP) analysis, these target genes were enriched in terms related to the regulation of smooth muscle cell proliferation, epithelial cell proliferation, and epithelial cell migration. In the GO cellular component (CC) category, they were enriched in the RNA polymerase II transcription regulator complex and the transferase complex involved in transferring phosphorus-containing groups. The enrichment profile of the corresponding MCODE module (score = 15,176; genes = 18) was broadly similar to that observed for all intersecting target genes.

### miRNA differential expression analysis

The 22 included miRNAs were systematically matched to all miRNAs detected in GSE120584, and 8 were not directly matched in the dataset. As these miRNAs were not explicitly annotated for arm origin in the original studies, GSE120584 was further searched for miRNAs from the same family or for potentially corresponding arm-specific miRNAs, which were then included in an exploratory analysis (Table 6). Following this expansion, the final miRNA set included 20 positively correlated miRNAs and 12 negatively correlated miRNAs. In subsequent analyses, miRNAs added after matching-based expansion are indicated by an asterisk (*).

**Table 6:** Literature-derived miRNAs not directly matched in GSE120584 and the corresponding supplemented family or arm-specific miRNAs

Correlation	Included miRNA	Supplemented miRNA
**Positive**	miR-202	miR-202-5p; miR-202-3p
	miR-211	miR-211-5p; miR-211-3p
	miR-222	miR-222-3p; miR-222-5p
	miR-223	miR-223-3p; miR-223-5p
	miR-26b	miR-26b-5p; miR-26b-3p
**Negative**	miR-128	miR-128-3p; miR-128–1-5p; miR-128–2-5p
	miR-138	miR-138-5p; miR-138–2-3p; miR-138–1-3p
	miR-9	miR-9-5p; miR-9-3p

**Fig. 7:**
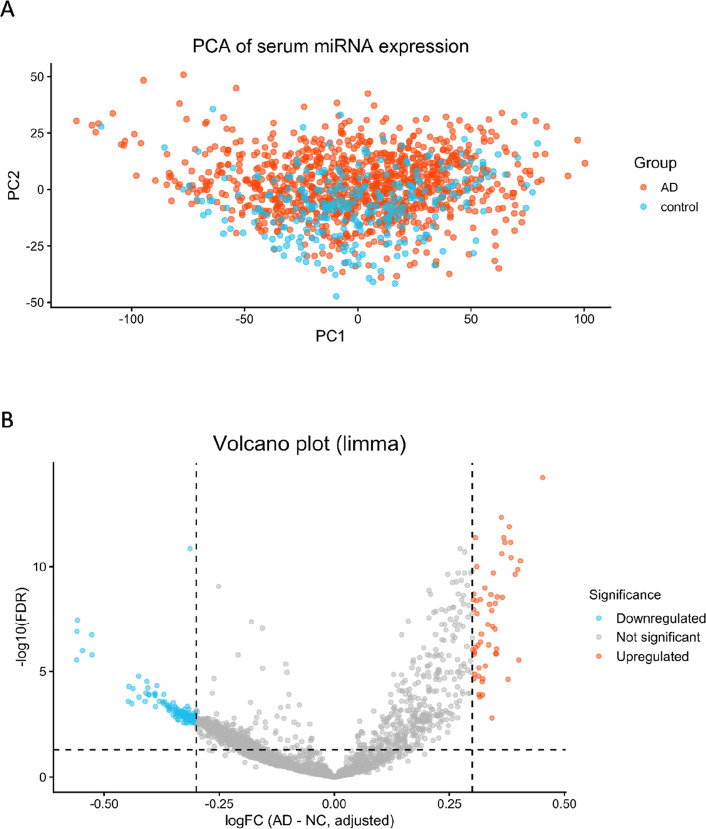
Principal component analysis (PCA) and volcano plots of differential miRNA expression in GSE120584

We next performed differential expression analysis of miRNAs in the GSE120584 dataset. The PCA plot for GSE120584 is shown in (Fig. 7A). Because the PCA plot did not show clear separation between groups, suggesting potential confounding, we included age, sex, and APOE ε4 allele count as covariates in the limma model. The resulting volcano plot is shown in (Fig. 7B).

**Fig. 8:**
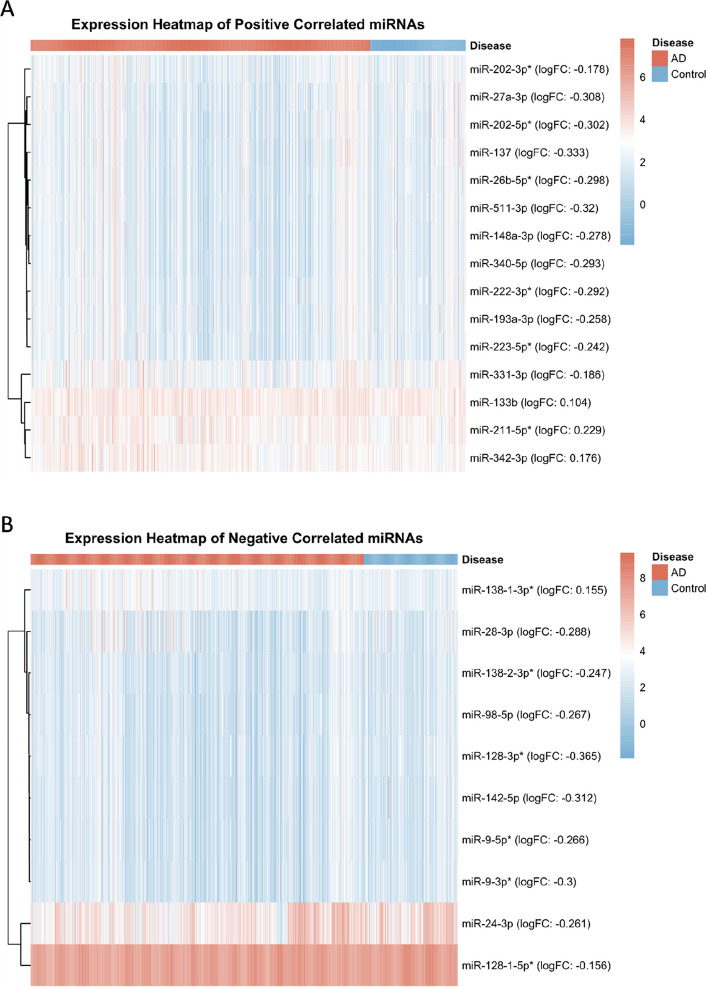
Heat map of expanded positively and negatively correlated miRNAs in the GSE120584 dataset. Note: * indicates miRNAs supplemented after matching

A total of 13 miRNAs were differentially expressed among the included miRNAs, and 12 were identified among the supplementary ones. The heatmap generated from the expanded miRNA dataset is shown in (Fig. 8). In the GSE120584 dataset, most positively correlated miRNAs showed decreased expression in patients with AD, whereas only miR-133b, miR-342-3p, and the expanded miR-211-5p displayed increased expression. Conversely, most negatively correlated miRNAs also showed decreased expression, with only the supplemented miR-138–1-3p being upregulated. Moreover, most expanded miRNAs exhibited low expression levels in both patient groups, except for the positively correlated miR-133b, miR-211-5p*, and miR-342-3p, as well as the negatively correlated miR-24-3p and miR-128–1-5p*. Comparison with the miRTarBase database revealed that among the 14 miRNAs possessing functional MTI target genes, only miR-320a (positively correlated) showed no difference in expression.

### Correlation between miRNA and clinical baseline characteristics

**Fig. 9:**
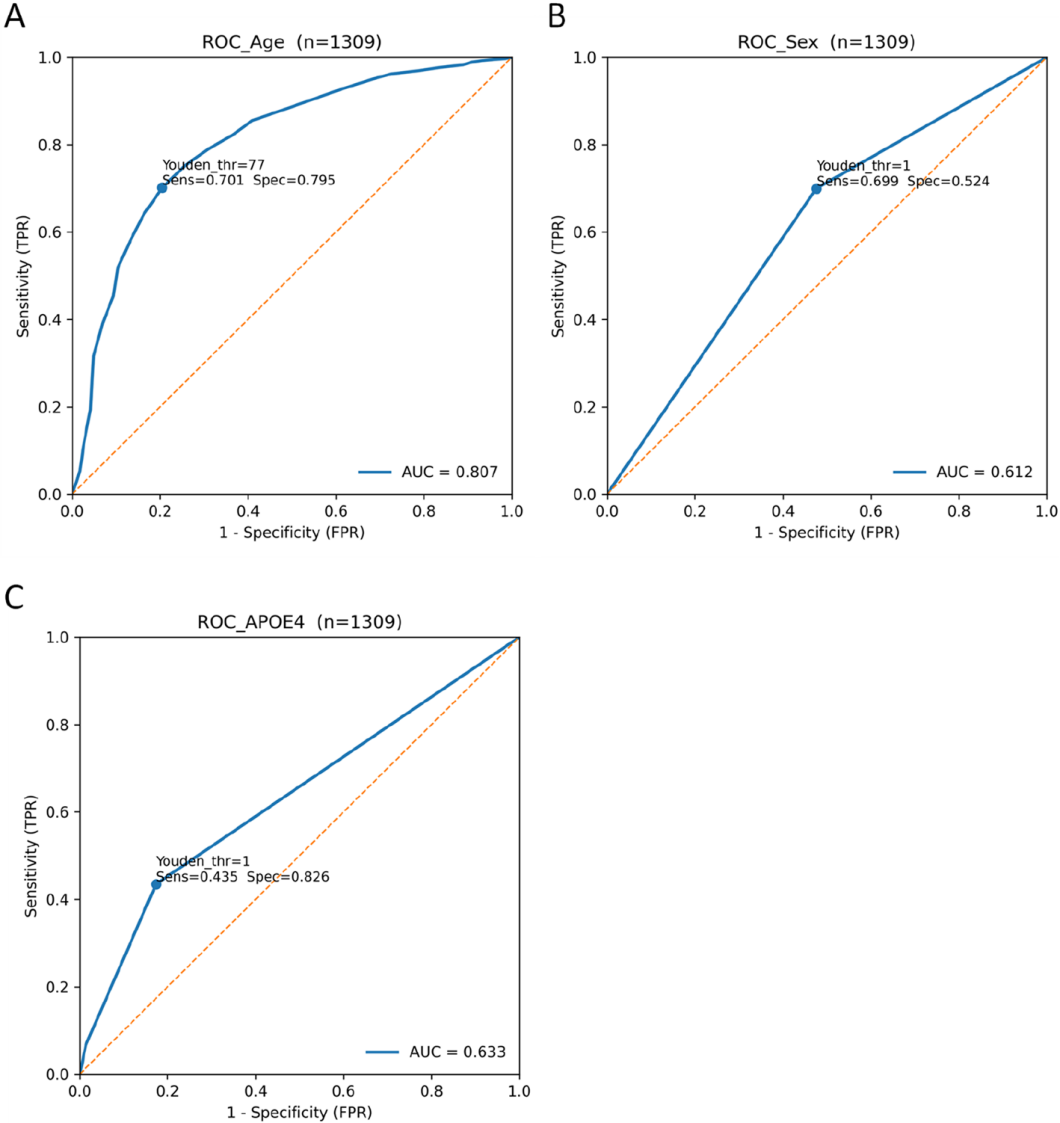
Receiver operating characteristic (ROC) curves for baseline clinical predictors: (**A**) age, (**B**) sex, and (**C**) APOE ε4 allele count

The diagnostic performance of each baseline clinical characteristic was assessed, with the results summarized in (Table 7) and the corresponding ROC curves presented in (Fig. 9). Because the AD and control groups in GSE120584 differed substantially in age, sex, and APOE ε4 distribution, the univariable diagnostic performance described below should be interpreted primarily as reflecting the discriminative capacity of these variables within this cohort.

1. Age showed high discriminative ability for AD (AUC = 0.807, AP = 0.919). The Youden index indicated an optimal cutoff of 77.0 years, at which positive predictions were associated with a higher proportion of AD cases than controls (PPV = 0.924, NPV = 0.429). Five-fold cross-validation suggested a stable contribution of age to diagnostic performance (CV-AUC = 0.809 ± 0.034, CV-AP = 0.920 ± 0.016).
2. Sex showed limited raw discriminative power (AUC = 0.388, AP = 0.747). The optimal threshold based on Youden’s index was 1 (classifying females as the positive group). Nevertheless, five-fold cross-validation yielded relatively robust performance estimates (CV-AUC = 0.612 ± 0.025, CV-AP = 0.821 ± 0.009).
3. APOE ε4 allele count (0/1/2) also exhibited limited discriminative ability (AUC = 0.633, AP = 0.835). The optimal cutoff of 1 (i.e., APOE ε4 allele count ≥ 1 classified as AD) was derived from the Youden index. Five-fold cross-validation indicated that APOE ε4 allele count had stable but modest diagnostic value (CV-AUC = 0.634 ± 0.022, CV-AP = 0.835 ± 0.011).

**Table 7:** Univariate diagnostic performance of baseline clinical predictors (n = 1309)

Univariate		Age	Sex	APOE ε4
**AUC**		0.807	0.612	0.633
**AP**		0.919	0.821	0.835
**Youden’s index**	**Youden’s J statistic**	0.496	0.224	0.261
	**Youden threshold**	77	1 (female)	1
	**Sensitivity**	0.701	0.699	0.435
	**Specificity**	0.795	0.524	0.826
	**Accuracy**	0.722	0.661	0.521
	**PPV**	0.924	0.839	0.899
	**NPV**	0.429	0.330	0.292
**Confusion matrix**	**TP/FP/TN/FN**	716/59/229/305	714/137/151/307	444/50/238/577
**fivefold cross-validation**	**CV-AUC**	0.809 ± 0.034	0.612 ± 0.025	0.634 ± 0.022
	**CV-AP**	0.920 ± 0.016	0.821 ± 0.009	0.835 ± 0.011

*Abbreviations*: *AUC* area under the curve, *AP* average precision, *TP* true positive, *FP* false positive, *TN* true negative, *FN* false negative

Subsequently, we analyzed correlations between the expanded set of included miRNAs (*n* = 32) and clinical baseline characteristics. Partial Spearman correlation analyses were performed, adjusting for the remaining covariates in each model, and Benjamini–Hochberg correction for multiple comparisons was applied (FDR < 0.05 was considered statistically significant). Age showed the broadest pattern of independent associations with miRNA expression, being significantly correlated with 28 miRNAs, whereas sex was independently associated with 12 miRNAs. Overall, the absolute values of the partial correlation coefficients (|r|) for age-related miRNAs were larger than those observed for sex-related effects. The top five miRNAs ranked by |r| for each characteristic are listed in (Table 8). After simultaneously adjusting for age and sex, only hsa-miR-211-5p remained independently associated with APOE ε4 allele count (r = 0.090, q = 0.033). For sex-associated miRNAs, several p-values fell within very similar ranges, and the monotonicity adjustment of the Benjamini–Hochberg procedure resulted in identical q-values for these miRNAs. Complete results of the partial Spearman correlation analyses for all included miRNAs are provided in [Supplementary Material 5].

**Table 8:** Associations of included miRNAs with age and sex identified by partial Spearman correlation analysis

Age-associated miRNA	Partial ρ	q-value	Sex-associated miRNA	Partial ρ	q-value
miR-128–2-5p*	0.328	1.6 × 10^(−322)	miR-128–2-5p*	0.082	0.044
miR-211-3p*	0.194	1.53 × 10^(−11)	miR-320a	−0.074	0.044
miR-24-3p	−0.162	3.16 × 10^(−8)	miR-137	−0.072	0.044
miR-133b	0.152	2.14 × 10^(−7)	miR-128–1-5p*	0.071	0.044
miR-128–1-5p*	−0.133	8.23 × 10^(−6)	miR-9-3p*	−0.070	0.044

^*^indicates the miRNAs supplemented after matching; Partial ρ indicates the partial Spearman correlation coefficient

### Correlation structure and de-duplicated feature selection among included miRNAs

**Fig. 10:**
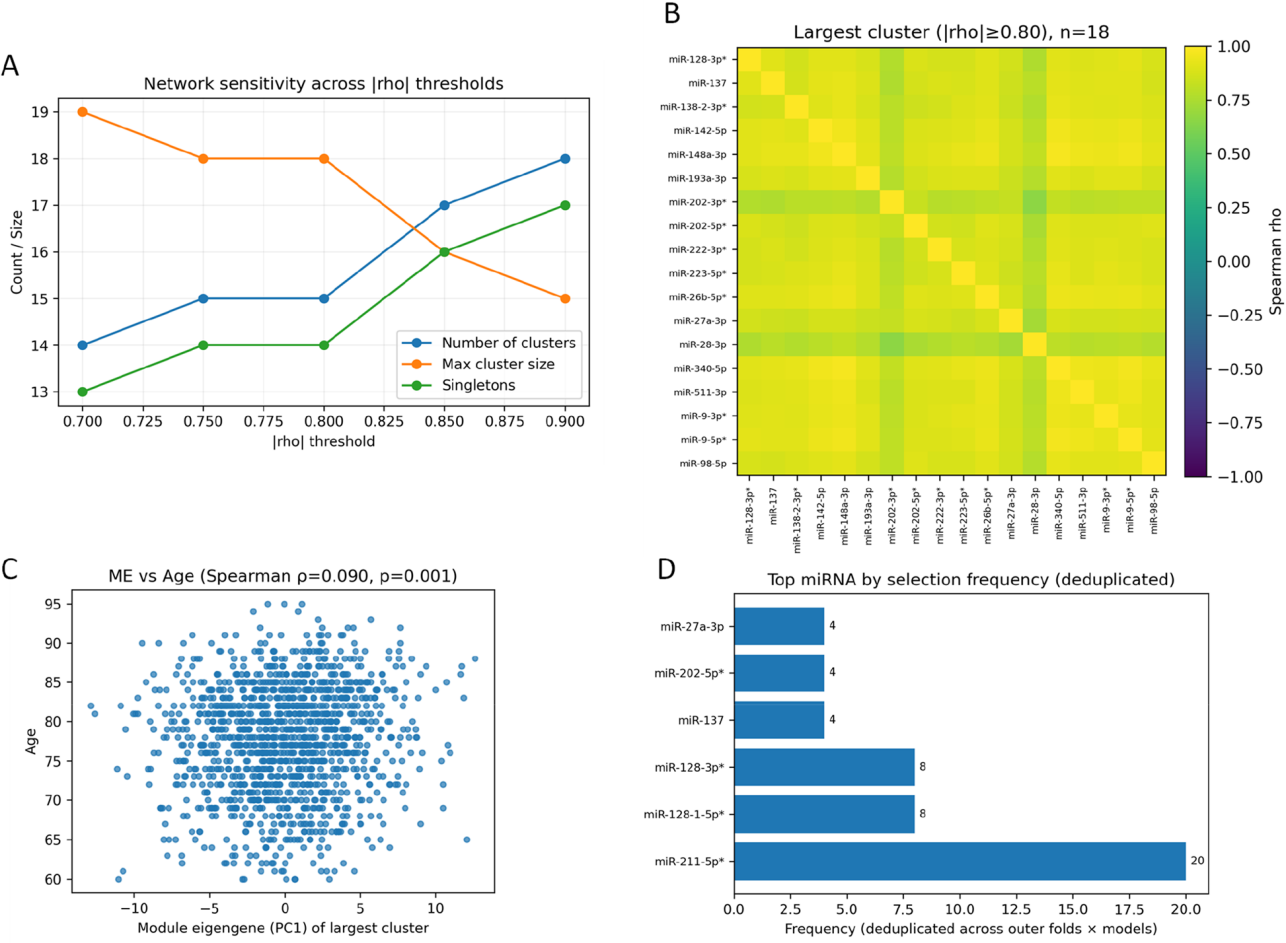
Correlation structure and selection frequency of included miRNAs. (**A**) Sensitivity scan across |ρ| thresholds. (**B**) Pairwise correlation heat map for the largest miRNA cluster (*n* = 17). (**C**) Scatter plot of the first principal component (PC1) of the largest cluster versus age. (**D**) miRNA selection frequencies after de-duplication

We analyzed the correlation structure and redundancy among the included miRNAs. In a |ρ| threshold sensitivity scan, the overall network structure remained stable. When the absolute correlation threshold was relaxed from 0.90 to 0.70, the number of clusters decreased from 18 to 14, the maximum cluster size increased from 15 to 19, and the number of isolated nodes (singletons) decreased from 17 to 13 (Fig. 10A). At |ρ|= 0.80, the largest cluster comprised 18 miRNAs, including miR-128-3p, miR-137, miR-138–2-3p, miR-142-5p, miR-148a-3p, miR-193a-3p, miR-202-3p, miR-202-5p, miR-222-3p, miR-223-5p, miR-26b-5p, miR-27a-3p, miR-28-3p, miR-340-5p, miR-511-3p, miR-9-3p, miR-9-5p and miR-98-5p. The pairwise correlation heatmap within this cluster is shown in (Fig. 10B). Among these miRNAs, miR-28-3p showed the strongest correlation with age (|r|= 0.105627), whereas miR-27a-3p showed the weakest (|r|= 0.053272). The association between the first principal component (PC1) of this cluster and age is presented in (Fig. 10C).

Subsequently, we analyzed feature selection frequencies and constructed a candidate panel. To avoid double counting across configuration dimensions, selection frequencies were aggregated after de-duplication at the outer fold × model level. miR-211-5p was selected in all 20 outer training runs, whereas miR-128–1-5p and miR-128-3p were each selected in 8 of 20 runs. After cluster-based de-duplication (|ρ|= 0.80), the frequency-based panel was ranked as miR-211-5p, miR-128–1-5p and miR-128-3p (Fig. 10D). In the single-miRNA diagnostic value assessment, miR-6761-3p showed the highest five-fold cross-validated AUC in GSE120584 (0.693), whereas miR-128–1-5p showed the highest value among the included miRNAs (0.654) (Table 9). The univariate ranking indicates that hsa-miR-211-5p had a five-fold cross-validated AUC of approximately 0.621 (rank ≈ 73), suggesting that its selection advantage arises mainly from its complementarity with age and its stability across folds, rather than from superior standalone discriminative power.

**Table 9:** Top 10 individual miRNAs ranked by five-fold cross-validated AUC

All miRNA in GSE120584	fivefold CV AUC	Included miRNA	fivefold CV AUC
miR-6761-3p	0.69286331	miR-128–1-5p*	0.654253
miR-1538	0.689357327	miR-211-5p*	0.620747
miR-3184-5p	0.689354337	miR-128–2-5p*	0.606401
miR-6840-3p	0.682148269	miR-342-3p	0.604465
miR-3622a-3p	0.677609488	miR-24-3p	0.59423
miR-6777-3p	0.676044445	miR-138–1-3p*	0.586817
miR-6875-3p	0.674480738	miR-133b	0.577964
miR-6867-3p	0.670517976	miR-26b-3p*	0.565762
miR-1229-3p	0.668177584	miR-211-3p*	0.549039
miR-4713-5p	0.667394451	miR-128-3p*	0.546725

^*^indicates the miRNAs supplemented after matching

### miRNA panel optimization and 1-SE-guided model selection

We used **K** to denote the number of miRNAs included in the highest-diagnostic-value miRNA combination (**M2**), i.e., the panel size. Because we prioritized the inclusion of miRNAs from different clusters, miR-211-5p, miR-128–1-5p, and miR-128-3p were selected from three distinct clusters; therefore, the maximum panel size was 3 (Kmax = 3). These three miRNAs were derived from the expanded candidates related to miR-211 (positively correlated) and miR-128 (negatively correlated). The correspondence between K values and miRNA combinations is shown in Table 10. Among them, K = 2B (miR-211-5p + miR-128-3p) was additionally included for sensitivity analysis. Analyses were performed using age alone and age plus sex as the baseline models.

**Table 10:** Optimal miRNA panels by panel size (K) for AD discrimination

Combination	miRNA
K = 1	miR-211-5p
K = 2A	miR-211-5p + miR-128–1-5p
K = 2B	miR-211-5p + miR-128-3p
K = 3	miR-211-5p + miR-128–1-5p + miR-128-3p

(**1**) Age-only baseline: The baseline area under the curve (AUC) was 0.806, and the one-standard-error (1-SE) threshold was 0.810. At K = 1, adding miR-211-5p improved overall discrimination (ΔAUC = + 0.015), with the largest gain observed in average precision (AP = 0.934). Among K = 2 panels, the combination including miR-128-3p showed marginally better discrimination than that including miR-128–1-5p (ΔAUC = + 0.012). Along the incremental K-AUC path, optimal performance was observed at K = 3 (AUC = 0.823). Applying the 1-SE rule yielded an AUC threshold of 0.810; among panels meeting this criterion, the smallest panel size was K = 1, indicating that the age + miR-211-5p model provided the most parsimonious improvement in performance.

(**2**) Age + sex baseline: The baseline AUC was 0.816, and the one-standard-error (1-SE) threshold was 0.823. We evaluated panel sizes from K = 1 to K_max and applied the 1-SE rule independently. At K = 1, adding miR-211-5p yielded an AUC of approximately 0.810. Along the standard incremental path, the K = 2 panel (miR-211-5p + miR-128–1-5p) achieved an AUC of approximately 0.819, which did not reach the 1-SE threshold. The best performance along the incremental path was observed at K = 3 (AUC ≈ 0.836), and K = 3 was therefore selected as the smallest near-optimal panel under the 1-SE rule. In the sensitivity analysis, the alternative K = 2B panel (miR-211-5p + miR-128-3p) achieved AUC ≈ 0.826 and Brier ≈ 0.171, suggesting stronger complementarity between miR-128-3p and the age + sex baseline; however, the primary analysis followed the standard incremental path and thus retained K = 3.

**Fig. 11:**
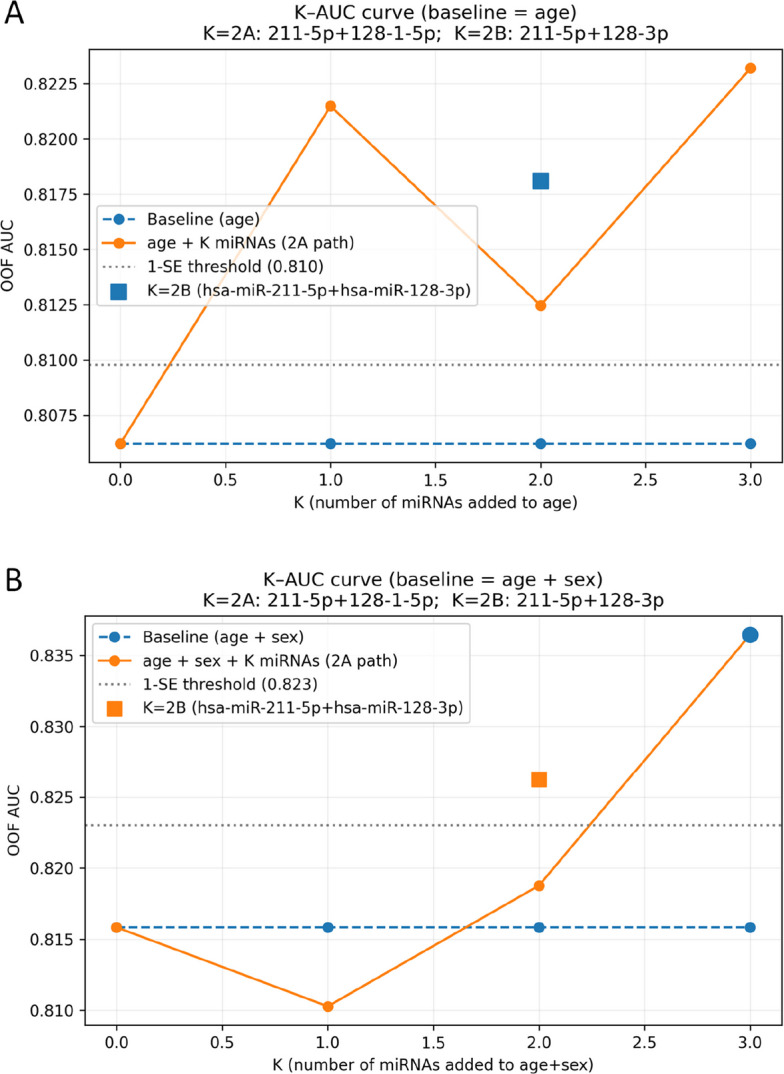
Cross-validated AUC as a function of panel size (K) for candidate miRNA panels under two baseline models: (**A**) age only and (**B**) age + sex

The 1-SE curves are shown in (Fig. 11), and the detailed results are provided in Table 11 and [Supplementary Material 6].

**Table 11:** Performance of incremental miRNA panels under two baseline clinical models

Baseline	miRNA Combination	AUC	95% CI	PR AUC	Brier score
**Age**	K = 0	0.806	0.773–0.839	0.919	0.203
	K = 1	0.821	0.789–0.854	0.934	0.194
	K = 2A	0.812	0.786–0.839	0.920	0.184
	K = 2B	0.818	0.786–0.850	0.928	0.184
	K = 3	0.823	0.797–0.849	0.925	0.179
**Age & Sex**	K = 0	0.816	0.784–0.848	0.930	0.176
	K = 1	0.810	0.778–0.843	0.930	0.186
	K = 2A	0.819	0.791–0.847	0.927	0.179
	K = 2B	0.826	0.797–0.855	0.935	0.171
	K = 3	0.836	0.810–0.863	0.935	0.170

In subsequent analyses, both K = 1 (miR-211-5p) and K = 3 (miR-211-5p + miR-128–1-5p + miR-128-3p) were treated as optimal candidate miRNA panels.

### Development and comprehensive performance evaluation of miRNA-clinical diagnostic models

Elastic net logistic regression (ENet) was used as the primary classifier, with support vector machines (SVMs), random forest (RF), and XGBoost as comparator algorithms in sensitivity analyses. For all included miRNAs (M1), we fitted ENet models based on miRNAs alone and on the optimal miRNA panels (K = 1: miR-211-5p; K = 3: miR-211-5p + miR-128–1-5p + miR-128-3p), and further constructed diagnostic models that combined each miRNA configuration with baseline variables (age or age + sex). ENet performance metrics are summarized in (Table 12), with full analytical details provided in [Supplementary Material 7]. Across all three miRNA-only configurations, diagnostic performance was lower than that of age alone (AUC = 0.807). The best composite models were K3 + age + sex (ENet: AUC = 0.8383, AP = 0.9345, Brier score = 0.1616) and M1 + age + sex (ENet: AUC = 0.8345, AP = 0.9304, Brier score = 0.1630).

**Table 12:** Diagnostic performance of candidate miRNA-based models estimated by elastic net logistic regression

Model		AUC	AP	Brier
**M1**	**M1**	0.7212836	0.883146394	0.212058799
	**M1 + age**	0.826086217	0.925289041	0.168835439
	**M1 + age + sex**	0.834479405	0.930368987	0.162977569
**M2**	**K1**	0.616929889	0.834012495	0.24415593
	**K1 + age**	0.813924937	0.928930441	0.178409571
	**K1 + age + sex**	0.823879095	0.937029694	0.172460215
	**K3**	0.700446866	0.874517656	0.219928987
	**K3 + age**	0.830408641	0.929628191	0.165884968
	**K3 + age + sex**	0.838305311	0.934475832	0.161643067

M1 denotes the model including all included miRNAs, and M2 denotes the model including the selected combination of miRNAs with the highest diagnostic value (K1 = miR-211-5p; K3 = miR-211-5p + miR-128–1-5p + miR-128-3p)

**Fig. 12:**
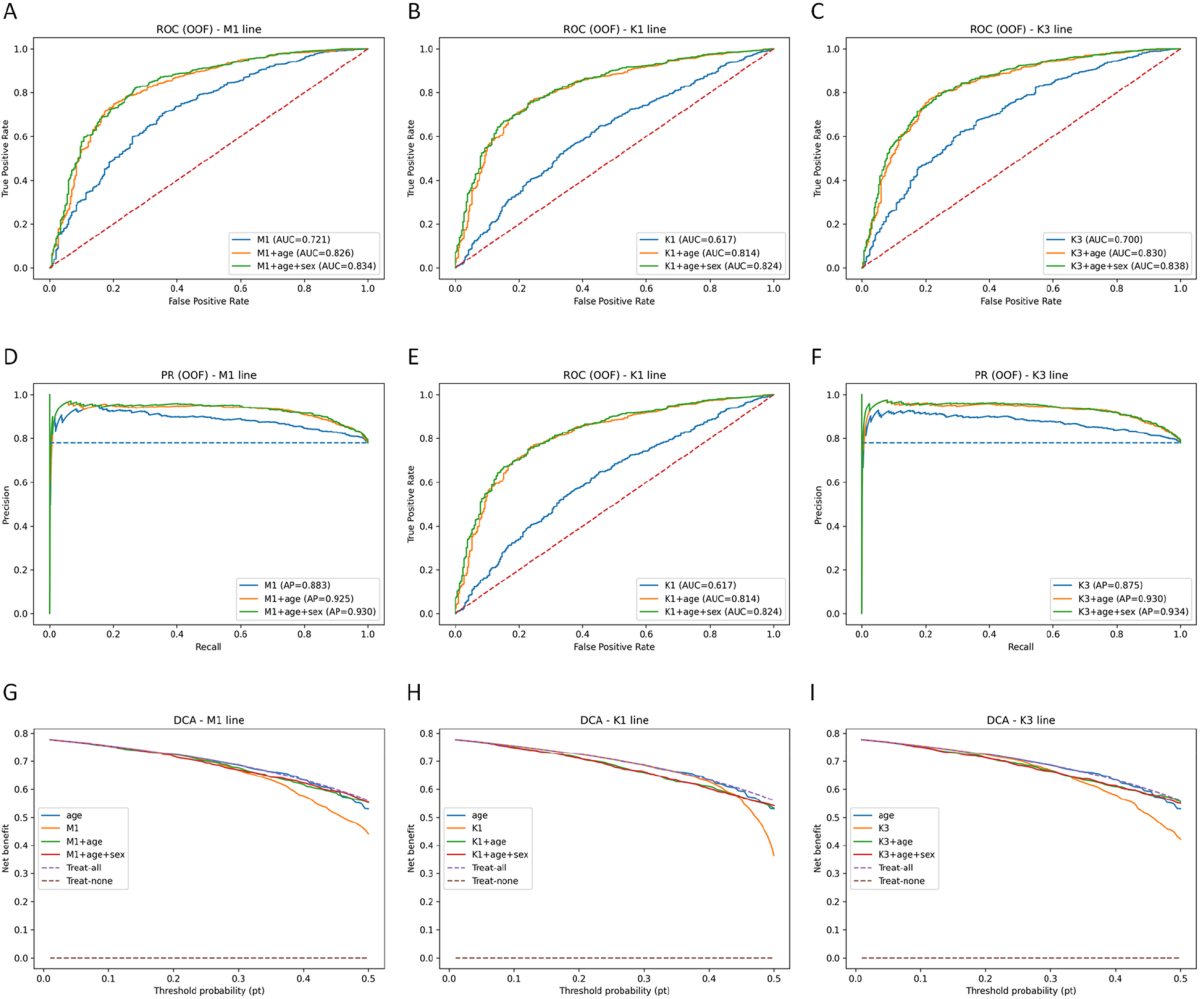
Out-of-fold receiver operating characteristic (ROC), precision–recall (PR), and decision curve analysis (DCA) results for candidate diagnostic models. (**A**-**C**) ROC curves for the M1, K1, and K3 models, respectively. (**D**-**F**) PR curves for the M1, K1, and K3 models, respectively. (**G**-**I**) DCA curves for the M1, K1, and K3 models, respectively

ROC curves derived from out-of-fold probabilities indicated that the main improvement in model performance came from the addition of age (ΔAUC: + 0.197 for K1, + 0.130 for K3, and + 0.105 for M1), with sex providing a further modest yet consistent increase (approximately + 0.008–0.010). Against this background, the miRNA panels offered limited incremental discriminative value, among which **K3 + age + sex** performed best, followed by **M1 + age + sex**, while **K1 + age + sex** showed slightly lower performance. These results suggest that the miRNAs identified in this study are better regarded as adjunctive markers that complement baseline clinical variables, rather than as stand-alone replacements. The ROC curves are presented in (Fig. 12, A-C).

Precision-recall (PR) curves based on out-of-fold predicted probabilities showed that adding the clinical baseline variables age and sex substantially improved precision across almost the entire recall range compared with miRNA-only models. For K1, AP increased from 0.834 to 0.929 after adding age (K1 + age, ΔAP = + 0.095), and further to 0.937 after additionally including sex (ΔAP = + 0.008). K3 and M1 displayed similar patterns (K3: 0.875 → 0.930 → 0.934; M1: 0.883 → 0.925 → 0.930). In the low-to-moderate recall region (≈0.1–0.6), the composite-model curves formed an upper envelope, indicating that higher positive predictive values could be achieved at comparable sensitivity levels, thereby reducing false positives. Among the three composite models, K3 + age (+ sex) provided the most robust performance, although its absolute gain over clinical baseline models was limited. The PR curves are shown in (Fig. 12, D-F).

**Fig. 13:**
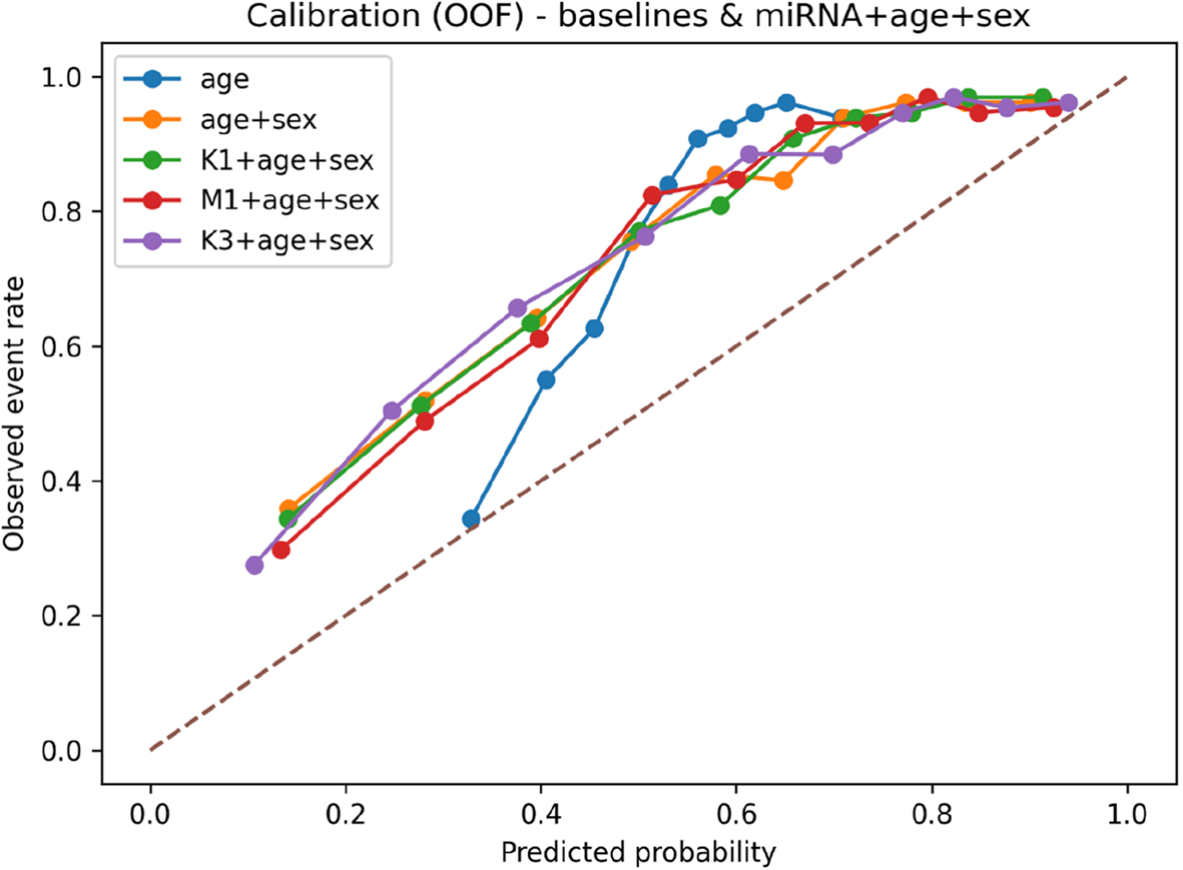
Out-of-fold calibration plots for baseline and composite miRNA-clinical models

The calibration assessment based on out-of-fold predicted probabilities showed that the calibration curves increased monotonically with the predicted probability. However, in the moderate probability range (approximately 0.3–0.6), the curves generally lay above the 45° line, indicating an overall underestimation of risk. Among all models, the age-only baseline exhibited the greatest deviation (expected calibration error [ECE] = 0.246, Brier score = 0.203). Calibration improved after incorporating sex (age + sex: ECE = 0.205, Brier score = 0.176). Further integration of miRNA data shifted the curves closer to the ideal line, with K3 + age + sex showing the best calibration (ECE = 0.185, Brier score = 0.162) and M1 + age + sex the second best (ECE = 0.190, Brier score = 0.163). Taken together, these findings suggest that adding miRNA data to the clinical baseline primarily improves the calibration of predicted probabilities, as shown in (Fig. 13).

In DCA, we compared the net benefit curves of Treat-none, Treat-all, and the candidate models across threshold probabilities (pt) from 0.01 to 0.50. Overall, models based solely on miRNAs (M1, K1, K3) yielded the lowest net benefit across this entire range. Adding age (+ age) produced a marked improvement, and further including sex (+ sex) provided small but consistent gains at low-to-intermediate thresholds (approximately pt 0.05–0.25). The net benefit curve for K3 + age (+ sex) formed a clear upper envelope between pt = 0.10 and 0.35, outperforming the age-only model; M1 + age (+ sex) showed a slightly smaller advantage, whereas K1 + age (+ sex) largely overlapped with the age-only model and did not consistently exceed it. At higher thresholds (pt ≥ 0.35), differences between the age model and the composite models diminished.

Because event prevalence was high in this cohort, the Treat-all curve appeared elevated; in lower-prevalence, real-world screening settings, this curve would be expected to shift downward overall, although the relative ranking of the models would likely remain unchanged. These findings are consistent with the ROC and precision-recall (PR) analyses: age accounted for most of the discriminative gain, whereas miRNAs and sex conferred additional but modest incremental net benefit. Among the miRNA-based panels, the K3 combination provided the most stable improvement in net benefit. The DCA plot is shown in (Fig. 12, G-I).

To facilitate comparison at clinically relevant operating points, we derived a model-specific classification threshold (*Threshold*) from the out-of-fold (OOF) predicted probabilities and computed sensitivity, specificity, and predictive values at that cutoff. For the high-specificity operating point, the cutoff was selected to satisfy the constraint of specificity ≥ 0.90, and the attained sensitivity and PPV at this cutoff are reported in (Table 13). Likewise, for the high-sensitivity operating point, the cutoff was chosen to meet sensitivity ≥ 0.90, and the attained specificity and PPV are summarized in (Table 14).

**Table 13:** Performance of diagnostic models at the high-specificity operating point (specificity ≥ 0.90)

miRNA model		Threshold	Sensitivity	Specificity	PPV	NPV
**M1**	**M1**	0.647759	0.308521	0.902778	0.918367	0.269151
	**M1 + age**	0.661394	0.526934	0.902778	0.95053	0.349933
	**M1 + age & sex**	0.669552	0.553379	0.902778	0.952782	0.363128
**M2**	**K1**	0.547738	0.165524	0.902778	0.857868	0.233813
	**K1 + age**	0.646838	0.53477	0.902778	0.95122	0.353741
	**K1 + age & sex**	0.657476	0.550441	0.902778	0.952542	0.361613
	**K3**	0.652111	0.260529	0.902778	0.904762	0.256158
	**K3 + age**	0.696536	0.520078	0.902778	0.949911	0.346667
	**K3 + age & sex**	0.687512	0.563173	0.902778	0.953566	0.368272

*Abbreviations*: PPV, positive predictive value; NPV, negative predictive value

**Table 14:** Performance of diagnostic models at the high-sensitivity operating point (sensitivity ≥ 0.90)

miRNA model		Threshold	Sensitivity	Specificity	PPV	NPV
**M1**	**M1**	0.367697	0.900098	0.34375	0.829422	0.492537
	**M1 + age**	0.346371	0.902057	0.510417	0.867232	0.595142
	**M1 + age & sex**	0.340497	0.902057	0.552083	0.877143	0.6139
**M2**	**K1**	0.451235	0.910872	0.173611	0.796233	0.35461
	**K1 + age**	0.32546	0.901077	0.458333	0.855019	0.566524
	**K1 + age & sex**	0.315025	0.901077	0.506944	0.86629	0.591093
	**K3**	0.365407	0.900098	0.291667	0.818344	0.451613
	**K3 + age**	0.319876	0.900098	0.524306	0.870265	0.596838
	**K3 + age & sex**	0.310408	0.901077	0.555556	0.877863	0.613027

Threshold was used solely to dichotomize predicted probabilities, with a participant classified as AD when the predicted probability was greater than or equal to the model-specific threshold. Because the optimal cutoff varied across models, comparisons should be made under the same operating-point constraint using the attained performance metrics.

At a specificity of at least 0.90, the K3 + age + sex model achieved the highest sensitivity (sensitivity = 0.563) and the highest positive predictive value (PPV = 0.9536) (see (Table 13)). At high-sensitivity operating points, the same model also provided the highest specificity (specificity = 0.556) and a relatively high PPV (PPV = 0.8779) (see (Table 14)).

## Discussion

### Background on peripheral blood miRNAs as biomarkers for AD

Because currently available treatments cannot reverse the neurodegenerative changes that accumulate before AD is clinically diagnosed, early detection has become a major research priority. The NIA-AA research framework further emphasized that AD should be characterized on the basis of its underlying pathological processes rather than clinical symptoms alone, thereby aligning disease characterization across the preclinical, mild cognitive impairment, and dementia stages [9]. This biomarker-based framework centers on amyloid-β deposition (A), pathologic tau (T), and neurodegeneration (N). This framework classifies biomarkers into the three core pathological axes and incorporates imaging modalities such as amyloid PET (for A), tau PET (for T), brain MRI and fluorodeoxyglucose positron emission tomography (FDG-PET) (for N), as well as cerebrospinal fluid (CSF) biomarkers including Aβ42 or the Aβ42/Aβ40 ratio (for A) and phosphorylated tau (for T).

However, the high cost of neuroimaging and the invasive nature of CSF sampling limit their utility for identifying preclinical AD before overt cognitive symptoms emerge. Consequently, increasing effort has been devoted to developing peripheral blood-based biomarkers.

miRNAs are small non-coding RNAs, typically 19–25 nucleotides in length, that mediate post-transcriptional silencing of target genes [78]. Current evidence indicates that circulating miRNAs in serum and plasma remain relatively stable under common pre-analytical conditions and can be measured reproducibly even after room-temperature storage and repeated freeze–thaw cycles [79–82]. This stability is largely attributable to their encapsulation within extracellular vesicles, including exosomes and ectosomes [83], and to their association with carrier complexes such as Argonaute 2 (AGO2) [84], nucleophosmin 1 (NPM1) [85], and high-density lipoprotein (HDL) [86]. Together, these protective carriers shield circulating miRNAs from nuclease degradation and other pre-analytical influences [86–90].

Accumulating evidence therefore supports circulating miRNAs as noninvasive biomarkers with diagnostic and stratification value across a wide range of systemic diseases, including malignancies [91], cardiovascular diseases [92], neurodegenerative disorders [93], metabolic diseases [94], and infectious diseases [95], and also highlights their potential utility for early detection and prognostic assessment [96].

miRNAs are involved in core pathological processes of AD [97], including the regulation of Aβ production and clearance, tau hyperphosphorylation, neuroinflammation, neuronal oxidative stress, mitochondrial dysfunction, and synaptopathy. Animal and cellular studies have demonstrated the therapeutic potential of miRNAs as targets in AD. For instance, exosome-loaded miR-29b reduced PSEN1 expression and thereby inhibited β-amyloid oligomer formation [98], while targeting MAPT mRNA with miR-166 and miR-724 reduced total tau protein levels by approximately 30% in the central prefrontal cortex of model mice [99]. Intranasal administration of a miR-206-3p antagomir restored synaptic plasticity by upregulating BDNF expression [100]. However, issues such as poor transmembrane permeability [101], on- and off-target side effects [102, 103], complex multi-target interactionscomplex multi-target interactions [104][189], and narrow therapeutic windows [105, 106] mean that miRNA-based therapies for AD have not yet entered clinical trials [107].

Current clinical research therefore focuses on analyzing miRNA expression differences in patients with AD [108] and on evaluating the diagnostic value of specific miRNAs for AD [97]. AD-associated miRNA expression profiles have been identified in the superior temporal gyrus and entorhinal cortex [109], and differential expression of specific miRNAs has been detected in the brains, blood, and cerebrospinal fluid of patients with AD across multiple studies [110]. Current research also indicates significant associations between plasma miRNAs and central A/T/N biomarkers in AD, and some plasma miRNA signatures have been reported to improve the discrimination of A/T/N positivity [111]. However, the mechanisms by which circulating miRNAs cross the blood–brain barrier and their definitive links to central neuropathology in AD remain unclear.

### Main findings of the present study and overall characteristics of the candidate miRNAs

In this study, we systematically screened published case–control studies to identify serum miRNAs that were both differentially expressed in AD and associated with cognitive performance. We then re-evaluated these candidates in GSE120584 and assessed their diagnostic relevance. In total, 22 miRNAs from 23 studies were included and categorized, according to their associations with MMSE scores, as either positively or negatively correlated. Only 14 of these miRNAs had experimentally validated miRNA-target interactions (MTIs) in the miRTarBase database. Enrichment analysis showed that target genes of positively correlated miRNAs were enriched in pathways related to PI3K/AKT/mTOR, Wnt/β-catenin, and interleukin signaling, whereas targets of negatively correlated miRNAs were enriched in cell cycle-related pathways involving E2F transcription factors. The enrichment profile of overlapping target genes largely mirrored that observed for target genes of positively and negatively correlated miRNAs.

For the 8 miRNAs that could not be matched to the GSE120584 dataset, we expanded the panel by including 18 miRNAs from the same families that were present in this dataset. Except for miR-320a, all included positively and negatively correlated miRNAs exhibited differential expression. Among the expanded miRNAs, only miR-223 (miR-223-3p*, miR-223-5p*) showed no differential expression. The expanded positively correlated miRNAs predominantly showed decreased expression, whereas the negatively correlated miRNAs were more frequently downregulated. By reviewing relevant animal and cellular studies, we summarized the roles of these differentially expressed miRNAs in AD pathogenesis, validated through in vivo and in vitro experiments, as shown in (Tables 15 and 16). Among these differentially expressed miRNAs, direct evidence for the involvement of miR-223-5p* (positively correlated), miR-138–1-3p* and miR-138–2-3p* (negatively correlated), or miR-138-3p in AD-related pathological changes is currently lacking. miR-28-3p (negatively correlated) has been reported to show significantly elevated expression levels in APP/PS1 mice [150], in mild cognitive impairment, and in AD patient serum [58], but its role in the AD disease process requires further investigation.

**Table 15:** Mechanistic roles of differentially expressed, positively correlated miRNAs in AD pathology

miRNA	Target/Pathways		Experimental Validation	Model System	Reference
miR-202-3p*	**functional target**	β-catenin↓ & Gli1↓	RT-qPCR, Western blot, immunofluorescence double staining, BrdU incorporation	IL-1β-treated Primary rat oligodendrocyte precursor cells (OPCs)	Li Y et al., 2020 [112]
	**pathway**	miR-202-3p↑ ⇒ β-catenin↓ ⇒ Gli1↓ ⇒ OPC proliferation↑/differentiation↑			
**miR-27a-3p**	**direct target**	GSK3β↓	Luciferase reporter assay, qPCR, Western blot	Human cerebral microvascular endothelial cell line hCMEC/D3	Harati R et al., 2022 [113]
	**pathway**	miR-27a-3p↑ ⇒ GSK3β↓ ⇒ β-catenin↑ ⇒ claudin-5↑/occludin↑ ⇒ TEER↑ permeability↓	TEER measurement, FITC-dextran permeability assay, Western blot, qPCR		
	**functional target**	lncRNA NEAT1 could bind to miR-27a-3p	Dual luciferase reporter assay + RNA pull-down experiments	Aβ₁₋₄₀-induced human neuroblastoma SH-SY5Y cells	Dong LX et al., 2021 [114]
	**protein levels**	miR-27a-3p↑⇒ BACE1, APP, Aβ, Tau, p-Tau↓	Western blot + qPCR + flow cytometry	Rat hippocampal Aβ₁₋₄₀ injection	
	**indirect target**	miR-27a-3p↑consistent with CYP46A1↑	qPCR	Primary cultured astrocytes from C57BL/6J mice treated with Aβ₁₋₄₂	Jaberian Asl B et al., 2025 [115]
	**pathway**	miR-27a-3p↑⇒ CYP46A1-mediated cholesterol efflux (brain cholesterol clearance)	qPCR	Neonatal C57BL/6J mouse primary astrocytes	
miR-202-5p*	**direct target**	APP↓	Dual-luciferase reporter assay + Western blot	Rat pheochromocytoma PC12 cells	Dong LH et al., 2021 [64]
	**pathway**	miR-202-5p↑⇒APP↓ → Aβ↓ → apoptosis↓	TUNEL apoptosis assay + MTT cell viability assay	Rat pheochromocytoma PC12 cells treated with Aβ	
	**direct target**	eIF4E↓	Dual-luciferase reporter assay + RIP assay	Mouse neuroblastoma N2a cells subjected to OGD/R	Li B et al.,2019 [116]
	**pathway**	miR-202-5p↑⇒p-Akt, p-GSK-3β, p–c-Raf, p-BAD↑ (Akt/GSK-3β signaling pathway)	Western blot	N2a cells + SD rat middle cerebral artery occlusion model	
**miR-137-3p** **(miR-137)**	**functional target**	TNFAIP1↓	qPCR + RIP assay	HEK293T cells	Li Y et al.,2022 [117]
	**pathway**	miR-137↑⇒TNFAIP1↓⇒ apoptosis↓, autophagy↓↓, amyloidosis↓	TUNEL apoptosis assay + autophagy fluorescence assay + AlphaLISA amyloidosis assay	Aβ₂₅₋₃₅-induced human neuroblastoma SH-SY5Y cells	
	**direct target**	TNFAIP1↓	Dual-luciferase reporter assay + qPCR/Western blot expression validation	mouse neuroblastoma N2a cells + primary mouse cortical neurons	He D et al.,2017 [118]
	**pathway**	miR-137↑⇒TNFAIP1↓⇒NF-κB↓ ⇒ apoptosis↓, Caspase-3 ↓(NF-κB signaling pathway)	MTT viability assay + flow cytometry apoptosis assay + Caspase-3 activity assay + ELISA + Western blot	primary neurons and N2a cell model only	
	**direct target**	PTN↓	Dual-luciferase reporter assay + qRT-PCR + Western blot	Human neuroblastoma SK-N-SH cells	Yang L et al.,2020 [119]
	**pathway**	miR-137↑⇒PTN↓⇒ phosphorylation of PTN/PTPRZ pathway proteins↓⇒apoptosis↓	Western blot + flow cytometry	SK-N-SH cells	
	**direct target**	KREMEN1↓	Dual-luciferase reporter assay + qRT-PCR + Western blot	HEK293 cells + SH-SY5Y cells and human primary neurons	Wang H et al.,2019 [120]
	**pathway**	miR-137↑⇒KREMEN1↓⇒ Aβ-induced apoptosis↓, cell viability and MMP↑	MTT assay + Flow cytometry + JC-1 MMP assay + Western blot	SH-SY5Y cells and human primary neurons	
miR-26b-5p*	**direct target**	MME↓	Dual-luciferase reporter assay + RT-qPCR + Western blot	293 T cells + PC12 cells	Chen L et al.,2025 [121]
	**pathway**	miR-26b-5p↓⇒MME↓⇒ Aβ degradation↓⇒apoptosis and oxidative stress↑	MTT assay + LDH release + Flow cytometry + MDA/SOD/CAT assays + Western blot	rat pheochromocytoma PC12 cells	
	**functional target**	CYP27B1↓	qRT-PCR	Primary rat cortical neurons	Dursun E et al.,2019 [122]
	**pathway**	miR-26b-5p↑⇒CYP27B1↓⇒active vitamin D synthesis↓⇒ affects neuronal differentiation and survival	qRT-PCR		
**miR-511-3p**	**pathway**	miR-511-3p↑⇒ inflammatory cytokines(IL-1β, IL-6, TNF-α) ↓⇒Aβ-induced cell damage and inflammation↓	ELISA + MTT	Aβ₁₋₄₀ treated SH-SY5Y cells	Wang T et al.,2023 [73]
**miR-148a-3p**	**direct target**	p35 (encoded by CDK5R1)	Dual-luciferase reporter + qRT-PCR + Western blot	HEK293 cells	Zeng L et al.,2021 [123]
		PTEN			
	**pathway**	miR-148a-3p↑⇒p35↓⇒ CDK5 activity↓⇒ p-tau↓	Western blot + Co-IP for p35-CDK5 binding	SH-SY5Y with Swedish APP	
		miR-148a-3p↑⇒ PTEN↓⇒ Akt-p↑⇒ CREB-p↑⇒ miR-148a-3p(PTEN/Akt/CREB feed-forward loop) ⇒tau phosphorylation↓	Western blot + ChIP + promoter-luciferase reporter		
**miR-340-5p**	**direct target**	POT1	Dual-luciferase reporter + qRT-PCR + Western blot	HEK293 cells	Li X et al.,2021 [124]
	**pathway**	miR-340-5p↑ ⇒ POT1↓ ⇒ telomerase recruitment↑ ⇒ telomere length↑ ⇒ cellular senescence↓ ⇒ AD symptoms alleviated	TRF + TRAP-ELISA + SA-β-Gal + behavioral tests	HT22 mouse hippocampal neuronal cells induced with Aβ42 oligomers	
		miR-340-5p↑⇒ POT1↓⇒ telomerase activity↑⇒ telomere length↑⇒ Aβ1–42↓⇒ cognitive improvement	Morris water maze + passive avoidance + TRF + TRAP-ELISA + Aβ1–42 IHC	D-galactose- induced ICR mice	
miR-222-3p*	**functional target**	FERMT2↓	Western blot + APP metabolism functional experimen	HEK293 cells, HeLa cells	Eysert F et al.,2021 [125]
	**pathway**	miR-222-3p↑⇒FERMT2↓⇒APP cell surface level↑⇒Aβ secretion↑, sAPPα secretion↑	Western blot + Cell-surface biotinylation assay + Alpha-LISA assay	HEK293-APP695WT cells; Primary neurons: Postnatal day 0 (P0) rat hippocampal neurons	
**miR-193a-3p**	**direct target**	PTEN↓	Dual-luciferase reporter + qRT-PCR + Western blot	PC12 rat pheochromocytoma cells/SH-SY5Y human neuroblastoma cells + Aβ_25–35_ induction	Cao F et al.,2019 [57]
	**pathway**	miR-193a-3p↑⇒PTEN↓⇒ PI3K-AKT activity↑⇒cell viability↑, apoptosis↓, alleviates Aβ toxicity	MTT + Flow cytometry		
miR-223-5p*	-				
**miR-331-3p**	**direct target**	Sqstm1	Dual-luciferase reporter assay + qRT-PCR + Western blot	293 T cells + SH-SY5Y cells + APPswe/PS1dE9 transgenic AD mice	Chen ML et al.,2021 [126]
	**pathway**	miR-331-3p↑⇒Sqstm1↓⇒ autophagy activity↓⇒Aβ clearance↓	Western blot + Immunofluorescence + behavioral tests	APPswe/PS1dE9 transgenic AD mice	
	**direct target**	VHL	Dual-luciferase reporter assay + qRT-PCR + Western blot	Aβ₁₋₄₀-induced SH-SY5Y human neuroblastoma cells	Liu Q et al.,2020 [65]
	**pathway**	miR-331-3p↑⇒VHL↓⇒cell viability↑, inflammation↓	MTT + ELISA + qRT-PCR		
**miR-133b**	**direct target**	EGFR	Dual-luciferase reporter assay + RT-qPCR + Western blot	Aβ₁₋₄₀-induced SH-SY5Y human neuroblastoma cells	Yang Q et al.,2019 [56]
	**pathway**	miR-133b↑⇒EGFR↓⇒cell viability↑, apoptosis↓	MTT + Flow cytometry + RT-qPCR	Aβ₁₋₄₀-induced SH-SY5Y human neuroblastoma cells	
miR-211-5p*	**direct target**	Ngn2	Dual-luciferase reporter assay + qRT-PCR + Western blot	Aβ₁₋₄₂-inducedPC12 rat pheochromocytoma cells	Liu XH et al.,2021 [127]
	**pathway**	miR-211↑⇒Ngn2↓⇒PI3K/AKT phosphorylation↓⇒ proliferation↓, apoptosis↑	Western blot + MTT + Colony formation + TUNEL & Flow cytometry		
	**direct target**	NUAK1	qRT-PCR + Western blot + Dual-luciferase assay	Neuro2A mouse neuroblastoma cells + Primary mouse cortical neurons (E18.5) + APP/PS1 double transgenic AD mouse model	Fan C et al.,2016 [128]
	**pathway**	miR-211-5p↑⇒NUAK1↓⇒ neurite length↓, branching↓	Immunofluorescence + ImageJ quantification + MTT	Aβ₁₋₄₂-treated primary cortical neuron model	
	**direct target**	NEP	qRT-PCR + Western blot + dual-luciferase reporter assay	SH-SY5Y human neuroblastoma cells	Chen H et al.,2024 [129]
	**pathway**	miR-211-5p↑⇒NEP↓⇒ decreased Aβ clearance⇒ enhanced neurotoxicity	Annexin V/PI staining + Transwell migration assay + ELISA	Aβ1–40-treated SH-SY5Y cell model	
	**direct target**	SIRT1	qRT-PCR + Western blot + luciferase reporter assay + immunofluorescence	Rats intracerebroventricular injection of STZ + Neuro-2a: transfected with Aβ1–42	Zhu R et al.,2020 [130]
	**pathway**	miR-211-5p↑⇒SIRT1↓⇒Nrf2↓⇒HO-1↓⇒oxidative stress↑ (MDA/ROS↑, SOD/GSH/GPX↓)	Annexin V/PI staining + Transwell + ELISA		
**miR-342-3p**	**pathway**	miR-342-3p↑⇒JNK/c-Jun activation↑⇒neuronal apoptosis↑⇒Aβ deposition↑⇒ cognitive decline worsens	Western blot + MTT viability, IHC + ELISA (Aβ1–40/42)	3xTg-AD mice + HT22 cells	Fu Y et al.,2019 [131]
	**direct target**	Chi3l1	qRT-PCR + Western blot + Dual-luciferase assay	APPsw-Tg mice + ApoE-/-mice (atherosclerosis model) + HUVEC/iMAEC/VSMC (human/mouse endothelial and smooth muscle cells)	Jung YY et al.,2018 [132]
	**pathway**	miR-342-3p↓⇒Chi3l1↑⇒ VCAM1/ICAM1↑, eNOS↓, NO↓⇒EC inflammation↑;	qPCR + NO assay + THP-1 monocyte adhesion assay + VSMC migration/proliferation assays (BrdU, Wound healing)		
		miR-342-3p↓⇒Chi3l1↑⇒PDGF-BB-induced VSMC migration/proliferation↑⇒ atherosclerotic plaque formation

**Table 16:** Mechanistic roles of differentially expressed, negatively correlated miRNAs in AD pathology

miRNA	Target/Pathways		Experimental Validation	Model System	Reference
**miR-98-5p**	**direct target**	SNX6	qRT-PCR + Western blot + luciferase reporter assay + ELISA + MTT viability + flow cytometry	SK-N-SH cels, SH-SY5Y cells HEK293 cells	Li Q et al., 2016 [133]
	**pathway**	miR-98-5p↑ ⇒ SNX6↓ ⇒ BACE1↑ ⇒ sAPPβ↑, βCTF↑ ⇒ Aβ40/42↑ ⇒ apoptosis↑, viability↓	Western blot + ELISA (Aβ40/42) + MTT (viability) + flow cytometry		
	**direct target**	α7 nAChR↓	qRT-PCR + Western blot + luciferase reporter assay	HEK293T cells, BV-2 cells APP/PS1 transgenic mice, C57BL/6J wild-type (WT) mice	Song C et al.,2021 [134]
	**pathways**	miR-98-5p↑ ⇒ α7 nAChR↓ ⇒ Ca^2^⁺ signaling↓ ⇒ CaM/CaMKII↓ ⇒ synaptic proteins↓ ⇒ cognitive deficits↑	Western blot + immunofluorescence + ROS assay + ELISA,		
		miR-98-5p↑ ⇒ α7 nAChR↓ ⇒ NF-κB↑ ⇒ inflammation↑			
		miR-98-5p↑ ⇒ α7 nAChR↓ ⇒ Nrf2↓ ⇒ HO-1/NQO-1↓ ⇒ antioxidant↓			
	**direct target**	circ_0061183 (circular RNA)	Dual-luciferase reporter assay	293 T human embryonic kidney cells	Zeng HX et al., 2025 [135]
	**pathway**	miR-98-5p↑ ⇒ IL10, BMP2, TGFβR1↓ ⇒ blocks TGF-β signaling ⇒ microglial M2 polarization↓	qRT-PCR + Western blot	HMC3 human microglial cells	
	**direct target**	HEY2↓	Dual-luciferase reporter assay	293 T human embryonic kidney cells	Chen FZ et al., 2018 [136]
	**pathway**	miR-98-5p↑ ⇒ HEY2 ↓ ⇒ Jagged1 ↓ & Notch1 ↓ & Hes1/5 ↓ ⇒ APP ↓ & Bax ↓ & Bcl-2 ↑ ⇒ Aβ production ↓ & oxidative stress ↓ & mitochondrial dysfunction ↓	qRT-PCR + Western blot	Primary hippocampal neurons from AD mice	
	**functional target**	CYP46A1↓	qRT-PCR	Aβ₁₋₄₂ treated primary cultured astrocytes from C57BL/6J mice	Jaberian Asl B et al., 2025 [115]
	**pathway**	Aβ ⇒ miR-98-5p ↓ ⇒ CYP46A1 ↑ ⇒ 24-hydroxycholesterol ↑ ⇒ brain cholesterol efflux ↑			
**miR-142-5p**	**functional target**	PSD-95↓	qRT-PCR + immunocytochemistry	Aβ₄₂ treated human neuroblastoma SH-SY5Y cells	Song J et al., 2017 [137]
	**pathway**	Aβ₄₂ ⇒ miR-142-5p↑ ⇒ (predicted targets AKAP5, DRD1, etc. ↓) ⇒ PSD-95 protein ↓ ⇒ synaptic dysfunction			
	**direct target**	BAI3↓	Dual-luciferase reporter assay	Aβ₁₋₄₂ treated HEK293T human embryonic kidney cells and HT-22 mouse hippocampal neuronal line	Fu CH et al., 2021 [138]
	**pathway**	miR-142-5p ↑ ⇒ BAI3 ↓ ⇒ pCaMKII ↓ & PSD-95 ↓ & p-Synapsin ↓ ⇒ synaptic plasticity impaired ⇒ spatial learning/memory deficit	Western blot + immunofluorescence + Morris water maze	APP/PS1 double-transgenic male mice (6–8 months); Aβ₁₋₄₂ treated primary mouse hippocampal neurons; HT-22 cells	
	**direct target**	PTPN1↓	Dual-luciferase reporter assay	293 T human embryonic kidney cells; Sprague–Dawley rat single lateral ventricle injection of Aβ₁₋₄₂	Liang W et al., 2022 [139]
	**pathway**	miR-142-5p↑ ⇒ PTPN1 ↓ ⇒ p-Akt/Akt ↓ ⇒ Bax ↑ & Bcl-2 ↓ ⇒ neuronal apoptosis ↑ ⇒ learning/memory impairment	Western blot + IHC + ELISA + TUNEL apoptosis assay		
miR-9-5p*	**direct target**	OPTN↓	Dual-luciferase reporter assay	293 T human embryonic kidney cells	Chen ML et al., 2021 [126]
	**pathway**	miR-9-5p↑ ⇒ OPTN↓ ⇒ autophagy activity↓ ⇒ Aβ clearance↓ ⇒ Aβ accumulation↑	Western blot + qRT-PCR + immunofluorescence	SH-SY5Y cells, APPswe/PS1dE9 mice	
	**direct target**	BACE1↓	Dual-luciferase reporter assay	Human neuroblastoma SH-SY5Y cells	Ding Y et al., 2021 [140]
	**pathway**	miR-9-5p↑ ⇒ BACE1↓ ⇒ C99/C83 ratio↓ ⇒ Aβ deposition↓ ⇒ neurotoxicity ↓	qRT-PCR + Western blot + CCK-8 + flow cytometry for apoptosis	R/B/Aβ SH-SY5Y cells; C57BL/6 mice (hippocampal injection of Aβ₁₋₄₂ + BDNF-AS knockdown)	
	**direct target**	GSK-3β	Dual-luciferase reporter assay	Aβ₂₅₋₃₅ treated mouse hippocampal neuronal cell line HT22	Liu J et al., 2020 [141]
	**pathway**	miR-9-5p↑ ⇒ GSK-3β↓ ⇒ stabilized mitochondrial membrane potential, ROS↓, apoptosis↓, Nrf2/Keap1 antioxidant signaling↑	CCK-8 + flow cytometry for apoptosis + JC-1 mitochondrial potential + ROS fluorescent probe + Western blot		
	**direct ligand**	TLR7/TLR8↓	HEK-Blue reporter assay + TNF-ELISA + confocal imaging + RNA-seq + qRT-PCR	human THP-1 macrophages, iPSC-derived human cortical neurons (iNeurons), mouse primary cortical neurons	Kumbol V et al., 2025 [142]
	**pathway**	miR-9-5p↑ ⇒ TLR7/8 activation ⇒ TNF/IL-1α/IL-6 expression↑ ⇒ axonal length↓, excitatory synapse VGLUT1↓, neuronal apoptosis↑	High-content imaging + TUNEL apoptosis assay + Western blot + qRT-PCR	iNeurons (human), human THP-1 macrophages, C57BL/6 and Tlr7 −/− mouse primary neurons	
	**direct target**	UBE4B↓	miRNA-mRNA pull-down + qRT-PCR + RNAi screen + Western blot	Drosophila S2 cells, adult Drosophila eye neurons, human SH-SY5Y neuroblastoma cells	Subramanian M et al., 2021 [143]
	**pathway**	miR-9↑ ⇒ UBE4B↓ ⇒ Tau ubiquitination↓ ⇒ autophagy-mediated Tau degradation↓ ⇒ Tau aggregation↑ ⇒ neurodegeneration↑	Western blot + autophagy inhibitor treatment + co-immunoprecipitation	SH-SY5Y cells, Tau-BiFC mouse model	
	**functional target**	BACE1↓	Western blot + RT-qPCR	Human corneal fibroblasts	Choi SI et al., 2019 [144]
	**pathway**	miR-9-5p↑ ⇒ BACE1↓ ⇒ APP β-cleavage↓ ⇒ β-CTF↓ ⇒ AICD↓ ⇒ Aβ production↓			
miR-9-3p*	**direct target**	Dmd (dystrophin) ↓/SAP97 (Dlg1) ↓	luciferase reporter assay + Western blot	mouse hippocampal CA1 neurons, HEK-293T cells	Sim SE et al., 2016 [145]
	**pathway**	miR-9-3p↓ ⇒ Dmd/SAP97↑ ⇒ disturbed AMPAR trafficking ⇒ LTP impairment ⇒ spatial & trace memory deficits	hippocampal slice whole-cell patch-clamp LTP/LTD + Morris water maze + object-location memory + trace fear conditioning	adult C57BL/6N mice	
**miR-24-3p**	**direct target**	KLF8↓	dual-luciferase reporter assay + qRT-PCR	Aβ_25–35_ treated human neuroblastoma SH-SY5Y cells	Liu L et al., 2021 [66]
	**pathway**	miR-24-3p↑ ⇒ KLF8↓ ⇒ proliferation↓, apoptosis↑ ⇒ exacerbated AD cell injury	CCK-8 cell viability + Annexin V-FITC/PI flow-cytometry apoptosis assay		
**miR-128-3p**	**direct target**	PPARG↓	miRNA-seq + qRT-PCR + TargetScan prediction + Western blot + luciferase reporter	SAMP8 mouse cortical tissue, primary hippocampal neurons (APP/PS1)	Bellver-Sanchis A et al., 2024 [146]
	**pathway**	miR-128-3p↓ ⇒ PPARG↑ ⇒ antioxidant enzymes↑, pro-oxidant enzymes↓ ⇒ OS↓ survival↑	qRT-PCR + Western blot + LDH survival assay		
**miR-128-5p** (miR-128–1-5p*)	**direct target**	STIM2↓	single-cell qPCR + Western blot + luciferase reporter	6-month male APP/PS1 mossy cells, HEK293T reporter cells	Deng M et al., 2021 [147]
	**pathway**	miR-128-5p↑ ⇒ STIM2↓ ⇒ impaired ER Ca^2^⁺ sensing ⇒ ↓ glutamate release probability ⇒ MC → SST synaptic failure ⇒ memory imprecision	dual patch-clamp EPSC/PPR + LST touchscreen (behavior) + LNA blocking oligo	6-month male APP/PS1 mice	
	**direct target**	GSK-3β↓	Luciferase reporter assay + Western blot	293T-Tau cells, SH-SY5Y cells	Li S et al., 2023 [148]
	**pathway**	miR-128↑ ⇒ GSK-3β↓ ⇒ Tau phosphorylation↓	Western blot		
	**direct target**	APPBP2↓	Luciferase reporter assay + RT-qPCR + Western blot	N2a-APPsw cells, SH-SY5Y cells	
	**pathway**	miR-128↑ ⇒ APPBP2↓ ⇒ APP↓ ⇒ Aβ↓	ELISA + Western blot	N2a-APPsw cells	
	**direct target**	mTOR↓	Luciferase reporter assay + Western blot	N2a-APPsw cells, SH-SY5Y cells	
	**pathway**	miR-128↑ ⇒ mTOR↓ ⇒ LC3-II↑ ⇒ autophagy↑ ⇒ Aβ↓	Western blot + TEM + fluorescence microscopy	N2a-APPsw cells, N2a-tfLC3 cells	
	**direct target**	PPAR-γ	Luciferase reporter assay + RT-qPCR + Western blot	Primary mouse cortical neurons, Aβ_1–42_ treated Neuro2a cells	Geng L et al., 2018 [149]
	**pathway**	miR-128↑ ⇒ PPAR-γ↓ ⇒ NF-κB↑ ⇒ Caspase 3↑ ⇒ apoptosis↑	MTT assay + Flow cytometry + Caspase 3 & NF-κB activity assay		
miR-138–1-3p*	**-**				
miR-138–2-3p*	**-**				
**miR-28-3p**	**-**				

### Potential biological roles and pathological significance of differentially expressed miRNAs

Tables 15 and 16 summarize recent publications reporting mechanistic studies on the therapeutic effects of the included miRNAs in AD.

Importantly, the in vivo and in vitro experimental evidence and pathway-related data presented in these two tables primarily support the biological interpretability of the associations observed in this study between the identified miRNAs and AD pathology, but do not permit a direct causal inference regarding the relationship between these miRNAs and AD.

Based on Table 15, most positively correlated miRNAs exert anti-AD pathological effects and neuroprotective functions in experimental models. miRNAs such as miR-202-5p and miR-137 inhibit apoptosis and protect neurons through pathways including PTEN-PI3K/AKT, NF-κB, and EGFR, while miR-511-3p and miR-193a-3p exhibit anti-neuroinflammatory effects. Multiple miRNAs, including miR-27a-3p, miR-26b-5p, and miR-148a-3p, regulate Aβ production and clearance as well as tau phosphorylation. Among these miRNAs, only miR-133b, miR-342-3p, and miR-211-5p showed increased expression. Specifically, miR-133b may exert neuroprotective effects by inhibiting EGFR-related signaling pathways, thereby reducing Aβ-associated neurotoxicity [56]. Conversely, miR-211-5p acts as a “promoting miRNA,” exacerbating Aβ-related neuronal damage through multiple pathways: it targets Ngn2 to inhibit the PI3K/AKT signaling pathway and thereby increase neuronal apoptosis [127]; targets NUAK1 to reduce neuronal axon growth and branching [128]; suppresses NEP to decrease Aβ clearance from neurons [129]; and inhibits the SIRT1/Nrf2/HO-1 antioxidant pathway, thereby exacerbating oxidative stress in neurons [130]. miR-342-3p may exert distinct functions in central versus peripheral vascular systems: small-sample studies have revealed increased hippocampal expression of miR-342-3p in patients with AD, while in vitro and in vivo experiments have demonstrated its pathological role in promoting Aβ deposition and neuronal apoptosis by enhancing NK/c-Jun activation [131]; conversely, in the arterial endothelium of APP/sw transgenic mice, miR-342-3p was found to directly target the inflammatory factor Chi3l1, exhibiting anti-inflammatory and anti-atherosclerotic effects [132].

The negatively correlated miRNAs listed in (Table 16) are also extensively involved in key AD-related pathological processes, including Aβ production and clearance, tau degradation, synaptic structure and plasticity, neuroinflammation, and oxidative stress. Increased expression of most of these miRNAs, including miR-142-5p, miR-24-3p, and miR-128-5p, exacerbates AD-related pathological damage. Suppression of miR-9-3p expression in the hippocampus leads to impaired hippocampus-dependent memory in mice, suggesting potential neuroprotective effects of miR-9-3p. In contrast, miR-98-5p, miR-9-5p, and miR-128 participate in multiple stages of AD pathology rather than exhibiting simple unidirectional regulation:

1. miR-98-5p is upregulated in APP/PS1 mice and in brain tissue from patients with AD, where it targets Chrna7 to suppress α7 nAChR expression. This weakens cholinergic anti-inflammatory pathways and Ca^2^⁺-dependent synaptic plasticity, thereby exacerbating Aβ deposition and neuroinflammation [134]. Studies in astrocytes and microglia further indicate that miR-98-5p regulates CYP46A1, a key cholesterol-metabolizing enzyme [115], thereby affecting the lipid environment for Aβ clearance, and modulates microglial M1/M2 polarization via the circ_0061183/miR-98-5p/IL-10/TGF-β axis [135];
2. within neurons, miR-9-5p suppresses BACE1 [140, 144] and GSK-3β [141], alleviates oxidative stress, and protects mitochondrial function. However, in late-stage (12-month-old) APPswe/PS1dE9 mice, upregulation of miR-9-5p inhibits the autophagy receptor Optn, thereby obstructing autophagic clearance of Aβ [126]. Additionally, miR-9-5p may impair tau degradation by suppressing UBE4B [143]. Extracellularly, miR-9-5p acts as an endogenous agonist of TLR7/8, inducing neuroinflammation and structural and functional neuronal damage [142];
3. upregulation of miR-128 in dentate gyrus mossy cells inhibits STIM2, leading to impaired synaptic function and memory accuracy [147]. In Aβ-stimulated cortical neurons, miR-128-5p targets PPAR-γ to relieve NF-κB inhibition, thereby amplifying neuroinflammatory and apoptotic responses [149]. Conversely, cellular studies reveal that miR-128-5p suppresses GSK3β, APPBP2, and mTOR expression, reducing tau hyperphosphorylation and Aβ accumulation while promoting autophagic clearance [149]. Among all differentially expressed negatively correlated miRNAs, only miR-138–1-3p exhibits elevated expression in patients with AD within the GSE120584 dataset.

Beyond their direct involvement in downstream pathological processes of AD, the dysregulation of these miRNAs may itself be influenced by more upstream transposable element-related regulation. Analysis using the MDTE database (microRNAs derived from Transposable Elements Database) showed that several of the included miRNAs are derived from transposable elements: miR-28, miR-211 (L2), and miR-511 (L1) were all classified as LINE-derived miRNAs, miR-340 as a TcMar/Mariner DNA transposon-derived miRNA, and miR-342 as a SINE/tRNA-RTE-derived miRNA [151].

Transposable elements (TEs) are DNA sequences capable of moving within the genome, replicating themselves, or being copied into new genomic locations [152]. Emerging evidence indicates that multiple classes of TEs become transcriptionally aberrantly activated during AD pathogenesis under pathological stimuli such as Aβ and tau, thereby triggering neuroinflammatory cascades, altering the expression of target genes, disrupting neuronal homeostasis, and ultimately promoting disease progression [153–155]. Among these, transcriptional activation of LINE-1 (L1) has been linked to tau pathology, and age-related overactivation of LINE-1 has been identified as an important driver of microglial dysfunction [156]. In addition, the large number of G4 structures derived from LINE-1 can disrupt neuronal gene expression, leading to progressive neuronal death and, ultimately, cognitive impairment in AD [157]. HERV/ERV elements have likewise been associated with tau-related pathological changes [155].

Multiple differentially expressed HERV loci have been identified in the brains of patients with AD, and some of these loci are positively correlated with the expression of TLR8 and interferon-stimulated genes [153]. Moreover, a “retrotransposon storm” has been reported in individuals with preclinical AD, characterized by widespread dysregulation of transposable elements in peripheral blood total RNA, including increased LINEs, LTRs, and SVAs and decreased SINEs [158]. In the hippocampus and dorsolateral prefrontal cortex of patients with AD, the RNA processing ratio of Alu elements has also been found to increase in parallel with Aβ pathology. Overactivation of Alu relieves RNA polymerase II (Pol II)-mediated transcriptional repression, resulting in upregulation of target genes enriched in core AD pathogenic pathways and thereby exacerbating neuronal apoptosis [154].

Importantly, TEs and miRNAs are closely interconnected. Current evidence suggests that TEs represent an important evolutionary source of miRNAs and provide an independent noncanonical route for miRNA biogenesis [159], with LINE and SINE elements being the major donors of TE-derived miRNAs in humans [160]. miRNAs also function as key effector molecules through which TEs exert genome-wide epigenetic regulation. By providing cis-regulatory elements for miRNA genes, TEs can modulate miRNA transcription and expression [161]. Conversely, TE-derived miRNAs may silence homologous transposable elements and the genes encoding their corresponding regulatory proteins through sequence complementarity [162, 163].

Taken together, because TEs participate in the formation of miRNA regulatory networks, and because TE-embedded miRNAs in the human brain exhibit marked spatiotemporal dynamic expression patterns during brain development and in neurological conditions such as neurodegenerative diseases [164], we speculate that some of the miRNAs included in this study may carry upstream repeat sequence-related regulatory information. However, this remains a hypothesis and requires further investigation for validation.

### Construction and performance of diagnostic models for AD

Unlike previous exploratory analyses based solely on GSE120584, our study did not perform an agnostic re-screening of all serum miRNAs within the same public cohort. Instead, we used cognitive relevance as the clinical anchor and predefined the candidate set from published clinical studies reporting cognition-associated serum miRNAs in AD. For miRNAs that could not be directly matched, we added same-family or potentially corresponding arm-specific miRNAs for exploratory analysis.

We subsequently constructed a serum-based diagnostic model in the GSE120584 cohort using the expanded panel of candidate miRNAs. Correlation network analysis was used to characterize redundancy among these miRNAs and showed that the overall network topology remained largely stable across correlation thresholds of |ρ|= 0.70–0.90, suggesting the presence of relatively robust co-varying miRNA modules. At |ρ|= 0.80, a large cluster comprising 18 miRNAs emerged. The first principal component (PC1) of this cluster showed only a weak correlation with age, indicating that these miRNAs appear in serum primarily as co-expression modules rather than as individual age-driven markers. On this basis, we applied a “frequency plus cluster de-duplication” strategy within nested cross-validation to identify miRNAs that contributed stably to discrimination beyond age. miR-211-5p was selected in all folds (20/20) and thus represented the most stable signal, followed by miR-128–1-5p and miR-128-3p (each selected in 8/20 folds). Given the modest discriminatory ability of miR-211-5p alone (AUC ≈ 0.62), we interpret its repeated selection as reflecting complementary information to age and consistent performance across resamples rather than strong stand-alone performance. K-AUC curve analysis using the 1-SE rule then showed that the smallest near-optimal miRNA panel depended on the clinical baseline: with age alone as the baseline, the optimal panel size was K = 1 (miR-211-5p), whereas with age plus sex as the baseline, the optimal panel size was K = 3 (miR-211-5p + miR-128–1-5p + miR-128-3p).

Further analysis indicated that, within this cohort, age alone as a clinical baseline variable exhibited substantial discriminative performance (AUC ≈ 0.807), exceeding that of all models based solely on miRNAs (M1 ≈ 0.721, K1 ≈ 0.617, and K3 ≈ 0.700). Incorporation of miRNAs into the age- and sex-based model further increased the AUC to approximately 0.838, indicating that these miRNAs contribute a modest but stable incremental gain. These findings suggest that miR-211-5p and miR-128-related miRNAs are better viewed as adjunctive biomarkers that add limited but stable information beyond baseline clinical variables, rather than as stand-alone replacement markers.

Given the marked heterogeneity of AD in clinical phenotype, pathological burden, and molecular regulation, together with the temporal and spatial overlap among its pathogenic processes [165, 166], circulating cognition-related miRNAs such as miR-211-5p and miR-128 are more plausibly viewed as peripheral readouts of convergent pathology than as linear surrogates of any single pathological event [167]. These miRNAs may reflect the combined effects of Aβ deposition, tau abnormality, neuroinflammation, synaptic dysregulation, and axonal injury. That these miRNAs did not outperform age and sex in the diagnostic models does not diminish their potential relevance; rather, they may capture biologically informative variation that complements baseline clinical variables and aids interpretation of disease heterogeneity and patient stratification [14, 166].

A similarly complex disease network has also been observed in other neurodegenerative disorders. In Parkinson’s disease (PD), for example, large-scale genome-wide association studies (GWAS) and meta-analyses have shown that age at onset is not determined by a single locus or factor, but rather reflects the combined influence of polygenic signals, population genetic background, and disease heterogeneity [168]. At the same time, epidemiological studies suggest that some apparently stable epidemiological associations in PD may still be affected by reverse causation, survival bias, and differences in case composition [169]. Likewise, studies of fluid biomarkers in frontotemporal dementia (FTD) indicate substantial heterogeneity in clinical presentation, genetic background, and pathological basis. Most currently available findings still center on nonspecific biomarkers reflecting neurodegeneration per se, rather than a single disease-specific marker capable of consistently covering all subtypes [170]. Although some peripheral blood miRNAs may be useful for early identification, prognostic evaluation, or stratified management across multiple neurodegenerative diseases, they often lack strict disease specificity and may instead reflect shared processes such as axonal injury, neuroinflammation, and neurodegeneration [93, 165, 170]. For instance, Sheinerman et al. found in AD, FTD, PD, and amyotrophic lateral sclerosis (ALS) that brain-enriched circulating miRNA panels could distinguish each disease from healthy controls while also enabling further differentiation among disease entities, suggesting that peripheral miRNAs may simultaneously capture both shared neurodegenerative processes and disease-specific combinatorial signatures [93].

At the same time, as discussed above, the aberrant changes in the included miRNAs may also be related to abnormal activation of transposable elements (TEs), which commonly accompanies neurodegenerative pathology [171]. With aging, genomic heterochromatin progressively relaxes, and multilayered epigenetic repression mechanisms targeting TEs, including DNA methylation and histone modifications, gradually fail [172, 173]. In addition, characteristic pathological proteins such as tau (in AD/progressive supranuclear palsy) and TDP-43 (in ALS/FTD) participate in TE transcriptional repression, and dysfunction of these proteins may further amplify TE activation [155, 174]. Once aberrantly activated, TEs may promote neurodegenerative pathology through several mechanisms, including induction of DNA double-strand breaks [175], amplification of neuroinflammatory loops via TE-derived nucleic acids in the cytoplasm [176], disruption of transcription and splicing of neuron-critical genes [157], and impairment of mitochondrial structure and function [177].

More specifically, LINE-1/L1 is not only associated with tau pathology in AD, but increased burden of highly active retrotransposition-competent L1s (HA RC-L1s) has also been significantly correlated with PD risk as well as disease progression indicators such as clinical symptom scores and levodopa equivalent dose [178]. In contrast, in the striatum of patients with Huntington’s disease, L1 RNA expression has been reported to be significantly negatively correlated with CAG repeat length [179]. Moreover, abnormal HERV activity has been identified across multiple neurodegenerative diseases in the central nervous system. For example, 698 differentially expressed HERV loci were identified in the parietal cortex of patients with AD [153], whereas in the brains of patients with PD, HERV-K was found to be predominantly localized to astrocytes, and reduced levels were associated with longer disease duration and worsening motor scores [180]. In addition, animal studies have suggested that in ALS/FTD, ERVs may form a self-reinforcing feedback loop with TDP-43 proteinopathy and promote the spread of neurodegeneration through virus-like intercellular propagation [181].

### Possible reasons for the inconsistent differential expression of the included miRNAs between the original studies and GSE120584

We found that most differentially expressed miRNAs showed a downward trend in GSE120584. However, this direction was not fully consistent with their reported correlations with MMSE scores in the included studies or with the pathological roles suggested by previous mechanistic investigations. Two possible explanations may account for this discrepancy.

First, the overall decrease in peripheral blood miRNAs reported in patients with AD in previous studies may have influenced the observed direction of expression for individual miRNAs. For example, Dong H et al. identified 38 differentially expressed miRNAs in 127 patients with AD and 123 controls, of which 28 were downregulated [182]. In plasma exosomes, Lugli G et al. reported 4 upregulated miRNAs and 16 downregulated miRNAs in 70 samples (35 AD cases and 35 controls) [183]. Similarly, Gámez-Valero A et al. found no upregulated miRNAs and only 4 significantly downregulated miRNAs in 10 patients with AD and 15 controls [184].

Second, differences in disease stage, sample processing procedures, detection platforms, and normalization methods may all contribute to inconsistent directions of expression. In the present study, the mean age of patients in the included literature was generally around 72 years, whereas the median age of the AD group in GSE120584 was 80.0 years (interquartile range, 76.0–84.0). This raises the possibility that some of the included miRNAs may show opposite trends across different stages of AD, with increased expression in earlier stages but decreased expression in later stages. For instance, one study found that hippocampal miR-128 was upregulated in the intermediate stage of AD but decreased in the late stage; a similar pattern was also observed for miR-132 and miR-212 in the nucleus basalis of Meynert [185]. In addition, previous studies have suggested that during tissue aging, miRNA biogenesis gradually declines with age, leading to a progressive reduction in miRNA abundance [186]. Therefore, the overall downregulation of the included miRNAs may also be related to the older age of patients with AD in GSE120584.

Moreover, inconsistent expression patterns of the same miRNA across different studies have also been reported. Sun C et al. reviewed 28 miRNA studies related to AD and found that the direction of cortical expression of miR-7 and miR-9 differed across studies [187]. Abidin SZ et al. likewise reported inconsistent differential expression of miR-125b and miR-146a among published studies [188]. In a systematic review, Swarbrick et al. further noted that inconsistencies in miRNA expression in cerebrospinal fluid and peripheral blood of patients with AD may be associated with technical differences across studies as well as variation in disease stage among the sampled populations [11].

It should be noted that these explanations remain speculative at present and require further validation in more standardized datasets.

### Limitations of the study and future directions

This study has several limitations: (1) all included studies were case–control in design and were therefore subject to potential selection and publication biases; moreover, heterogeneity across studies in diagnostic criteria, sample processing, detection platforms, reference controls, and normalization methods may have affected the consistency of miRNA expression directions; (2) in the public dataset GSE120584, the AD and control groups differed markedly in age, sex, and APOE ε4 distribution, and although covariate adjustment was performed, residual confounding cannot be fully excluded; (3) the present study re-evaluated candidate miRNAs in only a single public serum dataset and lacked validation in an independent external cohort; (4) for miRNAs that could not be directly matched, exploratory substitution analysis was performed using miRNAs from the same family or potentially corresponding arm-specific forms, and these results should not be considered equivalent to direct replication of the original miRNAs; and (5) the primary outcome of this study was discrimination between AD and control participants; therefore, the findings cannot be directly extrapolated to early-screening settings such as preclinical AD or conversion from mild cognitive impairment (MCI) to AD.

In light of these limitations and the main findings of the present study, future research should prioritize independent external validation of the proposed miRNA-clinical composite model in prospective, multicenter, large-scale clinical cohorts with pre-standardized procedures. Key methodological steps, including sample collection, processing workflows, detection platforms, reference control selection, normalization strategies, and arm annotation, should be harmonized as much as possible. For miRNAs that cannot be directly matched, repeated measurement using arm-specific assays should be considered to improve comparability and reproducibility. In parallel, peripheral blood, cerebrospinal fluid, and, where appropriate, exosomal samples should ideally be collected from the same individuals to evaluate the cross-fluid consistency of candidate miRNA expression and its relationship with A/T/N biomarkers and central pathological changes.

Because miR-211-5p and miR-128-related miRNAs provided only limited but stable incremental information beyond baseline clinical variables such as age and sex, they are more appropriately viewed as adjunctive rather than replacement biomarkers. Future studies should extend this work to key clinical settings, including preclinical AD, MCI, and conversion from MCI to AD, and should integrate cognitive measures, disease staging, and longitudinal follow-up outcomes to systematically characterize the dynamic behavior of candidate miRNAs across different stages of disease progression. In addition, multimodal modeling incorporating genetic risk, A/T/N biomarkers, imaging measures, and longitudinal cognitive outcomes should be pursued [166, 167]. At the same time, further cell- and animal-based studies are needed to clarify the relationships of miR-211-5p, miR-128, and other key miRNAs with inconsistent expression patterns to Aβ deposition, tau abnormalities, neuroinflammation, synaptic injury, and aberrant activation of transposable elements, thereby providing a stronger evidence base for their potential clinical translation in AD stratification, progression prediction, and adjunctive diagnosis.

## Conclusion

In conclusion, using cognitive relevance as the clinical anchor, we integrated published evidence with validation in a public cohort to identify serum miRNAs associated with cognition in AD. miR-211-5p and a miR-128-related panel provided a modest but consistent improvement when added to age- and sex-based models, with the combination of miR-211-5p, miR-128–1-5p, and miR-128-3p performing best. However, their stand-alone discriminative ability did not exceed that of baseline clinical variables such as age. These markers are therefore better regarded as adjunctive peripheral blood biomarkers rather than stand-alone diagnostic substitutes, and their biological relevance and clinical utility require confirmation in independent external cohorts.

## Supplementary Information

Supplementary Material 1.

Supplementary Material 2.

Supplementary Material 3.

Supplementary Material 4.

Supplementary Material 5.

Supplementary Material 6.

Supplementary Material 7.

Supplementary Material 8.

Supplementary Material 9.

## Data Availability

The data supporting this study were derived from previously published articles and the GEO dataset GSE120584 (https://www.ncbi.nlm.nih.gov/geo/query/acc.cgi), all of which are publicly accessible.
