# Supplementary material for: Literature-derived serum miRNA signatures associated with cognitive decline in Alzheimer’s disease: integrated analysis and machine learning-based diagnostic modeling
Source: Alzheimers Res Ther. 2026 Apr 20;18:135. doi: 10.1186/s13195-026-02048-x (PMC13227807; doi:10.1186/s13195-026-02048-x)
Supplement: Supplementary file 3 — Supplementary Material 3. [file 13195_2026_2048_MOESM3_ESM.docx]

**[Additional file 3] Summary of miRNA Differential Expression and Correlation with MMSE Scores from Included Studies.**

| **Study**  **(Author, Year)** | **miRNA** | **Differential Expression in AD** | | **Correlation with MMSE Score** | |
| --- | --- | --- | --- | --- | --- |
|  |  | **Statistical Test** | **Change (p-value)** | **Method** | **Association (r, p-value)** |
| Xiao JianTing, 2014 [1] | miR-9 | One-way ANOVA | upregulated (P=0.012) | Pearson | Negatively correlated (r=-0.603，P＜0.0001) |
| Tan, L.,2014 [1] | miR-342-3p | two-sided nonparametric Mann-Whitney test | downregulated (P<0.001) | Pearson | Negatively correlated with the Ln–Cq of miR-342-3p. (r=-0.562，P<0.0001) |
| Jia LiHua, 2016 [3] | miR-223 | two-tailed t-tests | downregulated (P<0.001) | Spearman | Positively correlated (r=0.7131，p<0.0001) |
| Qin Yang, 2019 [4] | miR-133b | Student's t-test / ANOVA | downregulated (P<0.001) | Spearman | Positively correlated (r=0.8814，P<0.001) |
| Cao F, 2020 [5] | miR-193a-3p | Student's t-test / ANOVA | downregulated (P<0.001) | Spearman | Positively correlated (r=0.5889，P<.0001) |
| Zhao, Xiaohua, 2020 [6] | miR-28-3p | t-test | upregulated (P<0.05) | Spearman | Negatively correlated (r=-0.6142，P<0.05) |
| Lin Ruidian, 2021 [7] | miR-26b | t-test | downregulated (P<0.05) | Pearson | Positively correlated (r=0.518，p=0.001) |
|  | miR-222 |  | downregulated (P<0.05) |  | Positively correlated (r=0.557，p=0.001) |
| Meng Kaitao, 2021 [8] | miR-128 | Independent samples t-test | upregulated (P<0.05) | Pearson | Negatively correlated (r=-0.571，P<0.05) |
|  | miR-223 |  | downregulated (P<0.05) |  | Positively correlated (r=0.531，P<0.05) |
| Qiao Weidong, 2021 [9] | miR-137 | t-test | downregulated (P<0.01) | Pearson | Positively correlated (r=36.452，P=0.001) |
| Zhang Qun, 2021 [10] | miR-320a | Student's t-test | downregulated (P<0.001) | Pearson | Positively correlated (r=0.651，P<0.001) |
| Zhou Yulei, 2021 [11] | miR-137 | t-test / Z-test | downregulated (P<0.001) | Spearman | Positively correlated (Z/t=-11.210，P<0.01) |
| Dong LiHua, 2021 [12] | miR-202 | one‐way analysis of variance followed by Tukey's multiple comparison test | downregulated (p<0.001) | Spearman | Positively correlated (r=0.7，p<0.001) |
| Liu Qingling, 2021 [13] | miR-331-3p | Student's *t*-test and ANOVA followed by Tukey's test. | downregulated (P<0.001) | Pearson | Positively correlated (r=0.711，P<0.001) |
| Liu, L., 2021 [14] | miR-24-3p | Student’s t-test | upregulated (p<0.001) | Pearson | Negatively correlated (r=-0.617，p<0.001) |
| Zhang, H., 2021 [15] | miR-148a-3p | Student’s t-test | downregulated (P<0.001) | Spearman | Positively correlated (r=0.651，P<0.001) |
| Zhang, M., 2021 [16] | miR-128 | independent sample t-tests | upregulated (P<0.01) | Pearson | Negatively correlated (r=−0.687，P<0.01) |
| Deng Tianling, 2022 [17] | miR-340-5p | t-test | downregulated (P<0.001) | Pearson | Positively correlated (r=0.615，P＜0.001) |
| Wang Wei, 2022 [18] | miR-26b | LSD-t test | downregulated (P<0.001) | Pearson | Positively correlated (r=0.702，P<0.05) |
| Zhou Weihua, 2022 [19] | miR-137 | student-t test | downregulated (P＜0.05) | Pearson | Positively correlated (r=0.319，P＜0.05) |
|  | miR-138 |  | upregulated (P＜0.05) |  | Negatively correlated (r=-0.437，P＜0.05) |
| He Xia, 2023 [20] | miR-98-5p | Independent samples t-test | upregulated (P <0.01) | Pearson / Spearman | Negatively correlated (r=-0.618，P<0.05) |
| Wang, T., 2023 [21] | miR-511-3p | Student's t-test | downregulated (P<0.001) | Pearson | Positively correlated (r=0.765，P<0.001) |
| He Lijie, 2024 [22] | miR-27a-3p | LSD-t test | downregulated (P<0.001) | Spearman | Positively correlated (r=0.424，P<0.001) |
| Wang Pengfei, 2024 [23] | miR-211 | t-test | downregulated (P<0.001) | Spearman | Positively correlated (r=0.539，P＜0.001) |
|  | miR-202 |  | downregulated (P<0.05) |  | Positively correlated (r=0.554，P＜0.001) |

ANOVA: a one-way analysis of variance

**References**

1. Xiao JianTing, Yu Yang, Tan Lan, et al. Changes in serum miR-9 levels and their significance in patients with Alzheimer’s disease [J]. *Shandong Medical Journal*, 2014, 54(38): 8-10.
2. Tan L, Yu JT, Tan MS, Liu QY, Wang HF, Zhang W, Jiang T, Tan L. Genome-wide serum microRNA expression profiling identifies serum biomarkers for Alzheimer's disease. J Alzheimers Dis. 2014;40(4):1017-27. doi: 10.3233/JAD-132144. PMID: 24577456.
3. Jia LH, Liu YN. Downregulated serum miR-223 servers as biomarker in Alzheimer's disease. Cell Biochem Funct. 2016 Jun;34(4):233-7. doi: 10.1002/cbf.3184. Epub 2016 Mar 30. PMID: 27027823.
4. Yang Q, Zhao Q, Yin Y. miR-133b is a potential diagnostic biomarker for Alzheimer's disease and has a neuroprotective role. Exp Ther Med. 2019 Oct;18(4):2711-2718. doi: 10.3892/etm.2019.7855. Epub 2019 Aug 5. PMID: 31572518; PMCID: PMC6755445.
5. Cao F, Liu Z, Sun G. Diagnostic value of miR-193a-3p in Alzheimer's disease and miR-193a-3p attenuates amyloid-β induced neurotoxicity by targeting PTEN. Exp Gerontol. 2020 Feb;130:110814. doi: 10.1016/j.exger.2019.110814. Epub 2019 Dec 16. PMID: 31857133.
6. Zhao X, Wang S, Sun W. Expression of miR-28-3p in patients with Alzheimer's disease before and after treatment and its clinical value. Exp Ther Med. 2020 Sep;20(3):2218-2226. doi: 10.3892/etm.2020.8920. Epub 2020 Jun 22. PMID: 32765698; PMCID: PMC7401892.
7. Lin Ruidian, Zhang Hui. Relationship between serum miR-26b and miR-222 expression and cognitive impairment in patients with Alzheimer’s disease [J]. *Chinese and Foreign Medical Research*, 2021, 19(15): 7-9. DOI:10.14033/j.cnki.cfmr.2021.15.003.
8. Meng Kaitao, Zhang Jianguo, Liu Chong, et al. Correlation analysis of changes in serum miR-128 and miR-223 expression levels with inflammatory response and cognitive function in patients with different severities of Alzheimer’s disease [J]. *Stroke and Nervous Diseases*, 2021, 28(06): 667–671.
9. Qiao Weidong, Chen Xiaoguang, Yun Wang. Expression of serum miR-137 and its relationship with prognosis in patients with Alzheimer’s disease [J]. *Chinese Journal of Neuroimmunology and Neurology*, 2021, 28(01): 25–29.
10. Zhang Qun, Hou Lifang, Zhong Yuehong, Shen Ju. miR-320a as a diagnostic biomarker for Alzheimer’s disease and its protective effect against β-amyloid-induced neurotoxicity [J]. *Advances in Clinical Medicine*, 2021, 11(2): 646–654. DOI:10.12677/ACM.2021.112094.
11. Zhou Yulei, Chen Yanjie, Liu Zemin, et al. Changes in serum miR-137 and ABCA7 levels and their clinical significance in patients with Alzheimer’s disease [J]. *Hebei Medical Journal*, 2021, 27(10): 1652–1657.
12. Dong LH, Sun L, Zhang WJ, Wang XY, Li JM. Reduced serum miR-202 may promote the progression of Alzheimer's disease patients via targeting amyloid precursor protein. Kaohsiung J Med Sci. 2021 Aug;37(8):730-738. doi: 10.1002/kjm2.12391. Epub 2021 May 27. PMID: 34042273; PMCID: PMC11896508.
13. Liu Q, Lei C. Neuroprotective effects of miR-331-3p through improved cell viability and inflammatory marker expression: Correlation of serum miR-331-3p levels with diagnosis and severity of Alzheimer's disease. Exp Gerontol. 2021 Feb;144:111187. doi: 10.1016/j.exger.2020.111187. Epub 2020 Dec 3. PMID: 33279668.
14. Liu L, Liu L, Lu Y, Zhang T, Zhao W. Serum aberrant expression of miR-24-3p and its diagnostic value in Alzheimer's disease. Biomark Med. 2021 Nov;15(16):1499-1507. doi: 10.2217/bmm-2021-0098. Epub 2021 Oct 20. PMID: 34668391.
15. Zhang H, Liu W, Ge H, Li K. Aberrant expression of miR-148a-3p in Alzheimer's disease and its protective role against amyloid-β induced neurotoxicity. Neurosci Lett. 2021 Jun 21;756:135953. doi: 10.1016/j.neulet.2021.135953. Epub 2021 May 9. PMID: 33979697.
16. Zhang M, Han W, Xu Y, Li D, Xue Q. Serum miR-128 Serves as a Potential Diagnostic Biomarker for Alzheimer's Disease. Neuropsychiatr Dis Treat. 2021 Jan 29;17:269-275. doi: 10.2147/NDT.S290925. Erratum in: Neuropsychiatr Dis Treat. 2021 Feb 16;17:513. doi: 10.2147/NDT.S306151. PMID: 33542630; PMCID: PMC7853421.
17. Deng Tianling, Duan Aiqin, Zeng Minling, et al. Correlation between serum miR-340-5p and APPL1 levels and inflammatory response and cognitive function in elderly patients with Alzheimer’s disease [J]. *Journal of Tropical Medicine*, 2022, 22(11): 1551–1556.
18. Wang Wei, Zheng Liping, Chang Liguo, Guo Fushan. Correlation between serum miRNA-26b, Hcy and β-APP levels and disease severity in patients with Alzheimer’s disease and its clinical significance [J]. *International Medicine & Health Guidance News*, 2022, 28(17): 2451–2455. DOI:10.3760/cma.j.issn.1007-1245.2022.17.018.
19. Zhou Weihua, Niu Chengshan, Di Pingwei, et al. Relationship of serum miR-137 and miR-138 expression with cognitive impairment and the PI3K/Akt signaling pathway in peripheral blood lymphocytes in Alzheimer’s disease [J]. *Progress in Modern Biomedicine*, 2022, 22(24): 4674–4678. DOI:10.13241/j.cnki.pmb.2022.24.014.
20. He Xia, Wei Xianhong, Li Jia, et al. Changes in serum miR-98-5p and miR-142-5p levels and their significance in patients with Alzheimer’s disease [J]. *Shandong Medical Journal*, 2023, 63(01): 28–31, 36.
21. Wang T, Zhao W, Liu Y, Yang D, He G, Wang Z. MicroRNA-511-3p regulates Aβ1-40 induced decreased cell viability and serves as a candidate biomarker in Alzheimer's disease. Exp Gerontol. 2023 Jul;178:112195. doi: 10.1016/j.exger.2023.112195. Epub 2023 May 25. PMID: 37121335.
22. He Lijie, Zhang Chunyan, Wang Jing. Correlation of NEAT1 and miR-27a-3p expression in serum and cerebrospinal fluid of patients with Alzheimer’s disease [J]. *Journal of Peking University (Health Sciences)*, 2024, 56(02): 207–212. DOI:10.19723/j.issn.1671-167X.2024.02.002.
23. Wang Pengfei, Chen Changying, Jin Yujuan, et al. Serum expression levels of miR-211 and miR-202 in patients with Alzheimer’s disease and their correlations with cognitive function, anxiety and depressive symptoms [J]. *Journal of Modern Laboratory Medicine*, 2024, 39(02): 129–134.
