# Supplementary material for: Literature-derived serum miRNA signatures associated with cognitive decline in Alzheimer’s disease: integrated analysis and machine learning-based diagnostic modeling
Source: Alzheimers Res Ther. 2026 Apr 20;18:135. doi: 10.1186/s13195-026-02048-x (PMC13227807; doi:10.1186/s13195-026-02048-x)
Supplement: Supplementary file 8 — Supplementary Material 8. [file 13195_2026_2048_MOESM8_ESM.docx]

**[Additional file 8] Roles of differentially expressed, positively correlated miRNAs (included and expanded set) in the pathological process of AD**

| **miRNA** | **Target / Pathways** | | **Experimental Validation** | **Model System** | **Reference** |
| --- | --- | --- | --- | --- | --- |
| **miR-202-3p*** | **functional target** | β-catenin↓ & Gli1↓ | RT-qPCR, Western blot, immunofluorescence double staining, BrdU incorporation | IL-1β-treated Primary rat oligodendrocyte precursor cells (OPCs) | Li Y et al., 2020^[1]^ |
|  | **pathway** | miR-202-3p↑ ⇒ β-catenin↓ ⇒ Gli1↓ ⇒ OPC proliferation↑/differentiation↑ |  |  |  |
| **miR-27a-3p** | **direct target** | GSK3β↓ | Luciferase reporter assay, qPCR, Western blot | Human cerebral microvascular endothelial cell line hCMEC/D3 | Harati R et al., 2022^[2]^ |
|  | **pathway** | miR-27a-3p↑ ⇒ GSK3β↓ ⇒ β-catenin↑ ⇒ claudin-5↑/occludin↑ ⇒ TEER↑、permeability↓ | TEER measurement, FITC-dextran permeability assay, Western blot, qPCR |  |  |
|  | **functional target** | lncRNA NEAT1 could bind to miR-27a-3p | Dual luciferase reporter assay + RNA pull-down experiments | Aβ₁₋₄₀-induced human neuroblastoma SH-SY5Y cells | Dong LX et al., 2021^[3]^ |
|  | **protein levels** | miR-27a-3p↑⇒ BACE1, APP, Aβ, Tau, p-Tau↓ | Western blot + qPCR + flow cytometry | Rat hippocampal Aβ₁₋₄₀ injection |  |
|  | **indirect target** | miR-27a-3p↑consistent with CYP46A1↑ | qPCR | Primary cultured astrocytes from C57BL/6J mice treated with Aβ₁₋₄₂ | Jaberian Asl B et al., 2025^[4]^ |
|  | **pathway** | miR-27a-3p↑⇒ CYP46A1-mediated cholesterol efflux（brain cholesterol clearance） | qPCR | Neonatal C57BL/6J mouse primary astrocytes |  |
| **miR-202-5p*** | **direct target** | APP↓ | Dual-luciferase reporter assay + Western blot | Rat pheochromocytoma PC12 cells | Dong LH et al., 2021^[5]^ |
|  | **pathway** | miR-202-5p↑⇒APP↓→ Aβ↓→ apoptosis↓ | TUNEL apoptosis assay + MTT cell viability assay | Rat pheochromocytoma PC12 cells treated with Aβ |  |
|  | **direct target** | eIF4E↓ | Dual-luciferase reporter assay + RIP assay | Mouse neuroblastoma N2a cells subjected to OGD/R | Li B et al.,2019^[6]^ |
|  | **pathway** | miR-202-5p↑⇒p-Akt, p-GSK-3β, p-c-Raf, p-BAD↑（Akt/GSK-3β signaling pathway） | Western blot | N2a cells + SD rat middle cerebral artery occlusion model |  |
| **miR-137-3p**  **(miR-137)** | **functional target** | TNFAIP1↓ | qPCR + RIP assay | HEK293T cells | Li Y et al.,2022^[7]^ |
|  | **pathway** | miR-137↑⇒TNFAIP1↓⇒ apoptosis↓, autophagy↓↓, amyloidosis↓ | TUNEL apoptosis assay + autophagy fluorescence assay + AlphaLISA amyloidosis assay | Aβ₂₅₋₃₅-induced human neuroblastoma SH-SY5Y cells |  |
|  | **direct target** | TNFAIP1↓ | Dual-luciferase reporter assay + qPCR/Western blot expression validation | mouse neuroblastoma N2a cells + primary mouse cortical neurons | He D et al.,2017^[8]^ |
|  | **pathway** | miR-137↑⇒TNFAIP1↓⇒NF-κB↓ ⇒ apoptosis↓，Caspase-3 ↓（NF-κB signaling pathway） | MTT viability assay + flow cytometry apoptosis assay+Caspase-3 activity assay + ELISA + Western blot | primary neurons and N2a cell model only |  |
|  | **direct target** | PTN↓ | Dual-luciferase reporter assay + qRT-PCR + Western blot | Human neuroblastoma SK-N-SH cells | Yang L et al.,2020^[9]^ |
|  | **pathway** | miR-137↑⇒PTN↓⇒ phosphorylation of PTN/PTPRZ pathway proteins↓⇒apoptosis↓ | Western blot + flow cytometry | SK-N-SH cells |  |
|  | **direct target** | KREMEN1↓ | Dual-luciferase reporter assay + qRT-PCR + Western blot | HEK293 cells + SH-SY5Y cells and human primary neurons | Wang H et al.,2019^[10]^ |
|  | **pathway** | miR-137↑⇒KREMEN1↓⇒ Aβ-induced apoptosis↓， cell viability and MMP↑ | MTT assay + Flow cytometry +JC-1 MMP assay + Western blot | SH-SY5Y cells and human primary neurons |  |
| **miR-26b-5p*** | **direct target** | MME↓ | Dual-luciferase reporter assay + RT-qPCR + Western blot | 293T cells + PC12 cells | Chen L et al.,2025^[11]^ |
|  | **pathway** | miR-26b-5p↓⇒MME↓⇒ Aβ degradation↓⇒apoptosis and oxidative stress↑ | MTT assay + LDH release + Flow cytometry + MDA/SOD/CAT assays + Western blot | rat pheochromocytoma PC12 cells |  |
|  | **functional target** | CYP27B1↓ | qRT-PCR | Primary rat cortical neurons | Dursun E et ^al.,2019[12]^ |
|  | **pathway** | miR-26b-5p↑⇒CYP27B1↓⇒active vitamin D synthesis↓⇒ affects neuronal differentiation and survival | qRT-PCR |  |  |
| **miR-511-3p** | **pathway** | miR-511-3p↑⇒ inflammatory cytokines（IL-1β、IL-6、TNF-α）↓⇒Aβ-induced cell damage and inflammation↓ | ELISA+MTT | Aβ₁₋₄₀ treated SH-SY5Y cells | Wang T et al.,2023^[13]^ |
| **miR-148a-3p** | **direct target** | p35（encoded by CDK5R1） | Dual-luciferase reporter + qRT-PCR + Western blot | HEK293 cells | Zeng L et al.,2021^[14]^ |
|  |  | PTEN |  |  |  |
|  | **pathway** | miR-148a-3p↑⇒p35↓⇒ CDK5 activity↓⇒ p-tau↓ | Western blot+ Co-IP for p35-CDK5 binding | SH-SY5Y with Swedish APP |  |
|  |  | miR-148a-3p↑⇒ PTEN↓⇒ Akt-p↑⇒ CREB-p↑⇒ miR-148a-3p（PTEN/Akt/CREB feed-forward loop） ⇒tau phosphorylation↓ | Western blot + ChIP + promoter-luciferase reporter |  |  |
| **miR-340-5p** | **direct target** | POT1 | Dual-luciferase reporter + qRT-PCR + Western blot | HEK293 cells | Li X et al.,2021^[15]^ |
|  | **pathway** | miR-340-5p↑ ⇒ POT1↓ ⇒ telomerase recruitment↑ ⇒ telomere length↑ ⇒ cellular senescence↓ ⇒ AD symptoms alleviated | TRF + TRAP-ELISA+ SA-β-Gal + behavioral tests | HT22 mouse hippocampal neuronal cells induced with Aβ42 oligomers |  |
|  |  | miR-340-5p↑⇒ POT1↓⇒ telomerase activity↑⇒ telomere length↑⇒ Aβ1–42↓⇒ cognitive improvement | Morris water maze + passive avoidance + TRF + TRAP-ELISA + Aβ1–42 IHC | D-galactose- induced ICR mice |  |
| **miR-222-3p*** | **functional target** | FERMT2↓ | Western blot + APP metabolism functional experimen | HEK293 cells, HeLa cells | Eysert F et al.,2021^[16]^ |
|  | **pathway** | miR-222-3p↑⇒FERMT2↓⇒APP cell surface level↑⇒Aβ secretion↑, sAPPα secretion↑ | Western blot + Cell-surface biotinylation assay + Alpha-LISA assay | HEK293-APP695WT cells；Primary neurons: Postnatal day 0 (P0) rat hippocampal neurons |  |
| **miR-193a-3p** | **direct target** | PTEN↓ | Dual-luciferase reporter + qRT-PCR + Western blot | PC12 rat pheochromocytoma cells / SH-SY5Y human neuroblastoma cells + Aβ25–35 induction | Cao F et al.,2019^[17]^ |
|  | **pathway** | miR-193a-3p↑⇒PTEN↓⇒ PI3K-AKT activity↑⇒cell viability↑, apoptosis↓, alleviates Aβ toxicity | MTT+ Flow cytometry |  |  |
| **miR-223-5p*** | **-** | | | | |
| **miR-331-3p** | **direct target** | Sqstm1 | Dual-luciferase reporter assay + qRT-PCR + Western blot | 293T cells + SH-SY5Y cells + APPswe/PS1dE9 transgenic AD mice | Chen ML et al.,2021^[18]^ |
|  | **pathway** | miR-331-3p↑⇒Sqstm1↓⇒ autophagy activity↓⇒Aβ clearance↓ | Western blot + Immunofluorescence + behavioral tests | APPswe/PS1dE9 transgenic AD mice |  |
|  | **direct target** | VHL | Dual-luciferase reporter assay + qRT-PCR + Western blot | Aβ₁₋₄₀-induced SH-SY5Y human neuroblastoma cells | Liu Q et al.,2020^[19]^ |
|  | **pathway** | miR-331-3p↑⇒VHL↓⇒cell viability↑, inflammation↓ | MTT + ELISA + qRT-PCR |  |  |
| **miR-133b** | **direct target** | EGFR | Dual-luciferase reporter assay + RT-qPCR + Western blot | Aβ₁₋₄₀-induced SH-SY5Y human neuroblastoma cells | Yang Q et al.,2019^[20]^ |
|  | **pathway** | miR-133b↑⇒EGFR↓⇒cell viability↑, apoptosis↓ | MTT + Flow cytometry + RT-qPCR | Aβ₁₋₄₀-induced SH-SY5Y human neuroblastoma cells |  |
| **miR-211-5p*** | **direct target** | Ngn2 | Dual-luciferase reporter assay + qRT-PCR + Western blot | Aβ₁₋₄₂-induced  PC12 rat pheochromocytoma cells | Liu XH et al.,2021^[21]^ |
|  | **pathway** | miR-211↑⇒Ngn2↓⇒PI3K/Akt phosphorylation↓⇒ proliferation↓, apoptosis↑ | Western blot + MTT + Colony formation + TUNEL & Flow cytometry |  |  |
|  | **direct target** | NUAK1 | qRT-PCR + Western blot + Dual-luciferase assay | Neuro2A mouse neuroblastoma cells + Primary mouse cortical neurons（E18.5） + APP/PS1 double transgenic AD mouse model | Fan C et al.,2016^[22]^ |
|  | **pathway** | miR-211-5p↑⇒NUAK1↓⇒ neurite length↓, branching↓ | Immunofluorescence + ImageJ quantification + MTT | Aβ₁₋₄₂-treated primary cortical neuron model |  |
|  | **direct target** | NEP | qRT-PCR + Western blot + dual-luciferase reporter assay | SH-SY5Y human neuroblastoma cells | Chen H et al.,2024^[23]^ |
|  | **pathway** | miR-211-5p↑⇒NEP↓⇒ decreased Aβ clearance⇒ enhanced neurotoxicity | Annexin V/PI staining + Transwell migration assay + ELISA | Aβ1-40-treated SH-SY5Y cell model |  |
|  | **direct target** | SIRT1 | qRT-PCR + Western blot + luciferase reporter assay + immunofluorescence | Rats intracerebroventricular injection of STZ + Neuro-2a: transfected with Aβ1-42 | Zhu R et al.,2020^[24]^ |
|  | **pathway** | miR-211-5p↑⇒SIRT1↓⇒Nrf2↓⇒HO-1↓⇒oxidative stress↑ (MDA/ROS↑, SOD/GSH/GPX↓) | Annexin V/PI staining+ Transwell+ELISA |  |  |
| **miR-342-3p** | **pathway** | miR-342-3p↑⇒JNK/c-Jun activation↑⇒neuronal apoptosis↑⇒Aβ deposition↑⇒ cognitive decline worsens | Western blot + MTT viability, IHC + ELISA (Aβ1-40/42) | 3xTg-AD mice + HT22 cells | Fu Y et al.,2019^[25]^ |
|  | **direct target** | Chi3l1 | qRT-PCR + Western blot + Dual-luciferase assay | APPsw-Tg mice + ApoE-/-mice (atherosclerosis model) + HUVEC/iMAEC/VSMC (human/mouse endothelial and smooth muscle cells) | Jung YY et al.,2018^[26]^ |
|  | **pathway** | miR-342-3p↓⇒Chi3l1↑⇒ VCAM1/ICAM1↑, eNOS↓, NO↓⇒EC inflammation↑; | qPCR + NO assay + THP-1 monocyte adhesion assay + VSMC migration/proliferation assays (BrdU, Wound healing) |  |  |
|  |  | miR-342-3p↓⇒Chi3l1↑⇒PDGF-BB-induced VSMC migration/proliferation↑⇒ atherosclerotic plaque formation |  |  |  |

Note: * miRNAs added through the matching-and-expansion procedure.

**References**

1. Li Y, Liu L, Ding X, Liu Y, Yang Q, Ren B. Interleukin‑1β attenuates the proliferation and differentiation of oligodendrocyte precursor cells through regulation of the microRNA‑202‑3p/β‑catenin/Gli1 axis. Int J Mol Med. 2020 Sep;46(3):1217-1224. doi: 10.3892/ijmm.2020.4648. Epub 2020 Jun 18. PMID: 32582975.
2. Harati R, Hammad S, Tlili A, Mahfood M, Mabondzo A, Hamoudi R. miR-27a-3p regulates expression of intercellular junctions at the brain endothelium and controls the endothelial barrier permeability. PLoS One. 2022 Jan 13;17(1):e0262152. doi: 10.1371/journal.pone.0262152. PMID: 35025943; PMCID: PMC8758013.
3. Dong LX, Zhang YY, Bao HL, Liu Y, Zhang GW, An FM. LncRNA NEAT1 promotes Alzheimer's disease by down regulating micro-27a-3p. Am J Transl Res. 2021 Aug 15;13(8):8885-8896. PMID: 34540002; PMCID: PMC8430163.
4. Jaberian Asl B, Nazeri Z, Pezeshki SP, Kheirollah A, Azizidoost S, Adelipour M, Cheraghzadeh M. Effect of Amyloid Beta on Cholesterol Metabolism-Correlated microRNAs in Primary Cultured Astrocytes of C57BL/6J Mice: A Focus on CYP46A1 and APOE Genes. Cell J. 2025 Mar 22;26(11):625-631. doi: 10.22074/cellj.2025.2029261.1587. PMID: 40235143.
5. Dong LH, Sun L, Zhang WJ, Wang XY, Li JM. Reduced serum miR-202 may promote the progression of Alzheimer's disease patients via targeting amyloid precursor protein. Kaohsiung J Med Sci. 2021 Aug;37(8):730-738. doi: 10.1002/kjm2.12391. Epub 2021 May 27. PMID: 34042273; PMCID: PMC11896508.v
6. Li B, Huang Z, Meng J, Yu W, Yang H. MiR-202-5p attenuates neurological deficits and neuronal injury in MCAO model rats and OGD-induced injury in Neuro-2a cells by targeting eIF4E-mediated induction of autophagy and inhibition of Akt/GSK-3β pathway. Mol Cell Probes. 2020 Jun;51:101497. doi: 10.1016/j.mcp.2019.101497. Epub 2019 Dec 24. PMID: 31877332.
7. Li Y, Jin L, Wang F, Ren L, Pen R, Bo G, Wang L. Epigenetic axis of SNHG19/miR-137/TNFAIP1 modulates amyloid beta peptide 25-35-induced SH-SY5Y cytotoxicity. Epigenomics. 2022 Feb;14(4):187-198. doi: 10.2217/epi-2021-0288. Epub 2022 Feb 16. PMID: 35170354.
8. He D, Tan J, Zhang J. miR-137 attenuates Aβ-induced neurotoxicity through inactivation of NF-κB pathway by targeting TNFAIP1 in Neuro2a cells. Biochem Biophys Res Commun. 2017 Aug 26;490(3):941-947. doi: 10.1016/j.bbrc.2017.06.144. Epub 2017 Jun 24. PMID: 28655611.
9. Yang L, Kang K, Lin Y, Wu Y. Up-regulation of miR-137 can inhibit PTN in target manner to regulate PTN/PTPRZ pathway to prevent cognitive dysfunction caused by propofol. Am J Transl Res. 2020 Nov 15;12(11):7490-7500. PMID: 33312384; PMCID: PMC7724353.
10. Wang H, Lu B, Chen J. Knockdown of lncRNA SNHG1 attenuated Aβ25-35-inudced neuronal injury via regulating KREMEN1 by acting as a ceRNA of miR-137 in neuronal cells. Biochem Biophys Res Commun. 2019 Oct 20;518(3):438-444. doi: 10.1016/j.bbrc.2019.08.033. Epub 2019 Aug 23. PMID: 31447119.v
11. Chen L, Yan X. LncRNA NORAD sponging to miR-26b-5p represses the progression of Alzheimer's disease in vitro by upregulating MME expression. Cytotechnology. 2025 Feb;77(1):41. doi: 10.1007/s10616-024-00691-6. Epub 2025 Jan 10. PMID: 39803415; PMCID: PMC11723864.
12. Dursun E, Candaş E, Yılmazer S, Gezen-Ak D. Amyloid Beta 1-42 Alters the Expression of miRNAs in Cortical Neurons. J Mol Neurosci. 2019 Feb;67(2):181-192. doi: 10.1007/s12031-018-1223-y. Epub 2018 Dec 4. PMID: 30515701.
13. Wang T, Zhao W, Liu Y, Yang D, He G, Wang Z. MicroRNA-511-3p regulates Aβ1-40 induced decreased cell viability and serves as a candidate biomarker in Alzheimer's disease. Exp Gerontol. 2023 Jul;178:112195. doi: 10.1016/j.exger.2023.112195. Epub 2023 May 25. PMID: 37121335.
14. Zeng L, Jiang H, Ashraf GM, Liu J, Wang L, Zhao K, Liu M, Li Z, Liu R. Implications of miR-148a-3p/p35/PTEN signaling in tau hyperphosphorylation and autoregulatory feedforward of Akt/CREB in Alzheimer's disease. Mol Ther Nucleic Acids. 2021 Nov 29;27:256-275. doi: 10.1016/j.omtn.2021.11.019. Erratum in: Mol Ther Nucleic Acids. 2023 Oct 25;34:102059. doi: 10.1016/j.omtn.2023.102059. PMID: 35024240; PMCID: PMC8714918.
15. Li X, Zhang J, Yang Y, Wu Q, Ning H. MicroRNA-340-5p increases telomere length by targeting telomere protein POT1 to improve Alzheimer's disease in mice. Cell Biol Int. 2021 Jun;45(6):1306-1315. doi: 10.1002/cbin.11576. Epub 2021 Mar 19. PMID: 33624913.Li X, Zhang J, Yang Y, Wu Q, Ning H. MicroRNA-340-5p increases telomere length by targeting telomere protein POT1 to improve Alzheimer's disease in mice. Cell Biol Int. 2021 Jun;45(6):1306-1315. doi: 10.1002/cbin.11576. Epub 2021 Mar 19. PMID: 33624913.
16. Eysert F, Coulon A, Boscher E, Vreulx AC, Flaig A, Mendes T, Hughes S, Grenier-Boley B, Hanoulle X, Demiautte F, Bauer C, Marttinen M, Takalo M, Amouyel P, Desai S, Pike I, Hiltunen M, Chécler F, Farinelli M, Delay C, Malmanche N, Hébert SS, Dumont J, Kilinc D, Lambert JC, Chapuis J. Alzheimer's genetic risk factor FERMT2 (Kindlin-2) controls axonal growth and synaptic plasticity in an APP-dependent manner. Mol Psychiatry. 2021 Oct;26(10):5592-5607. doi: 10.1038/s41380-020-00926-w. Epub 2020 Nov 3. Erratum in: Mol Psychiatry. 2021 Oct;26(10):5608. doi: 10.1038/s41380-020-01015-8. PMID: 33144711; PMCID: PMC8758496.
17. Cao F, Liu Z, Sun G. Diagnostic value of miR-193a-3p in Alzheimer's disease and miR-193a-3p attenuates amyloid-β induced neurotoxicity by targeting PTEN. Exp Gerontol. 2020 Feb;130:110814. doi: 10.1016/j.exger.2019.110814. Epub 2019 Dec 16. PMID: 31857133.
18. Chen ML, Hong CG, Yue T, Li HM, Duan R, Hu WB, Cao J, Wang ZX, Chen CY, Hu XK, Wu B, Liu HM, Tan YJ, Liu JH, Luo ZW, Zhang Y, Rao SS, Luo MJ, Yin H, Wang YY, Xia K, Tang SY, Xie H, Liu ZZ. Inhibition of miR-331-3p and miR-9-5p ameliorates Alzheimer's disease by enhancing autophagy. Theranostics. 2021 Jan 1;11(5):2395-2409. doi: 10.7150/thno.47408. Erratum in: Theranostics. 2021 Oct 2;11(20):9774. doi: 10.7150/thno.67227. PMID: 33500732; PMCID: PMC7797673.
19. Liu Q, Lei C. Neuroprotective effects of miR-331-3p through improved cell viability and inflammatory marker expression: Correlation of serum miR-331-3p levels with diagnosis and severity of Alzheimer's disease. Exp Gerontol. 2021 Feb;144:111187. doi: 10.1016/j.exger.2020.111187. Epub 2020 Dec 3. PMID: 33279668.
20. Yang Q, Zhao Q, Yin Y. miR-133b is a potential diagnostic biomarker for Alzheimer's disease and has a neuroprotective role. Exp Ther Med. 2019 Oct;18(4):2711-2718. doi: 10.3892/etm.2019.7855. Epub 2019 Aug 5. PMID: 31572518; PMCID: PMC6755445.
21. Liu XH, Ning FB, Zhao DP, Chang YY, Wu HM, Zhang WH, Yu AL. Role of miR-211 in a PC12 cell model of Alzheimer's disease via regulation of neurogenin 2. Exp Physiol. 2021 Apr;106(4):1061-1071. doi: 10.1113/EP088953. Epub 2021 Mar 2. PMID: 33527539.
22. Fan C, Wu Q, Ye X, Luo H, Yan D, Xiong Y, Zhu H, Diao Y, Zhang W, Wan J. Role of miR-211 in Neuronal Differentiation and Viability: Implications to Pathogenesis of Alzheimer's Disease. Front Aging Neurosci. 2016 Jul 8;8:166. doi: 10.3389/fnagi.2016.00166. PMID: 27458373; PMCID: PMC4937029.
23. Chen H, Huang Z, Lei A, Yu X, Shen M, Wu D. miRNA-211-5p inhibition enhances the protective effect of hucMSC-derived exosome in Aβ1-40 -induced SH-SY5Y cells by increasing NEP expression. J Biochem Mol Toxicol. 2024 Jan;38(1):e23624. doi: 10.1002/jbt.23624. PMID: 38229323.
24. Zhu R, Qi X, Liu C, Wang D, Li L, Liu X, Hou Y, Su X, Lin H. The silent information regulator 1 pathway attenuates ROS-induced oxidative stress in Alzheimer's disease. J Integr Neurosci. 2020 Jun 30;19(2):321-332. doi: 10.31083/j.jin.2020.02.1151. PMID: 32706196.
25. Fu Y, Hu X, Zheng C, Sun G, Xu J, Luo S, Cao P. Intrahippocampal miR-342-3p inhibition reduces β-amyloid plaques and ameliorates learning and memory in Alzheimer's disease. Metab Brain Dis. 2019 Oct;34(5):1355-1363. doi: 10.1007/s11011-019-00438-9. Epub 2019 May 27. PMID: 31134481.
26. Jung YY, Kim KC, Park MH, Seo Y, Park H, Park MH, Chang J, Hwang DY, Han SB, Kim S, Son DJ, Hong JT. Atherosclerosis is exacerbated by chitinase-3-like-1 in amyloid precursor protein transgenic mice. Theranostics. 2018 Jan 1;8(3):749-766. doi: 10.7150/thno.20183. PMID: 29344304; PMCID: PMC5771091.
