# Supplementary material for: Literature-derived serum miRNA signatures associated with cognitive decline in Alzheimer’s disease: integrated analysis and machine learning-based diagnostic modeling
Source: Alzheimers Res Ther. 2026 Apr 20;18:135. doi: 10.1186/s13195-026-02048-x (PMC13227807; doi:10.1186/s13195-026-02048-x)
Supplement: Supplementary file 9 — Supplementary Material 9. [file 13195_2026_2048_MOESM9_ESM.docx]

**[Additional file 9] Roles of differentially expressed, negatively correlated miRNAs (included and expanded set) in the pathological process of AD**

| **miRNA** | **Target / Pathways** | | **Experimental Validation** | **Model System** | **Reference** |
| --- | --- | --- | --- | --- | --- |
| **miR-98-5p** | **direct target** | SNX6 | qRT-PCR + Western blot + luciferase reporter assay + ELISA + MTT viability + flow cytometry | SK-N-SH cels, SH-SY5Y cells  HEK293 cells | Li Q et al., 2016^[1]^ |
|  | **pathway** | miR-98-5p↑ ⇒ SNX6↓ ⇒ BACE1↑ ⇒ sAPPβ↑, βCTF↑ ⇒ Aβ40/42↑ ⇒ apoptosis↑, viability↓ | Western blot + ELISA (Aβ40/42) + MTT (viability) + flow cytometry |  |  |
|  | **direct target** | α7 nAChR↓ | qRT-PCR + Western blot + luciferase reporter assay | HEK293T cells, BV-2 cells  APP/PS1 transgenic mice, C57BL/6J wild-type (WT) mice | Song C et al.,2021^[2]^ |
|  | **pathways** | miR-98-5p↑ ⇒ α7 nAChR↓ ⇒ Ca²⁺ signaling↓ ⇒ CaM/CaMKII↓ ⇒ synaptic proteins↓ ⇒ cognitive deficits↑ | Western blot + immunofluorescence + ROS assay + ELISA, |  |  |
|  |  | miR-98-5p↑ ⇒ α7 nAChR↓ ⇒ NF-κB↑ ⇒ inflammation↑ |  |  |  |
|  |  | miR-98-5p↑ ⇒ α7 nAChR↓ ⇒ Nrf2↓ ⇒ HO-1/NQO-1↓ ⇒ antioxidant↓ |  |  |  |
|  | **direct target** | circ_0061183 (circular RNA) | Dual-luciferase reporter assay | 293T human embryonic kidney cells | Zeng HX et al., 2025^[3]^ |
|  | **pathway** | miR-98-5p↑ ⇒ IL10, BMP2, TGFβR1↓ ⇒ blocks TGF-β signaling ⇒ microglial M2 polarization↓ | qRT-PCR + Western blot | HMC3 human microglial cells |  |
|  | **direct target** | HEY2↓ | Dual-luciferase reporter assay | 293T human embryonic kidney cells | Chen FZ et al., 2018^[4]^ |
|  | **pathway** | miR-98-5p↑ ⇒ HEY2 ↓ ⇒ Jagged1 ↓ & Notch1 ↓ & Hes1/5 ↓ ⇒ APP ↓ & Bax ↓ & Bcl-2 ↑ ⇒ Aβ production ↓ & oxidative stress ↓ & mitochondrial dysfunction ↓ | qRT-PCR + Western blot | Primary hippocampal neurons from AD mice |  |
|  | **functional target** | CYP46A1↓ | qRT-PCR | Aβ₁₋₄₂ treated primary cultured astrocytes from C57BL/6J mice | Jaberian Asl B et al., 2025^[5]^ |
|  | **pathway** | Aβ ⇒ miR-98-5p ↓ ⇒ CYP46A1 ↑ ⇒ 24-hydroxycholesterol ↑ ⇒ brain cholesterol efflux ↑ |  |  |  |
| **miR-142-5p** | **functional target** | PSD-95↓ | qRT-PCR + immunocytochemistry | Aβ₄₂ treated human neuroblastoma SH-SY5Y cells | Song J et al., 2017^[6]^ |
|  | **pathway** | Aβ₄₂ ⇒ miR-142-5p↑ ⇒ (predicted targets AKAP5, DRD1, etc. ↓) ⇒ PSD-95 protein ↓ ⇒ synaptic dysfunction |  |  |  |
|  | **direct target** | BAI3↓ | Dual-luciferase reporter assay | Aβ₁₋₄₂ treated HEK293T human embryonic kidney cells and HT-22 mouse hippocampal neuronal line | Fu CH et al., 2021^[7]^ |
|  | **pathway** | miR-142-5p ↑ ⇒ BAI3 ↓ ⇒ pCaMKII ↓ & PSD-95 ↓ & p-Synapsin ↓ ⇒ synaptic plasticity impaired ⇒ spatial learning/memory deficit | Western blot + immunofluorescence + Morris water maze | APP/PS1 double-transgenic male mice (6–8 months); Aβ₁₋₄₂ treated primary mouse hippocampal neurons; HT-22 cells |  |
|  | **direct target** | PTPN1↓ | Dual-luciferase reporter assay | 293T human embryonic kidney cells; Sprague–Dawley rat single lateral ventricle injection of Aβ₁₋₄₂ | Liang W et al., 2022^[8]^ |
|  | **pathway** | miR-142-5p↑ ⇒ PTPN1 ↓ ⇒ p-Akt/Akt ↓ ⇒ Bax ↑ & Bcl-2 ↓ ⇒ neuronal apoptosis ↑ ⇒ learning/memory impairment | Western blot + IHC + ELISA + TUNEL apoptosis assay |  |  |
| **miR-9-5p*** | **direct target** | OPTN↓ | Dual-luciferase reporter assay | 293T human embryonic kidney cells | Chen ML et al., 2021^[9]^ |
|  | **pathway** | miR-9-5p↑ ⇒ OPTN↓ ⇒ autophagy activity↓ ⇒ Aβ clearance↓ ⇒ Aβ accumulation↑ | Western blot + qRT-PCR + immunofluorescence | SH-SY5Y cells, APPswe/PS1dE9 mice |  |
|  | **direct target** | BACE1↓ | Dual-luciferase reporter assay | Human neuroblastoma SH-SY5Y cells | Ding Y et al., 2021^[10]^ |
|  | **pathway** | miR-9-5p↑ ⇒ BACE1↓ ⇒ C99/C83 ratio↓ ⇒ Aβ deposition↓ ⇒ neurotoxicity ↓ | qRT-PCR + Western blot + CCK-8 + flow cytometry for apoptosis | R/B/Aβ SH-SY5Y cells; C57BL/6 mice (hippocampal injection of Aβ₁₋₄₂ + BDNF-AS knockdown) |  |
|  | **direct target** | GSK-3β | Dual-luciferase reporter assay | Aβ₂₅₋₃₅ treated mouse hippocampal neuronal cell line HT22 | Liu J et al., 2020^[11]^ |
|  | **pathway** | miR-9-5p↑ ⇒ GSK-3β↓ ⇒ stabilized mitochondrial membrane potential, ROS↓, apoptosis↓, Nrf2/Keap1 antioxidant signaling↑ | CCK-8 + flow cytometry for apoptosis + JC-1 mitochondrial potential + ROS fluorescent probe + Western blot |  |  |
|  | **direct ligand** | TLR7 / TLR8↓ | HEK-Blue reporter assay + TNF-ELISA + confocal imaging + RNA-seq + qRT-PCR | human THP-1 macrophages, iPSC-derived human cortical neurons (iNeurons), mouse primary cortical neurons | Kumbol V et al., 2025^[12]^ |
|  | **pathway** | miR-9-5p↑ ⇒ TLR7/8 activation ⇒ TNF/IL-1α/IL-6 expression↑ ⇒ axonal length↓, excitatory synapse VGLUT1↓, neuronal apoptosis↑ | High-content imaging + TUNEL apoptosis assay + Western blot + qRT-PCR | iNeurons (human), human THP-1 macrophages, C57BL/6 and Tlr7−/− mouse primary neurons |  |
|  | **direct target** | UBE4B↓ | miRNA-mRNA pull-down + qRT-PCR + RNAi screen + Western blot | Drosophila S2 cells, adult Drosophila eye neurons, human SH-SY5Y neuroblastoma cells | Subramanian M et al., 2021^[13]^ |
|  | **pathway** | miR-9↑ ⇒ UBE4B↓ ⇒ Tau ubiquitination↓ ⇒ autophagy-mediated Tau degradation↓ ⇒ Tau aggregation↑ ⇒ neurodegeneration↑ | Western blot + autophagy inhibitor treatment + co-immunoprecipitation | SH-SY5Y cells, Tau-BiFC mouse model |  |
|  | **functional target** | BACE1↓ | Western blot + RT-qPCR | Human corneal fibroblasts | Choi SI et al., 2019^[14]^ |
|  | **pathway** | miR-9-5p↑ ⇒ BACE1↓ ⇒ APP β-cleavage↓ ⇒ β-CTF↓ ⇒ AICD↓ ⇒ Aβ production↓ |  |  |  |
| **miR-9-3p*** | **direct target** | Dmd（dystrophin）↓/ SAP97（Dlg1）↓ | luciferase reporter assay + Western blot | mouse hippocampal CA1 neurons, HEK-293T cells | Sim SE et al., 2016^[15]^ |
|  | **pathway** | miR-9-3p↓ ⇒ Dmd/SAP97↑ ⇒ disturbed AMPAR trafficking ⇒ LTP impairment ⇒ spatial & trace memory deficits | hippocampal slice whole-cell patch-clamp LTP/LTD + Morris water maze + object-location memory + trace fear conditioning | adult C57BL/6N mice |  |
| **miR-24-3p** | **direct target** | KLF8↓ | dual-luciferase reporter assay + qRT-PCR | Aβ_25-35_ treated human neuroblastoma SH-SY5Y cells | Liu L et al., 2021^[16]^ |
|  | **pathway** | miR-24-3p↑ ⇒ KLF8↓ ⇒ proliferation↓, apoptosis↑ ⇒ exacerbated AD cell injury | CCK-8 cell viability + Annexin V-FITC/PI flow-cytometry apoptosis assay |  |  |
| **miR-128-3p** | **direct target** | PPARG↓ | miRNA-seq + qRT-PCR + TargetScan prediction + Western blot + luciferase reporter | SAMP8 mouse cortical tissue, primary hippocampal neurons (APP/PS1) | Bellver-Sanchis A et al., 2024^[17]^ |
|  | **pathway** | miR-128-3p↓ ⇒ PPARG↑ ⇒ antioxidant enzymes↑, pro-oxidant enzymes↓ ⇒ OS↓ survival↑ | qRT-PCR + Western blot + LDH survival assay |  |  |
| **miR-128-5p**  **(miR-128-1-5p*)** | **direct target** | STIM2↓ | single-cell qPCR + Western blot + luciferase reporter | 6-month male APP/PS1 mossy cells, HEK293T reporter cells | Deng M et al., 2021^[18]^ |
|  | **pathway** | miR-128-5p↑ ⇒ STIM2↓ ⇒ impaired ER Ca²⁺ sensing ⇒ ↓ glutamate release probability ⇒ MC→SST synaptic failure ⇒ memory imprecision | dual patch-clamp EPSC/PPR + LST touchscreen (behavior) + LNA blocking oligo | 6-month male APP/PS1 mice |  |
|  | **direct target** | GSK-3β↓ | Luciferase reporter assay + Western blot | 293T-Tau cells, SH-SY5Y cells | Li S et al., 2023^[19]^ |
|  | **pathway** | miR-128↑ ⇒ GSK-3β↓ ⇒ Tau phosphorylation↓ | Western blot |  |  |
|  | **direct target** | APPBP2↓ | Luciferase reporter assay + RT-qPCR + Western blot | N2a-APPsw cells, SH-SY5Y cells |  |
|  | **pathway** | miR-128↑ ⇒ APPBP2↓ ⇒ APP↓ ⇒ Aβ↓ | ELISA + Western blot | N2a-APPsw cells |  |
|  | **direct target** | mTOR↓ | Luciferase reporter assay + Western blot | N2a-APPsw cells, SH-SY5Y cells |  |
|  | **pathway** | miR-128↑ ⇒ mTOR↓ ⇒ LC3-II↑ ⇒ autophagy↑ ⇒ Aβ↓ | Western blot + TEM + fluorescence microscopy | N2a-APPsw cells, N2a-tfLC3 cells |  |
|  | **direct target** | PPAR-γ | Luciferase reporter assay + RT-qPCR + Western blot | Primary mouse cortical neurons, Aβ_1–42_ treated Neuro2a cells | Geng L et al., 2018^[20]^ |
|  | **pathway** | miR-128↑ ⇒ PPAR-γ↓ ⇒ NF-κB↑ ⇒ Caspase 3↑ ⇒ apoptosis↑ | MTT assay + Flow cytometry + Caspase 3 & NF-κB activity assay |  |  |
| **miR-138-1-3p*** | **-** | | | | |
| **miR-138-2-3p*** | **-** | | | | |
| **miR-28-3p** | **-** | | | | |

Note: * miRNAs added through the matching-and-expansion procedure.

**Reference**

1. Li Q, Li X, Wang L, Zhang Y, Chen L. miR-98-5p Acts as a Target for Alzheimer's Disease by Regulating Aβ Production Through Modulating SNX6 Expression. J Mol Neurosci. 2016 Dec;60(4):413-420. doi: 10.1007/s12031-016-0815-7. Epub 2016 Aug 19. PMID: 27541017.
2. Song C, Shi J, Xu J, Zhao L, Zhang Y, Huang W, Qiu Y, Zhang R, Chen H, Wang H. Post-transcriptional regulation of α7 nAChR expression by miR-98-5p modulates cognition and neuroinflammation in an animal model of Alzheimer's disease. FASEB J. 2021 Jun;35(6):e21658. doi: 10.1096/fj.202100257R. PMID: 34010470.
3. Zeng HX, Qin SJ, Wu QZ, Zeng QG, Li JH, Oudin A, Kanninen KM, Yang M, Jalava P, Dong GH, Zeng XW. Circular RNA circ_0061183 regulates microglial polarization induced by airborne ultrafine particles in HMC3 cells via sponging miR-98-5p. J Hazard Mater. 2025 Feb 15;484:136719. doi: 10.1016/j.jhazmat.2024.136719. Epub 2024 Nov 29. PMID: 39637799.
4. Chen FZ, Zhao Y, Chen HZ. MicroRNA-98 reduces amyloid β-protein production and improves oxidative stress and mitochondrial dysfunction through the Notch signaling pathway via HEY2 in Alzheimer's disease mice. Int J Mol Med. 2019 Jan;43(1):91-102. doi: 10.3892/ijmm.2018.3957. Epub 2018 Oct 24. PMID: 30365070; PMCID: PMC6257854.
5. Jaberian Asl B, Nazeri Z, Pezeshki SP, Kheirollah A, Azizidoost S, Adelipour M, Cheraghzadeh M. Effect of Amyloid Beta on Cholesterol Metabolism-Correlated microRNAs in Primary Cultured Astrocytes of C57BL/6J Mice: A Focus on CYP46A1 and APOE Genes. Cell J. 2025 Mar 22;26(11):625-631. doi: 10.22074/cellj.2025.2029261.1587. PMID: 40235143.
6. Song J, Kim YK. Identification of the Role of miR-142-5p in Alzheimer's Disease by Comparative Bioinformatics and Cellular Analysis. Front Mol Neurosci. 2017 Jul 18;10:227. doi: 10.3389/fnmol.2017.00227. PMID: 28769761; PMCID: PMC5513939.
7. Fu CH, Han XY, Tong L, Nie PY, Hu YD, Ji LL. miR-142 downregulation alleviates the impairment of spatial learning and memory, reduces the level of apoptosis, and upregulates the expression of pCaMKII and BAI3 in the hippocampus of APP/PS1 transgenic mice. Behav Brain Res. 2021 Sep 24;414:113485. doi: 10.1016/j.bbr.2021.113485. Epub 2021 Jul 21. PMID: 34302879.
8. Liang W, Xie Z, Liao D, Li Y, Li Z, Zhao Y, Li X, Dong M. Inhibiting microRNA-142-5p improves learning and memory in Alzheimer's disease rats via targeted regulation of the PTPN1-mediated Akt pathway. Brain Res Bull. 2023 Jan;192:107-114. doi: 10.1016/j.brainresbull.2022.02.016. Epub 2022 Feb 24. PMID: 35219754.
9. Chen ML, Hong CG, Yue T, Li HM, Duan R, Hu WB, Cao J, Wang ZX, Chen CY, Hu XK, Wu B, Liu HM, Tan YJ, Liu JH, Luo ZW, Zhang Y, Rao SS, Luo MJ, Yin H, Wang YY, Xia K, Tang SY, Xie H, Liu ZZ. Inhibition of miR-331-3p and miR-9-5p ameliorates Alzheimer's disease by enhancing autophagy. Theranostics. 2021 Jan 1;11(5):2395-2409. doi: 10.7150/thno.47408. Erratum in: Theranostics. 2021 Oct 2;11(20):9774. doi: 10.7150/thno.67227. PMID: 33500732; PMCID: PMC7797673.
10. Ding Y, Luan W, Shen X, Wang Z, Cao Y. LncRNA BDNF-AS as ceRNA regulates the miR-9-5p/BACE1 pathway affecting neurotoxicity in Alzheimer's disease. Arch Gerontol Geriatr. 2022 Mar-Apr;99:104614. doi: 10.1016/j.archger.2021.104614. Epub 2021 Dec 29. PMID: 34990931.
11. Liu J, Zuo X, Han J, Dai Q, Xu H, Liu Y, Cui S. MiR-9-5p inhibits mitochondrial damage and oxidative stress in AD cell models by targeting GSK-3β. Biosci Biotechnol Biochem. 2020 Nov;84(11):2273-2280. doi: 10.1080/09168451.2020.1797469. Epub 2020 Jul 25. PMID: 32713252.
12. Kumbol V, Ivanov A, McGurran H, Schüler J, Zhai Y, Ludwik K, Hinkelmann L, Brehm M, Krüger C, Küchler J, Wallach T, Höltje M, Beule D, Stachelscheid H, Lehnardt S. Neurodegenerative disease-associated microRNAs acting as signaling molecules modulate CNS neuron structure and viability. Cell Commun Signal. 2025 Apr 24;23(1):196. doi: 10.1186/s12964-025-02199-8. PMID: 40275260; PMCID: PMC12020182.
13. Subramanian M, Hyeon SJ, Das T, Suh YS, Kim YK, Lee JS, Song EJ, Ryu H, Yu K. UBE4B, a microRNA-9 target gene, promotes autophagy-mediated Tau degradation. Nat Commun. 2021 Jun 2;12(1):3291. doi: 10.1038/s41467-021-23597-9. Erratum in: Nat Commun. 2021 Jul 7;12(1):4257. doi: 10.1038/s41467-021-24572-0. PMID: 34078905; PMCID: PMC8172564.
14. Choi SI, Lee B, Woo JH, Jeong JB, Jun I, Kim EK. APP processing and metabolism in corneal fibroblasts and epithelium as a potential biomarker for Alzheimer's disease. Exp Eye Res. 2019 May;182:167-174. doi: 10.1016/j.exer.2019.03.012. Epub 2019 Mar 28. PMID: 30930125.
15. Sim SE, Lim CS, Kim JI, Seo D, Chun H, Yu NK, Lee J, Kang SJ, Ko HG, Choi JH, Kim T, Jang EH, Han J, Bak MS, Park JE, Jang DJ, Baek D, Lee YS, Kaang BK. The Brain-Enriched MicroRNA miR-9-3p Regulates Synaptic Plasticity and Memory. J Neurosci. 2016 Aug 17;36(33):8641-52. doi: 10.1523/JNEUROSCI.0630-16.2016. PMID: 27535911; PMCID: PMC6601897.
16. Liu L, Liu L, Lu Y, Zhang T, Zhao W. Serum aberrant expression of miR-24-3p and its diagnostic value in Alzheimer's disease. Biomark Med. 2021 Nov;15(16):1499-1507. doi: 10.2217/bmm-2021-0098. Epub 2021 Oct 20. PMID: 34668391.
17. Bellver-Sanchis A, Ávila-López PA, Tic I, Valle-García D, Ribalta-Vilella M, Labrador L, Banerjee DR, Guerrero A, Casadesus G, Poulard C, Pallàs M, Griñán-Ferré C. Neuroprotective effects of G9a inhibition through modulation of peroxisome-proliferator activator receptor gamma-dependent pathways by miR-128. Neural Regen Res. 2024 Nov 1;19(11):2532-2542. doi: 10.4103/1673-5374.393102. Epub 2024 Jan 8. PMID: 38526289; PMCID: PMC11090428.
18. Deng M, Zhang Q, Wu Z, Ma T, He A, Zhang T, Ke X, Yu Q, Han Y, Lu Y. Mossy cell synaptic dysfunction causes memory imprecision via miR-128 inhibition of STIM2 in Alzheimer's disease mouse model. Aging Cell. 2020 May;19(5):e13144. doi: 10.1111/acel.13144. Epub 2020 Mar 28. Erratum in: Aging Cell. 2021 Mar;20(3):e13327. doi: 10.1111/acel.13327. PMID: 32222058; PMCID: PMC7253057.
19. Li S, Poon CH, Zhang Z, Yue M, Chen R, Zhang Y, Hossain MF, Pan Y, Zhao J, Rong L, Chu LW, Shea YF, Rogaeva E, Tu J, St George-Hyslop P, Lim LW, Song YQ. MicroRNA-128 suppresses tau phosphorylation and reduces amyloid-beta accumulation by inhibiting the expression of GSK3β, APPBP2, and mTOR in Alzheimer's disease. CNS Neurosci Ther. 2023 Jul;29(7):1848-1864. doi: 10.1111/cns.14143. Epub 2023 Mar 7. PMID: 36880288; PMCID: PMC10324361.
20. Geng L, Zhang T, Liu W, Chen Y. Inhibition of miR-128 Abates Aβ-Mediated Cytotoxicity by Targeting PPAR-γ via NF-κB Inactivation in Primary Mouse Cortical Neurons and Neuro2a Cells. Yonsei Med J. 2018 Nov;59(9):1096-1106. doi: 10.3349/ymj.2018.59.9.1096. PMID: 30328325; PMCID: PMC6192880.
